# Biochemical phenotyping unravels novel metabolic abnormalities and potential biomarkers associated with treatment of GLUT1 deficiency with ketogenic diet

**DOI:** 10.1371/journal.pone.0184022

**Published:** 2017-09-29

**Authors:** Gerarda Cappuccio, Michele Pinelli, Marianna Alagia, Taraka Donti, Debra-Lynn Day-Salvatore, Pierangelo Veggiotti, Valentina De Giorgis, Simona Lunghi, Maria Stella Vari, Pasquale Striano, Nicola Brunetti-Pierri, Adam D. Kennedy, Sarah H. Elsea

**Affiliations:** 1 Department of Translational Medicine, Sector of Pediatrics, Federico II, University, Naples, Italy; 2 Telethon Institute of Genetics and Medicine, Pozzuoli, Naples, Italy; 3 Department of Molecular and Human Genetics, Baylor College of Medicine, One Baylor Plaza, Houston, TX, United States of America; 4 Greenwood Genetic Center, Greenwood, South Carolina, United States of America; 5 Department of Medical Genetics & Genomic Medicine, Saint Peter’s University Hospital, New Brunswick, New Jersey, United States of America; 6 Department Brain and Behavioral Sciences Fondazione IRCCS C, Mondino, Italy; 7 Pediatric Neurology and Muscular Diseases Unit, Department of Neurosciences, Rehabilitation, Ophthalmology, Genetics, Maternal and Child Health, University of Genoa, ‘G, Gaslini’ Institute, Genova, Italy; 8 Metabolon, Morrisville, North Carolina, United States of America; Mayo Clinic Rochester, UNITED STATES

## Abstract

Global metabolomic profiling offers novel opportunities for the discovery of biomarkers and for the elucidation of pathogenic mechanisms that might lead to the development of novel therapies. GLUT1 deficiency syndrome (GLUT1-DS) is an inborn error of metabolism due to reduced function of glucose transporter type 1. Clinical presentation of GLUT1-DS is heterogeneous and the disorder mirrors patients with epilepsy, movement disorders, or any paroxysmal events or unexplained neurological manifestation triggered by exercise or fasting. The diagnostic biochemical hallmark of the disease is a reduced cerebrospinal fluid (CSF)/blood glucose ratio and the only available treatment is ketogenic diet. This study aimed at advancing our understanding of the biochemical perturbations in GLUT1-DS pathogenesis through biochemical phenotyping and the treatment of GLUT1-DS with a ketogenic diet. Metabolomic analysis of three CSF samples from GLUT1-DS patients not on ketogenic diet was feasible inasmuch as CSF sampling was used for diagnosis before to start with ketogenic diet. The analysis of plasma and urine samples obtained from GLUT1-DS patients treated with a ketogenic diet showed alterations in lipid and amino acid profiles. While subtle, these were consistent findings across the patients with GLUT1-DS on ketogenic diet, suggesting impacts on mitochondrial physiology. Moreover, low levels of free carnitine were present suggesting its consumption in GLUT1-DS on ketogenic diet. 3-hydroxybutyrate, 3-hydroxybutyrylcarnitine, 3-methyladipate, and N-acetylglycine were identified as potential biomarkers of GLUT1-DS on ketogenic diet. This is the first study to identify CSF, plasma, and urine metabolites associated with GLUT1-DS, as well as biochemical changes impacted by a ketogenic diet. Potential biomarkers and metabolic insights deserve further investigation.

## Introduction

Metabolomic profiling is a semi-quantitative, unbiased analysis that looks at perturbations in metabolism. Metabolomics can be used for the diagnosis of inborn errors of metabolism (IEMs) enabling the simultaneous analysis of ~800 metabolites (amino acids, organic acids, fatty acids, neurotransmitters, nucleotides, cofactors and vitamins, bile acids, and other molecules <1,000 Da in molecular weight) within a single analysis [1]. Moreover, it is emerging as a powerful tool to understand small molecule perturbations occurring because of an enzyme defect and the relationship to the disease pathogenesis [2,3].

GLUT1 deficiency syndrome (GLUT1-DS) is an autosomal dominant, treatable neurological disorder due to a deficiency of glucose transporter type 1 (GLUT1) with an estimated frequency at approximately 1: 83,000 [4]. The GLUT1 transporter is encoded by the solute carrier family 2 member 1 (*SLC2A1*) gene and *SLC2A1* heterozygous pathogenic mutations cause GLUT1-DS. GLUT1 specifically transports glucose across the blood-brain barrier; only five percent of glucose is transported across the blood-brain barrier by passive diffusion [5]. Deficiency of GLUT1 results in hypoglycorrhachia and impairment of cerebral metabolism. Neurons in the superficial layers of cerebral cortex and hippocampus followed by neurons in basal ganglia and thalamus are primarily affected by lack of glucose. Hypoglycemia-induced neuronal death is not a direct result of energy failure but involves additional mechanisms such as glutamate neurotoxicity or toxic levels of aspartate and/or adenosine [5–7].

Untreated GLUT1-DS has a broad clinical presentation comprising intellectual disability, movement disorder, acquired microcephaly and seizures. Recently, dominant *SLC2A1* mutations were found in various forms of epilepsy, including genetic generalized epilepsy (GGE). In clinical practice, the clinically available distinctive biomarker for GLUT1-DS is a low concentration of glucose in cerebrospinal fluid (CSF) (<50 mg/dl or CSF-to-blood glucose ratio <0.60). Early diagnosis is critical for an effective etiological therapy.

Among IEMs, GLUT1-DS is unique because it is treatable and earlier treatment results in a better prognosis. However, it is not routinely tested by newborn screening. GLUT1-DS treatment is based on ketogenic diet (KD), an isocaloric, high fat, low-carbohydrate diet inducing production of ketone bodies (beta-hydroxybutyrate and acetoacetate) mimicking the biochemical changes occurring in periods of fasting. KD is also used as adjuvant therapy in patients with drug-resistant epilepsy [6, 7]. The diet consists of 3–4 g of fat to every 1 g of carbohydrate and protein combined (classic 3:1 or 4:1 KD). Although KD is the first choice of treatment for GLUT1-DS, in about 20% of patients, this diet can lose its effectiveness over time [7]. Further studies on pathophysiological mechanisms of KD are needed to develop novel therapeutic strategies.

In this study, we describe the identification of biomarkers associated with GLUT1-DS plus KD. We initially performed ultra-high performance liquid chromatography-tandem mass spectrometry on a single CSF sample from a subject diagnosed with GLUT1-DS and performed subsequent analyses on subjects diagnosed with GLUT1-DS and using KD intervention. Due to timing of metabolomics platform advancements, two different configurations were used, but consistent biochemical alterations were identified between the methods that aligned with the changes in phenotype and clinical appearance of the patients. To our knowledge, this is the first application of metabolomics in patients diagnosed with GLUT1-DS.

## Material and methods

### Participants and clinical data

Nine patients with a diagnosis of GLUT1-DS were enrolled in the study based on CSF glucose-to–blood glucose ratio <0.6 and the presence of a pathogenic *SLC2A1* mutation. 8 Patients with GLUT1-DS were referred to our clinical laboratory for biochemical testing or were enrolled in our study at the Department of Child Neurology and Psychiatry, Fondazione IRCCS Istituto Neurologico C. Mondino in Pavia, Italy (patients previously published [8]) while the 9^th^ patient has been followed by the Department of Medical Genetics & Genomic Medicine, Saint Peter’s University Hospital, New Brunswick, USA. Table 1 outlines demographics, clinical, biochemical and molecular information for each of the patients, as well as the ketogenic diet intervention for each patient at the time the biofluid was obtained. Three of the nine patients were not on ketogenic diet, while the remaining six patients were on KD. For the three patients, not on KD, only CSF samples were available.

**Table 1:** Clinical features of patients with GLUT1-DS.

Pt	Sample ID	Gender	Age (years)*	CSF / Plasma Glucose	SLC2A1 variant NM_006516.2	HC (%)	IQ	Seizure onset (months) and type	MD	Other clinical features	AEDs	KD type
*1*	*BIEM-00796* *Plasma* *BIEM-00797* *Urine*	*M*	*11*	*0*.*51*	*p*.*Arg223Trp*	*<25*	*82*	*30*, *FCS*	*PED*	*M*	*OXC*	*2*:*1*
*2*	*BIEM-00784 Plasma* *BIEM-00785 Urine*	*F*	*2*	*0*.*37*	*p*.*Arg249Ala fs131X*	*25–50*	*50*	*9*, *MAS*	*/*	*/*	*/*	*1*.*6*:*1*
*3*	*BIEM-00786* *Plasma* *BIEM-00787* *urine*	*F*	*7*	*NA*	*p*.*Asn34Ser*	*>50*	*93*	*NA*, *ABS*	*/*	*/*	*ETS*	*3*:*1*
*4*	*BIEM-00791* *plasma* *BIEM-00792* *Urine*	*F*	*7*	*0*.*36*	*p*.*Leu124TrpfsX12*	*>50*	*55*	*6*, *MS*	*/*	*O*	*CLB*	*3*:*1*
*5*	*BIEM-00788 Plasma*	*F*	*13*	*0*.*42*	*p*.*Thr295Met /pT295M9*	*25*	*79*	*24*, *ABS*	*PED*	*O*	*VPA ETS*	*4*:*1*
*6*	*BIEM-00789* *plasma* *BIEM-00790* *Urine*	*M*	*19*	*0*.*39*	*p*.*Arg223Thr*	*<10*	*50*	*17*, *MAS*	*/*	*O*	*LEV VPA*	*2*:*1*
*7*	*BIEM-00782* *plasma* *BIEM-00783* *CSF*	*M*	*10*	*0*.*5*	*p*.*Leu67Pro*	*>50*	*99*	*/*	*PED*	*W*	*/*	*/*
*8*	*BIEM-00794* *plasma* *BIEM-00795* *CSF*	*F*	*12*	*NA*	*p*.*Thr9Met*	*>50*	*102*	*84*, *ABS*	*/*	*/*	*ETS- started after CSF sampling*	*/*
*9*	*604266 CSF*	*M*	*Newborn*	*0*.*32*	*p*.*Arg153Gly*	*20*	*/*	*9 days (0*.*3 months)*, *FCS*	*/*	*small*, *unilateral preauricular tag*	*LEV- started after CSF sampling*	

Clinical and molecular detail for each patient are depicted in Table 1.

*F*. *female; M*. *male; Ratio*. *CSF/blood glucose ratio; NA*. *not available; Y*. *yes; N*. *no; HC*. *head circumference; m*. *months; ABS*. *absence seizure; FCS*. *Focal complex seizures; MAS*. *myoclonic atonic seizures*. *MS*. *myoclonic seizures; MD*. *movement disorder; C*. *chronic; PED*. *paroxismal exertion-induced dyskinesia; M*. *migraine; O oculogiric crises; W*. *weakness; KD*. *ketogenic diet; OXC*. *oxacarbazepine; ETS*. ethosuximide; CLB. Clobazam; VPA. valproic acid; LEV. Levetiracetam.

* Age at diagnosis of disease

This study received institutional review board approval at either Baylor College of Medicine or at Federico II University, where the investigations complied with the Declaration of Helsinki, and parents provided written informed consent before the beginning the study. All patients underwent a lumbar puncture in the fasting state (after 5–6 h of fasting) either as part of standard medical care or as part of this the initial enrollment in this study. Blood samples for glucose measurement were obtained immediately before the lumbar procedure to avoid stress-related hyperglycemia. A CSF-to–blood glucose ratio <0.6 was considered suspicious for GLUT1-DS. Confirmation of the diagnosis was obtained by *SLC2A1* mutation analysis.

### Molecular analysis

Molecular analysis of SLC2A1 was performed clinically or under a research protocol, including Sanger sequencing of all exons and flanking donor/acceptor sequences, as well as deletion/duplication analysis via multiplex ligation-dependent probe amplification (MLPA) as previously described [4].

### Global metabolomic profiling

Metabolomic profiling was performed by Baylor Genetics Laboratories (Houston, TX) and Metabolon, Inc (Durham, NC) as described previously [1,9,10] for plasma, urine, and CSF using two different platform configurations. For the both configurations, small molecules were extracted from 100 of ul of sample in an 80% methanol solution.

The first platform configuration consisted of four chromatographic analyses: GC-MS, LC-MS/MS in positive mode (LCMS Pos), LC-MS/MS in negative mode (LCMS Neg), and a LC-MS/MS Polar method (LCMS Pol). GC-MS was performed on bistrimethyl-silyl-trifluoroacetamide derivatized analytes using a Trace DSQ fast-scanning single-quadrupole mass spectrometer (Thermo-Finnigan). For LC/MS Neg and LCMS Pos methods, chromatographic separation was completed using an ACQUITY UPLC (Waters) equipped with a Waters BEH C18 column followed by analysis with an Orbitrap Elite high resolution mass spectrometer (Thermo-Finnigan).

The second configuration utilized the same mass spectrometry methods but utilized the following chromatographic methods: LCMS Neg (same as the previous configuration), LCMS Pol (same as previous configuration), and LCMS positive ion method focusing on lipophilic compounds (LCMS Pos Lipid) and LCMS positive ion method focusing on polar compounds (LCMS Pos Polar). In both configurations, metabolites were identified with known chemical structure by matching the ions’ chromatographic retention index, accurate mass, and mass spectral fragmentation signatures with reference library entries created from authentic standard metabolites under the identical analytical procedure as the experimental samples [11]. Currently, the reference library contains entries for ~2500 unique human metabolites.

Semi-quantitative analysis was achieved by comparing patient samples to a set of invariant anchor specimen included in each batch. Raw spectral intensity values were normalized to the anchor samples log transformed and compared to a normal reference population to generate z-score values. The reference population was a pediatric, randomly sampled population that was matched for age, diet and gender for this comparison. Rare compounds are those analytes detected in the patient specimen but only rarely seen in the reference population (<5% of all patients tested). Median raw intensity values were calculated for all analytes identified in ≥2/3 of the anchor specimen and these median values were then used to normalize corresponding analyte raw intensity values in patient specimen. Analytes not identified in 2/3 or more of the anchor specimens were excluded from analysis. Anchor adjusted analyte values were scaled to the median anchor adjusted analyte values of all previous specimens analyzed to date, missing values were imputed using the minimum detected value and the data were log-transformed. Data collected from samples were normalized to creatinine. Z-scores were calculated using the mean and standard deviation of the entire median-scaled log-transformed dataset.

### Statistical analysis

In the single-metabolite analysis, we assessed whether study-subject metabolite levels were altered compared to background by using single-group t-test on z-scores. In the pathway analysis, we assessed whether multiple metabolite levels within a pathway were altered. To this aim, we calculated the mean values for all metabolites and applied a Kruskal-Wallis analysis comparing the mean levels of pathway-metabolites versus non-pathway-metabolites. Statistical analysis was performed by using the R package for statistical computing version 3.3.1. Analysis with p-values below 0.001 were considered as statistically significant. Box-plots were designed with ggplot2 R-package: the solid line within the box represents the median; top and bottom lines of the box represent 75th and 25th percentiles respectively; top and bottom ends of whiskers represent the 75th percentile plus the 1.5 * Inter-Quartile Range (IQR) and 25th percentile minus the 1.5*IQR respectively; dots outside the whiskers represents outlier values.

## Results

### Metabolomic studies in GLUT1-DS CSF

**Fig 1:**
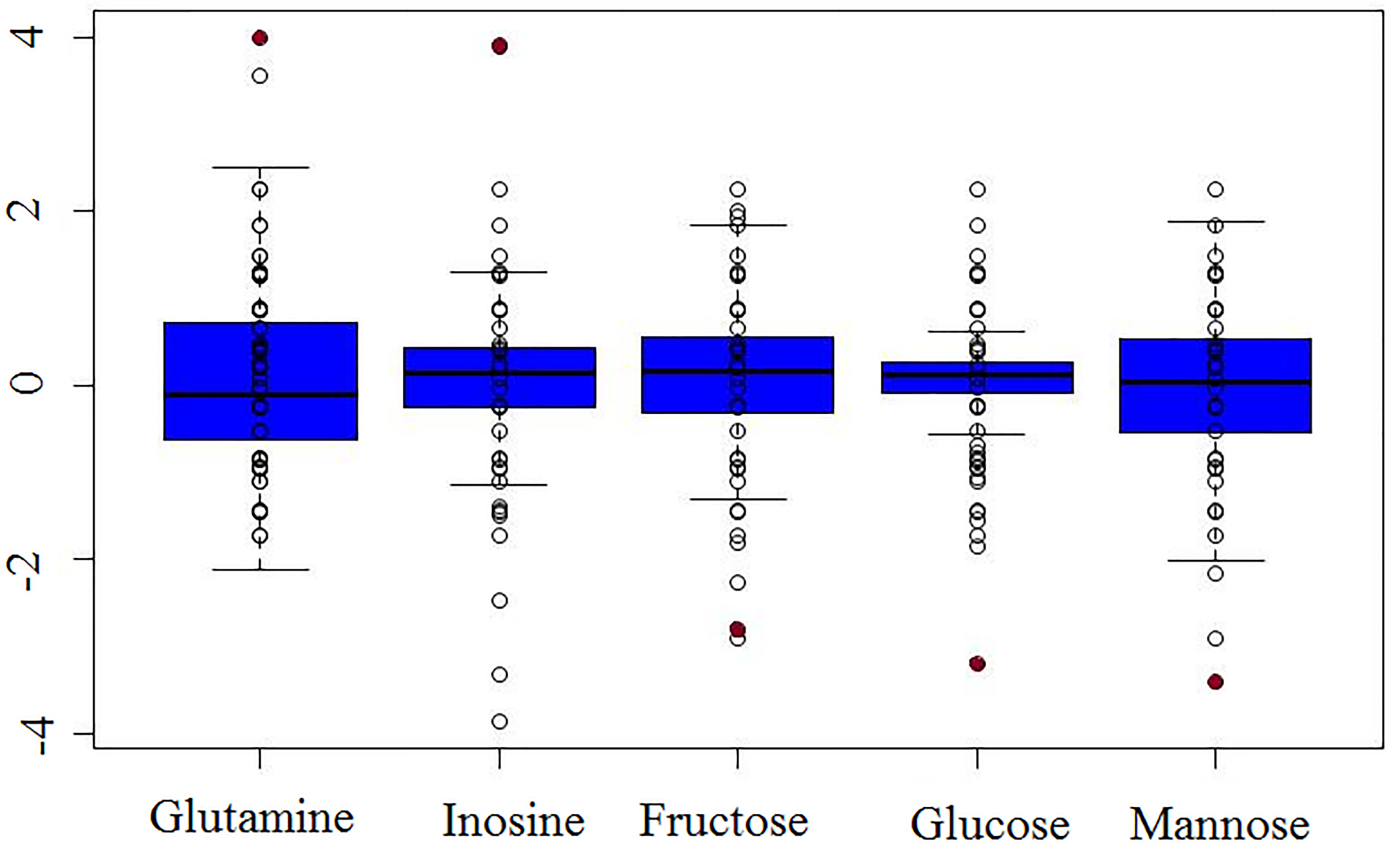
Metabolomic profiling identifies significantly altered levels of molecules in cerebrospinal fluid. Analytes significantly outside of the normal range are reported (relative to the general population. Z-scores <-2 or >2 are considered significant. A boxplot demonstrating analytes from the affected patient (represented by red circle) identified in the metabolomics profile with significant deviation from the normal range are shown. Elevations of glutamine (Z-score +3.98) and inosine (Z-score +3.91), along with reductions of glucose (Z-score -3.2), fructose (Z-score -2.8), and mannose (Z-score -2.86), are indicated. A complete listing of significantly altered analytes identified in the metabolomics analysis is provided in S1, S2 and S3 Tables.

Molecules with Z-scores above or below ±2 are listed in S1 Table. Metabolomic analysis identified 292 compounds in the CSF of Patient 9, of which 38 had significant Z-score values >2 or <-2. The most significant results were the detection of low levels of fructose (Z-score –2.8), mannose (Z-score –2.86) and glucose (Z-score –3.2), and high levels of glutamine (Z-score +3.98) and inosine (Z-score +3.91) (Fig 1). We performed traditional clinical CSF amino acid evaluation on the same CSF sample submitted for metabolomic profiling; however, no amino acid abnormalities were detected (glutamine 455.144 nM/ml, normal range 161–533 and glutamic acid 0 nM/ml, normal range 0–117).

By the time additional CSF samples were acquired, two in total, from subjects with GLUT1-DS, there was a change in the metabolomics platform configuration; the information regarding the version of the platform utilized for each sample is outlined in Table 2. There was a consistent profile observed (Table 2) with 321 and 322 named metabolic compounds identified. Comparing the biochemical profiles for these two patients to our reference population revealed significantly lower glycerol 3-phosphate (Gro3P) levels and significantly elevated isocitrate levels (Table 2). No significant changes in amino acids or neurotransmitters were found in the CSF from these two subjects.

**Table 2:** Z-scores of carbohydrate levels in CSF from patients diagnosed with GLUT1-DS not being treated with a KD.

Platform Version	Version 1	Version 2	
Carbohydrate	604266 (Patient 9)	BIEM-00783 (Patient 7)	BIEM-00795 (Patient 8)
Glucose	-3.3	-0.69	-0.61
Fructose	-2.8	-0.03	-1.11
Mannose	-2.86	0.15	0.02
Glycerol 3-phosphate	ND	-3.69	-2.34
Isocitrate	ND	1.85	2.20

ND–Not detected

### Metabolomic studies on plasma samples from GLUT1-DS patients on ketogenic diet (KD)

Metabolomic analyses of plasma from the six GLUT1-DS patients on KD identified an average of 633 metabolites. Each patient’s ketogenic diet intervention is outlined in Table 1. The examination of the Z-scores for each patient revealed several biochemical elevated across most of the plasma samples (Table 3). These include 3-hydroxybutyrate and 3-hydroxybutyrylcarnitine as well as 3-methyladipate and N-acetylglycine. All plasma samples had matched urine samples and 3-hydroxybutyrate, 3-methyladipate, and N-acetylglycine showed elevated levels in urine (Table 3). Changes associated with individual carbohydrates did not show Z-scores <-2, but several molecules did show decreased levels in plasma (Table 4).

**Table 3:** Z-scores for consistently elevated compounds in plasma and urine from GLUT1-DS on KD diet.

Individual Patient	Sample ID	Matrix	3-hydroxybutyrate	3-hydroxy butyrylcarnitine	3-methyl adipate	N-acetyglycine
1	BIEM-00796	EDTA Plasma	1.11	1.36	1.37	1.58
	BIEM-00797	Urine	3.64	0.91	0.85	-0.59
2	BIEM-00784	EDTA Plasma	3.22	2.13	1.63	2.25
	BIEM-00785	Urine	4.93	1.04	1.95	1.72
3	BIEM-00786	EDTA Plasma	3.59	2.82	2.96	2.39
	BIEM-00787	Urine	6.36	0.42	2.38	3.15
4	BIEM-00791	EDTA Plasma	3.36	2.31	2.09	1.99
	BIEM-00792	Urine	6.19	0.72	1.75	3.98
5	BIEM-00788	EDTA Plasma	3.38	1.77	2.24	2.50
6	BIEM-00789	EDTA Plasma	2.40	1.56	2.55	3.68
	BIEM-00790	Urine	4.09	0.07	2.36	1.03
7	BIEM-00783	CSF	Sample acquired prior to initiation of KD			
	BIEM-00782*	Not EDTA Plasma	NA	NA	NA	NA
8	BIEM-00795	CSF	Sample acquired prior to initiation of KD			
	BIEM-00794	Urine	0.04	0.59	0.94	1.45
	BIEM-00793*	Improperly handled EDTA Plasma	NA	NA	NA	NA

* Sample not included in analysis but listed for completion. Results leading to non-EDTA plasma and/or improper handling conclusion identified after data acquisition.

**Table 4:** Carbohydrate levels in plasma from patients diagnosed with GLUT1-DS and taking a KD.

Carbohydrate	Patient 1	Patient 2	Patient 3	Patient 4	Patient 5	Patient 6
Maltose	-1.380	-1.376	-1.377	-1.375	-1.379	-1.379
Sucrose	-1.279	-1.275	-1.279	-1.276	-1.279	-1.275
Lactose	-0.534	-0.533	-0.535	-0.530	-0.535	-0.535
Mannitol/sorbitol	-1.681	-1.477	-1.544	-0.757	-1.327	-1.606
N-acetylglucoseamine / N-acetylgalactosamine	0.453	0.801	0.324	0.449	0.332	0.511
Galactonate	-0.923	-1.246	-1.416	-1.232	-1.421	0.192
3-phosphoglycerate	0.064	-0.959	-0.969	-1.682	-0.054	-1.090
N-acetylneuraminate	-1.383	-0.243	-0.940	-1.119	-2.863	-0.028

We next analyzed entire pathways and subpathways to interrogate perturbations associated with entire pathways. The most affected superpathway was lipid metabolism (Table 5). Considering all the biochemicals identified and grouped into sub-pathways: long chain fatty acid, phospholipid, acylcarnitine and sphingolipid metabolism were significantly disturbed in GLUT1-DS patients on ketogenic diet (Table 6). The super-pathway analysis confirmed that lipids were the most significantly modified compounds in GLUT1-DS patients on KD (Table 5).

**Table 5:** Super-pathways modified in GLUT1DS patients on ketogenic diet.

SUPER_PATHWAY	Number	p_value
Lipid	305	7.90 e^-06^
Carbohydrate	22	0.023
Amino Acid	147	0.060
Cofactors and Vitamins	17	0.023
Xenobiotics	71	0.099
Nucleotide	28	0.714
Energy	8	0.030
Peptide	34	0.034

**Table 6:** Sub-pathways analysis based on individual metabolites perturbation in 6 GLUT1-DS patients on ketogenic diet.

SUB_PATHWAY	# compounds in subpathway	SUPER PATHWAY	p_value
Long Chain Fatty Acid	13	Lipid	2.15 e^-08^
Phospholipid Metabolism	51	Lipid	3.49 e^-07^
Fatty Acid Metabolism(Acylcarnitine)	15	Lipid	1.34 e^-05^
Sphingolipid Metabolism	23	Lipid	0.0007
Fatty Acid. Monohydroxy	13	Lipid	0.001
Polyunsaturated Fatty Acid (n3 and n6)	14	Lipid	0.0018
Endocannabinoid	5	Lipid	0.009
Polyamine Metabolism	5	Amino Acid	0.011
Lysolipid	48	Lipid	0.015
Steroid	17	Lipid	0.016
Fatty Acid. Branched	2	Lipid	0.019
Fatty Acid. Amide	3	Lipid	0.030
Fatty Acid Metabolism (Acylglycine)	2	Lipid	0.030
Plasmalogen	16	Lipid	0.038
TCA Cycle	7	Energy	0.039
Nicotinate and Nicotinamide Metabolism	5	Cofactors and Vitamins	0.040

#### Carnitine and energy metabolism

**Fig 2:**
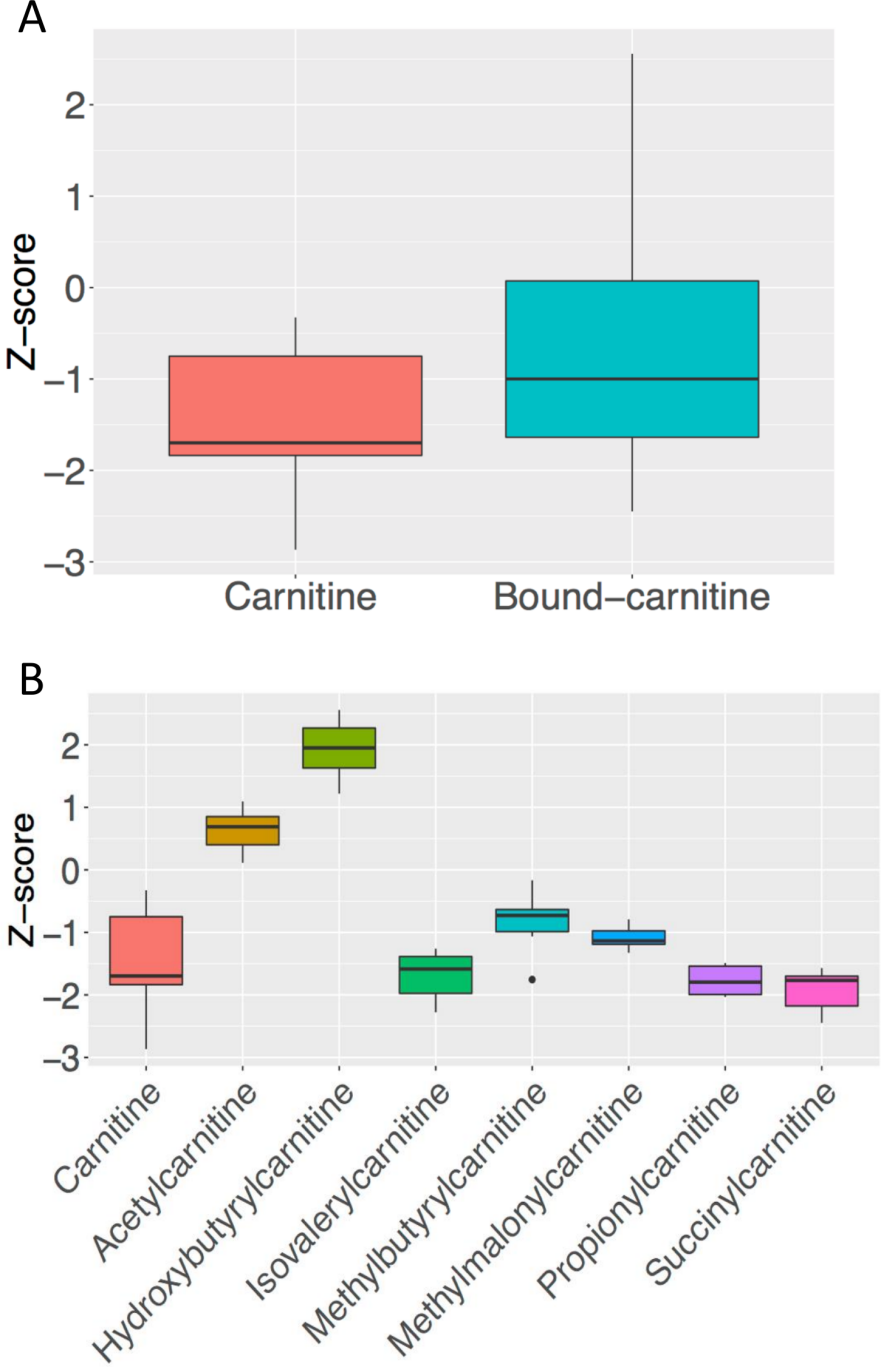
Plasma biochemical profiles of GLUT1-DS patients on KD. A) Low plasma free carnitine in GLUT1-DS patients on ketogenic diet. Box-plot of free and grouped bound-carnitine compounds z-scores for 6 patients with GLUT1-DS on ketogenic diet. Low levels of free carnitine were detected, while all carnitine conjugates showed higher levels in GLUT1-DS patients on ketogenic diet. B) Carnitine-bound metabolites are elevated in plasma of patients on ketogenic diet. Box-plot of free carnitine and specific carnitine-derived compound for 6 patients with GLUT1-DS on ketogenic diet. Higher levels of 3-hydroxybutyrylcarnitine is detected in face of low levels of other carnitine derived compounds (isovalerylcarnitine. 2-methylbutyrylcarnitine. 2-methylmalonyl carnitine. propionylcarnitine. succinylcarnitine) in GLUT1-DS patients on ketogenic diet.

Free carnitine and carnitine conjugates of GLUT1-DS on KD are shown in Table 7. Overall, those acylcarnitines linked to fatty acid metabolism showed higher levels in subjects on KD whereas those acylcarnitines linked to amino acid metabolism showed decreased levels. Specifically, amino acid-related acylcarnitines, 2-methylmalonyl carnitine, isovalerylcarnitine, propionylcarnitine, isobutyrylcarnitine, glutarylcarnitine, carnitine and 2-methylbutyrylcarnitine z-scores for GLUT1-DS patients on KD were significantly lower than for controls. Succinylcarnitine, a molecule representing an anaplerotic entry point into the tricarboxylic acid (TCA) cycle and where the metabolism of several amino acids feeds into the TCA cycle showed decreased levels. Moreover 3-hydroxybutyrylcarnitine, oleoylcarnitine, stearoylcarnitine were significantly higher in patients with GLUT1-DS on KD (Fig 2A and 2B). As expected 3-hydroxybutyrate (BHBA) was significantly (p = 1.86 e^-05^) elevated in plasma with an average z-score of 2.46 (p = 1.86 e^-05^) in individuals on ketogenic diet. Although not all metabolites showed large perturbations, all of them showed consistent directional changes suggesting the overall pathway was affected.

**Table 7:** Significant free carnitine and carnitine derivatives for 6 patients with GLUT1-DS patients.

Biochemical	HMDB	SUPER PATHWAY	SUBPATHWAY	P value	Average z-score
Carnitine	HMDB00062	Lipid	Carnitine Metabolism	0.0100	-1.537
3-hydroxybutyrylcarnitine	HMDB13127	Lipid	Fatty Acid Metabolism	0.0003	1.993
Oleoylcarnitine	HMDB05065	Lipid		0.0009	1.284
Stearoylcarnitine	HMDB00848	Lipid		0.0010	1.387
Palmitoylcarnitine	HMDB00222	Lipid		0.0121	0.930
Myristoylcarnitine	HMDB05066	Lipid		0.0050	0.854
Myristoleoylcarnitine*		Lipid		0.0064	0.536
Laurylcarnitine	HMDB02250	Lipid		0.0002	0.648
Octanoylcarnitine	HMDB00791	Lipid		0.037	0.423
Acetylcarnitine	HMDB00201	Lipid		0.0088	0.696
2-methylbutyrylcarnitine (C5)	HMDB00378	Amino Acid	BCAA Metabolism	0.0194	-0.828
Tiglylcarnitine	HMDB02366	Amino Acid		4.83 e^-05^	-0.347
Isovalerylcarnitine	HMDB00688	Amino Acid		5.76 e^-05^	-1.716
Propionylcarnitine	HMDB00824	Amino Acid		5.81 e^-05^	-1.796
2-methylmalonyl carnitine	HMDB13133	Amino Acid		2.69 e^-14^	-1.111
Isobutyrylcarnitine	HMDB00736	Amino Acid		0.0030	-1.043
Succinylcarnitine		Energy	TCA Cycle	1.51 e^-05^	-1.911
Glutarylcarnitine (C5)	HMDB13130	Amino Acid	Lysine Metabolism	0.0083	-0.886

#### Long and medium-chain fatty acids

8-hydroxyoctanoate, an octanoate derivative, showed significantly lower values in GLUT1-DS patients in both plasma and urine on KD versus controls (p = 1.28 e-12), while other long-chain and medium-chain fatty acids showed significantly (p<0.01) higher values in GLUT1-DS patients versus controls including: erucate (22:1n9), eicosenoate (20:1), nonadecenoate (19:1n9), nonadecanoate (19:0), oleate/vaccenate (18:1), 10-heptadecenoate (17:1n7), margarate (17:0), palmitoleate (16:1n7), palmitate (16:0), myristoleate (14:1n5), myristate (14:0), 5-dodecenoate (12:1n7), and 10-undecenoate (11:1n1) (S2 and S3 Tables).

## Discussion

Reduced brain glucose transport associated with GLUT1 lowered activity or deficiency is the hallmark of GLUT1-DS. Metabolomic profiling, the systematic study of small-molecule products of biochemical pathways has been used to predict diagnosis and monitor different metabolic disorders [1,3,12–15]. We applied an untargeted metabolomics profiling approach on both plasma and CSF samples from GLUT1-DS patients toward unraveling metabolic mechanisms and biomarkers implicated in this disorder.

Currently, the routine diagnostic tools in clinical use for GLUT1-DS are low glucose in CSF and a reduced CSF glucose to blood glucose ratio. Glucose values can vary depending on age [16–18], and screening for glucose levels versus a pediatric-matched population can identify perturbations in metabolites, such as carbohydrate metabolism, to identify signatures of disease (Table 2). The metabolomics approach in three CSF samples of GLUT1-DS not on ketogenic diet patients was applied to achieve a more comprehensive mirror of glucose-dependent pathways in the brain. In the analysis of the initial sample, we identified significantly decreased levels of glucose, fructose, and mannose with elevated levels of inosine and glutamine (Fig 1). The analysis of two subsequent CSF samples revealed decreased levels of most TCA cycle intermediates, elevated isocitrate, and decreased glycerol-3-phosphate (Table 2). The differences in biochemicals detected on the two platforms can be attributed to the different chromatography components of each platform; version 1 contained one LC/MS pos method and version 2 contained two LC/MS pos methods. Increased isocitrate levels in CSF may reflect low enzyme activity of isocitrate dehydrogenase, a NADP^+^-consuming enzyme. Glycerol-3-phosphate has a role in intermediary metabolism in the regulation of glucose lipid and energy metabolism [19–22] and a work in a GLUT1-DS mouse model revealed diminished cerebral lipid synthesis in these mice [23,24]. Isocitrate and glycerol-3-phosphate are known to play roles in cellular energetics and their biochemicals, or a ratio of them given they change in opposite directions, may be biomarkers of disease.

Energy failure has been proved in GLUT1-DS astrocytes [24]. TCA metabolites are globally reduced in GLUT1-DS patients and succinylcarnitine (C4) which arises from the TCA cycle intermediate succinyl-CoA [25] was significantly lower in our cohort (Table 7). Energy impairment is also supported by the recently proved clinical therapeutic efficacy in GLUT1-DS of triheptanoin which is a dietary supplement that replenishes key substrates to the TCA cycle in the brain [26]. We detected 2-aminoheptanoate at significantly higher levels in GLUT1-DS patients (average z-score = 1.4411, p<0.01). Heptanoate, a direct product of triheptanoin metabolism, can easily cross the blood-brain barrier via monocarboxylate transporters and enhance the effect of ketones as fuel to the TCA cycle in the brain [27, 28]. Specifically, 2-aminoheptanoate can be metabolized to two molecules of acetyl-CoA and one molecule of propionyl-CoA. Propionyl-CoA then can be metabolized to succinyl-CoA, an intermediate of the TCA cycle. We speculate that high 2-aminoheptanoate in GLUT1-DS patients might be a compensatory neurochemical mechanism for recovering energy homeostasis induced by ketone bodies.

The KD induced differences in plasma biochemical profiles in lipid, carbohydrate, and amino acid related compounds (Tables 3 and 4). The type of KD can differ in fatty acid content and composition, thereby affecting the levels of free fatty acids in the bloodstream [29]. Within the lipid superfamily, long-chain fatty acids, phospholipids, acylcarnitines, and sphingolipids showed significant perturbations. Medium-chain fatty acids such as octanoic acid and decanoic acid acids have been found to be elevated in the plasma of GLUT1-DS patients on KD [30]. Decanoate showed no changes in our subjects diagnosed with GLUT1-DS, and octanoate showed reduced levels in subjects diagnosed with GLUT1-DS (S2 Table). This profile suggests the KD consumed by these subjects did not contain medium chain triglycerides. Klepper et al. analyzed essential fatty acid levels in the plasma and CSF of 18 GLUT1-deficient patients consuming a ketogenic diet and revealed their respective levels versus control patients could be maintained over the course of several months [31].

Low to normal blood glucose levels in GLUT1-DS have been reported [32]. In our cohort, all GLUT1-DS patients showed significantly lower levels of different carbohydrate compounds; the most significant were those of maltose, sucrose, lactose, glucose, and mannitol/sorbitol (Table 4). Previous reports have suggested that a KD may decrease blood glucose level [33,34].

The brain can utilize ketones as a source of energy in the presence of low levels or absence of glucose. Ketones can be ingested through diet and produced through the metabolism of lipids. Because of this, ketone levels vary according to the dietary regimen, energy balance, physical activities and long-term side effects (e.g. elevated blood lipids, growth retardation) [7]. The neuroprotective effects of ketone bodies rely on alternative brain energy fuel recovery in lipid biosynthesis and influence on neuronal excitability [35,36], although the exact mechanism(s) of action of KD along with biochemical changes that are KD-related are still unknown, and KD is not fully effective in all GLUT1-DS patients [7]. Marin-Valencia et al. showed GLUT1-DS mice had elevated plasma fatty acids and ketone bodies [23]. Although glucose is the main energy source for neurons, cells can also utilize ketones, lactate, pyruvate, glycerol and some amino acids as alternative substrates [37]. Brain amino acids such as glutamate, glutamine, GABA, adenosine, pyruvate and aspartate were preserved in CSF of GLUT1-DS in line with other reports [23]. Specific KD metabolites include BHBA and 3-hydroxybutyrylcarnitine, as reflected in the plasma from 6 patients with GLUT1-DS on KD. Other potential KD-associated metabolites may include: N-acetylglycine, 2-hydroxyisobutyrate, nonadecanoate, alpha-ketobutyrate, 3-hydroxylaurate, 10-nonadecenoate, margarate, 15-methylpalmitate and 2-aminoheptanoate since they showed significant high values in our KD patients. In addition to dietary intervention with a ketogenic diet, plasma from subjects in a fasted state will have elevated ketone bodies and elevated free fatty acids, but the patients in this study were randomly samples and thus, did not have blood taken during a fasted state. Margarate influences the fatty acid network involving nonadecanoate, palmitate and heptadecenoate [38]. 3-hydroxybutyrate and 3-hydroxybutyrylcarnitine can be used as plasma biomarkers for personalized monitoring of individuals and for optimization of their diet.

L-carnitine regulates the flux substrate and energy balance across cell membranes by modulating the transport of long-chain fatty acids into mitochondria [39,40]. Free carnitine levels were significantly lower in GLUT1-DS patients on KD (p = 0.01) (Table 6, Fig 2A and 2B) consistent with those results obtained by other who have examined gradual carnitine depletion with long-term KD treatment [39]. The overall profiles in Table 3 and Table 7 are indicative of the consumption of carnitine and the metabolism of fatty acids through β-oxidation. Ketosis induces chronic formation of acyl-carnitine derivatives causing a dip of free carnitine [39,41,42]. Carnitine deficiency will impair oxidation of fatty acids and ketone production [39]. However, the need for carnitine supplementation in patients on KD remains controversial [39,41]. Based on our results and the low risk associated with L-carnitine supplementation, we suggest oral supplementation with carnitine for GLUT1-DS patients on KD for dietary intervention with a starting dose of 10–50 mg/kg per day [41].

The present study is, to the best of our knowledge, the first to investigate metabolomics perturbation in GLUT1-DS patients. The emerging application of metabolomics in clinical practice could contribute to an informative diagnosis and may provide insights into the pathomechanisms of IEMs, potentially broadening therapeutic choices, as those identified in the analysis of CSF and plasma from subjects diagnosed with GLUT1-DS. Overall, metabolomics has revealed multiple dysregulated metabolites in several pathways in the CSF and plasma of patients diagnosed with GLUT1-DS. From this perspective, metabolomics may provide new insights into the mechanisms regulating GLUT1 function and may open, with the collection of additional data from subjects diagnosed with GLUT1-DS or other glucose transporter deficiencies, new avenues of treatment.

## Supporting information

S1 Table Z-scores of CSF samples of patients 7 (783), 8 (795), and 9. (PDF)

S2 Table Z-scores for plasma samples of patient 1 to patient 6. (PDF)

S3 Table Z-scores for urine sample patient 1, 2, 3, 4, 6 and 8. (PDF)
