# Supplementary material for: Biochemical phenotyping unravels novel metabolic abnormalities and potential biomarkers associated with treatment of GLUT1 deficiency with ketogenic diet
Source: PLoS One. 2017 Sep 29;12(9):e0184022. doi: 10.1371/journal.pone.0184022 (PMC5621665; doi:10.1371/journal.pone.0184022)
Supplement: S1 Table — (PDF) [file pone.0184022.s001.pdf]

Cappuccio et al. Supplemental Table 1

Biochemical	HMDB	Super Pathway	Sub Pathway	ID 783	ID 795	ID 604266
1-(1-enyl-palmitoyl)-2-arachidonoyl-GPC (P-16:0/20:4)*		Lipid	Plasmalogen	0.099066245	-0.36496283	ND
1-(1-enyl-palmitoyl)-2-docosahexaenoyl-GPE (P-16:0/22:6)*		Lipid	Plasmalogen	-0.027552888	-0.351413551	ND
1-(1-enyl-palmitoyl)-2-oleoyl-GPC (P-16:0/18:1)*		Lipid	Plasmalogen	0.914056477	-0.05137141	ND
1-(1-enyl-stearoyl)-2-arachidonoyl-GPE (P-18:0/20:4)*	HMDB05779	Lipid	Plasmalogen	0.255659505	0.538245172	ND
1-(1-enyl-stearoyl)-2-docosahexaenoyl-GPE (P-18:0/22:6)*		Lipid	Plasmalogen	0.107530235	0.095366415	ND
1,2-dioleoyl-GPC (18:1/18:1)*		Lipid	Phospholipid Metabolism	-0.282048321	-0.6291007	ND
1,2-dipalmitoyl-GPC (16:0/16:0)	HMDB00564	Lipid	Phospholipid Metabolism	-0.43617405	-0.680987263	ND
1,5-anhydroglucitol (1,5-AG)	HMDB02712	Carbohydrate	Glycolysis, Gluconeogenesis, and Pyruvate Metabolism	0.25418447	0.542907156	-0.467705
1-arachidonoyl-GPC (20:4n6)*	HMDB10395	Lipid	Lysolipid	-0.424834359	-0.465848159	ND
1-linoleoyl-GPC (18:2)	HMDB10386	Lipid	Lysolipid	0.396716596	-1.599472956	ND
1-methylhistidine	HMDB00001	Amino Acid	Histidine Metabolism	0.878990232	-1.35319819	0.301842161
1-methylimidazoleacetate	HMDB02820	Amino Acid	Histidine Metabolism	-2.145285964	-1.939732253	ND
1-methylnicotinamide	HMDB00699	Cofactors and Vitamins	Nicotinate and Nicotinamide Metabolism	-1.274657357	0.08935488	ND
1-myristoyl-2-palmitoyl-GPC (14:0/16:0)		Lipid	Phospholipid Metabolism	-0.45791284	-0.472511099	ND
1-oleoyl-2-linoleoyl-GPC (18:1/18:2)*		Lipid	Phospholipid Metabolism	-0.592602382	-0.450050447	ND
1-oleoylglycerol (18:1)	HMDB11567	Lipid	Monoacylglycerol	-2.146217711	-2.148309763	ND
1-oleoyl-GPC (18:1)	HMDB02815	Lipid	Lysolipid	-0.80383091	-1.419531093	ND
1-palmitoleoyl-GPC (16:1)*	HMDB10383	Lipid	Lysolipid	-0.391767145	-0.400190395	ND
1-palmitoyl-2-adrenoyl-GPC (16:0/22:4)*		Lipid	Phospholipid Metabolism	-0.269846043	-0.309084143	ND
1-palmitoyl-2-arachidonoyl-GPC (16:0/20:4n6)		Lipid	Phospholipid Metabolism	-0.404277739	-0.434606985	ND
1-palmitoyl-2-dihomo-linolenoyl-GPC (16:0/20:3n3 or 6)*		Lipid	Phospholipid Metabolism	0.109865815	0.099389932	ND
1-palmitoyl-2-docosahexaenoyl-GPC (16:0/22:6)		Lipid	Phospholipid Metabolism	-0.17321315	-0.475519055	ND
1-palmitoyl-2-linoleoyl-GPC (16:0/18:2)		Lipid	Phospholipid Metabolism	-0.505421939	-0.600495495	ND
1-palmitoyl-2-oleoyl-GPC (16:0/18:1)		Lipid	Phospholipid Metabolism	-0.284334233	-0.446368159	ND
1-palmitoyl-2-palmitoleoyl-GPC (16:0/16:1)*		Lipid	Phospholipid Metabolism	-0.630425808	-0.676166147	ND
1-palmitoylglycerol (16:0)	HMDB31074	Lipid	Monoacylglycerol	-0.822779019	-0.601042608	ND
1-palmitoyl-GPC (16:0)	HMDB10382	Lipid	Lysolipid	-0.736163151	-1.035580834	-1.953017557
1-palmityl-2-arachidonoyl-GPC (O-16:0/20:4)*		Lipid	Phospholipid Metabolism	-0.178778575	-0.737356299	ND
1-palmityl-2-oleoyl-GPC (O-16:0/18:1)*		Lipid	Phospholipid Metabolism	0.424850246	-0.057605774	ND
1-stearoyl-2-arachidonoyl-GPC (18:0/20:4)		Lipid	Phospholipid Metabolism	-0.269345802	-0.491192774	ND
1-stearoyl-2-dihomo-linolenoyl-GPC (18:0/20:3n3 or 6)*		Lipid	Phospholipid Metabolism	0.017795132	0.081521293	ND
1-stearoyl-2-docosahexaenoyl-GPC (18:0/22:6)		Lipid	Phospholipid Metabolism	0.109891506	-0.16712524	ND
1-stearoyl-2-docosahexaenoyl-GPE (18:0/22:6)*	HMDB05334	Lipid	Phospholipid Metabolism	0.2568557	0.405392872	ND
1-stearoyl-2-linoleoyl-GPC (18:0/18:2)*		Lipid	Phospholipid Metabolism	-0.397539694	-0.141363325	ND
1-stearoyl-2-oleoyl-GPC (18:0/18:1)		Lipid	Phospholipid Metabolism	-0.035379135	-0.182277218	ND
1-stearoyl-GPC (18:0)	HMDB10384	Lipid	Lysolipid	-0.60859144	-1.105761507	ND
2-aminobutyrate	HMDB00650	Amino Acid	Methionine, Cysteine, SAM and Taurine Metabolism	-0.337353042	-0.493547047	NS
2-aminoheptanoate		Lipid	Fatty Acid, Amino	0.480767071	-1.885157318	-0.338540395
2-aminooctanoate	HMDB00991	Lipid	Fatty Acid, Amino	-1.313934753	-1.308900427	1.329559887
2'-deoxyuridine	HMDB00012	Nucleotide	Pyrimidine Metabolism, Uracil containing	-1.153769476	0.486110008	1.45153472
2-hydroxy-3-methylvalerate	HMDB00317	Amino Acid	Leucine, Isoleucine and Valine Metabolism	0.201716094	-0.231388182	1.015260051
2-hydroxyadipate	HMDB00321	Lipid	Fatty Acid, Dicarboxylate	-0.570819057	0.077078979	ND
2-hydroxybutyrate/2-hydroxyisobutyrate		Amino Acid	Methionine, Cysteine, SAM and Taurine Metabolism	-0.725379483	-2.253410291	-0.19171266
2-methylbutyrylcarnitine (C5)	HMDB00378	Amino Acid	Leucine, Isoleucine and Valine Metabolism	-0.012273154	0.083630293	-1.026727878
2-methylcitrate	HMDB00379	Energy	TCA Cycle	-0.384231292	-0.710028546	1.119860014
2-oxoadipate	HMDB00225	Amino Acid	Lysine Metabolism	-0.114350084	0.090423192	NS
3-(4-hydroxyphenyl)lactate	HMDB00755	Amino Acid	Phenylalanine and Tyrosine Metabolism	-0.772693824	0.416872607	0.44047351
3-carboxy-4-methyl-5-propyl-2-furanpropanoate (CMPF)	HMDB61112	Lipid	Fatty Acid, Dicarboxylate	1.903632925	-0.344483339	ND
3-hydroxy-2-ethylpropionate	HMDB00396	Amino Acid	Leucine, Isoleucine and Valine Metabolism	-0.539057041	-0.504110533	0.503415067
3-hydroxy-3-methylglutarate	HMDB00355	Lipid	MevaloNot Scoredte Metabolism	-0.452356075	-0.124620136	1.73624567
3-hydroxybutyrate (BHBA)	HMDB00357	Lipid	Ketone Bodies	-0.912043932	-1.093402682	NS
3-hydroxyisobutyrate	HMDB00336	Amino Acid	Leucine, Isoleucine and Valine Metabolism	-0.740935187	0.439873122	1.014339463
3-hydroxyoctanoate	HMDB01954	Lipid	Fatty Acid, Monohydroxy	-1.244768323	-1.24807954	1.927743596
3-methoxytyrosine	HMDB01434	Amino Acid	Phenylalanine and Tyrosine Metabolism	-1.086665712	-1.045261201	1.724664191
3-methyl-2-oxobutyrate	HMDB00019	Amino Acid	Leucine, Isoleucine and Valine Metabolism	0.548961205	1.030985606	1.042924443
3-methyl-2-oxovalerate	HMDB03736	Amino Acid	Leucine, Isoleucine and Valine Metabolism	0.046852883	0.109484402	0.760011009
3-methylglutaconate	HMDB00522	Amino Acid	Leucine, Isoleucine and Valine Metabolism	0.119921929	-0.239506418	0.397099751
3-methylglutarylcarnitine (2)	HMDB00552	Amino Acid	Lysine Metabolism	-0.964294858	0.354482849	-0.738092183
3-methylhistidine	HMDB00479	Amino Acid	Histidine Metabolism	1.233907556	-1.450731472	ND
3-ureidopropionate	HMDB00026	Nucleotide	Pyrimidine Metabolism, Uracil containing	0.351302833	0.191405338	0.517854426
4-acetamidobutanoate	HMDB03681	Amino Acid	Polyamine Metabolism	-0.239484898	-0.040615479	1.840151875
4-guanidinobutanoate	HMDB03464	Amino Acid	Guanidino and Acetamido Metabolism	-1.959732872	-2.181070589	-0.639199913
4-methyl-2-oxopentanoate	HMDB00695	Amino Acid	Leucine, Isoleucine and Valine Metabolism	0.275848845	0.519912011	1.240314168
5,6-dihydrothymine	HMDB00079	Nucleotide	Pyrimidine Metabolism, Thymine containing	-0.485903913	-0.605567283	0.786393483
5-hydroxyhexanoate	HMDB00525	Lipid	Fatty Acid, Monohydroxy	-0.714775001	-0.712795655	2.234327483
5-methyltetrahydrofolate (5MeTHF)	HMDB01396	Cofactors and Vitamins	Folate Metabolism	0.194443292	-0.561902706	NS
5-methylthioadenosine (MTA)	HMDB01173	Amino Acid	Polyamine Metabolism	0.199066042	0.34070651	NS
5-methyluridine (ribothymidine)	HMDB00884	Nucleotide	Pyrimidine Metabolism, Uracil containing	0.247229729	0.030248525	1.718897694
5-oxoproline	HMDB00267	Amino Acid	Glutathione Metabolism	0.080240445	-1.029972971	-1.347044156
6-oxopiperidine-2-carboxylate		Amino Acid	Lysine Metabolism	-0.181729168	-0.973879144	0.251267851
7-alpha-hydroxy-3-oxo-4-cholestenoate (7-Hoca)	HMDB12458	Lipid	Sterol	0.325756973	-0.964685325	1.440668076
7-methylguanine	HMDB00897	Nucleotide	Purine Metabolism, Guanine containing	-0.551539941	-1.166984951	2.00941539
7-methylxanthine	HMDB01991	Xenobiotics	Xanthine Metabolism	2.902670036	0.707966391	ND
acetylcarnitine	HMDB00201	Lipid	Fatty Acid Metabolism(Acyl Carnitine)	0.46511319	-0.0348918	1.729691732
acisoga		Amino Acid	Polyamine Metabolism	1.06889301	-1.293897846	0.318464871
aconitate [cis or trans]	HMDB00072	Energy	TCA Cycle	-0.47036224	-0.187123234	ND
adenine	HMDB00034	Nucleotide	Purine Metabolism, Adenine containing	-0.094362177	-1.107718306	-0.754542588
adenosine	HMDB00050	Nucleotide	Purine Metabolism, Adenine containing	-0.559096654	-1.634373795	NS
alanine	HMDB00161	Amino Acid	Alanine and Aspartate Metabolism	-0.385538823	0.215908698	0.882472551
allantoin	HMDB00462	Nucleotide	Purine Metabolism, (Hypo)Xanthine/Inosine containing	-0.257042502	-0.482753018	0.870215782
alpha-hydroxyisocaproate	HMDB00746	Amino Acid	Leucine, Isoleucine and Valine Metabolism	0.783337396	1.001161698	0.999670503
alpha-hydroxyisovalerate	HMDB00407	Amino Acid	Leucine, Isoleucine and Valine Metabolism	0.331836121	0.264125426	-0.866837303
alpha-ketoglutarate	HMDB00208	Energy	TCA Cycle	-0.748510373	-0.200697999	1.148236887
arabitol/xylitol		Carbohydrate	Pentose Metabolism	-0.197734985	-0.595146443	1.40664624
arabonate/xylonate		Carbohydrate	Pentose Metabolism	-0.515469372	-1.244528055	1.11004356
arginine	HMDB00517	Amino Acid	Urea cycle; Arginine and Proline Metabolism	1.117410988	0.2864163	-0.298422772
argininosuccinate	HMDB00052	Amino Acid	Urea cycle; Arginine and Proline Metabolism	-0.108256819	-0.22291222	ND
asparagine	HMDB00168	Amino Acid	Alanine and Aspartate Metabolism	1.089535114	0.841239379	1.674633339
aspartate	HMDB00191	Amino Acid	Alanine and Aspartate Metabolism	-0.55171047	-1.042796533	ND

Cappuccio et al. Supplemental Table 1

Biochemical	HMDB	Super Pathway	Sub Pathway	ID 783	ID 795	ID 604266
beta-hydroxyisovalerate	HMDB00754	Amino Acid	Leucine, Isoleucine and Valine Metabolism	-0.146089767	-0.060879856	0.712102331
betaine	HMDB00043	Amino Acid	Glycine, Serine and Threonine Metabolism	-0.842808808	-1.67059211	1.476474254
bilirubin (E,E)*		Cofactors and Vitamins	Hemoglobin and Porphyrin Metabolism	-0.474662016	-0.48202175	NS
bilirubin (Z,Z)	HMDB00054	Cofactors and Vitamins	Hemoglobin and Porphyrin Metabolism	-0.189701746	-0.482315197	ND
butyrylcarnitine	HMDB02013	Lipid	Fatty Acid Metabolism (also BCAA Metabolism)	0.315341504	-0.304718324	1.087628831
caffeine	HMDB01847	Xenobiotics	Xanthine Metabolism	1.198584083	0.788253684	ND
carboxyethyl-GABA	HMDB02201	Amino Acid	Glutamate Metabolism	0.124131616	-0.044555768	ND
carnitine	HMDB00062	Lipid	Carnitine Metabolism	1.272159342	0.214464648	-0.250097318
catechol sulfate	HMDB59724	Xenobiotics	Benzoate Metabolism	2.187250416	-0.394810217	ND
C-glycosyltryptophan		Amino Acid	Tryptophan Metabolism	-0.112200143	-0.261409376	NS
choline	HMDB00097	Lipid	Phospholipid Metabolism	-0.934474727	0.264106894	1.318342575
choline phosphate	HMDB01565	Lipid	Phospholipid Metabolism	-0.299723698	-0.252990546	ND
cis-4-decenoyl carnitine		Lipid	Fatty Acid Metabolism(Acyl Carnitine)	-1.458107987	-0.624173618	ND
citramalate	HMDB00426	Amino Acid	Glutamate Metabolism	-2.150552841	-1.335819104	ND
citrate	HMDB00094	Energy	TCA Cycle	0.353540359	0.189774292	0.141542309
citrulline	HMDB00904	Amino Acid	Urea cycle; Arginine and Proline Metabolism	0.896857254	1.364895857	1.265111976
creatine	HMDB00064	Amino Acid	Creatine Metabolism	1.073969883	2.172137989	3.53592466
creatine phosphate	HMDB01511	Amino Acid	Creatine Metabolism	0.845691384	0.969188968	ND
creatinine	HMDB00562	Amino Acid	Creatine Metabolism	0.569930909	-0.18786005	-1.163235184
cyclo(ala-pro)		Peptide	Dipeptide	0.741957895	-0.07089178	1.702858329
cyclo(pro-val)		Peptide	Dipeptide	2.677670881	1.712890737	ND
cys-gly, oxidized		Amino Acid	Glutathione Metabolism	0.460468575	0.327839431	NS
cystathionine	HMDB00099	Amino Acid	Methionine, Cysteine, SAM and Taurine Metabolism	-1.686687894	-1.686389126	ND
cysteine	HMDB00574	Amino Acid	Methionine, Cysteine, SAM and Taurine Metabolism	-1.143570571	-0.515803998	ND
cytidine	HMDB00089	Nucleotide	Pyrimidine Metabolism, Cytidine containing	-0.232155199	0.402371852	1.398614119
deoxycarnitine	HMDB01161	Lipid	Carnitine Metabolism	0.691524699	1.149730775	1.940566836
dimethyl sulfone	HMDB04983	Xenobiotics	Chemical	3.351693256	-2.067646753	0.113310405
dimethylarginine (SDMA + ADMA)	HMDB01539	Amino Acid	Urea cycle; Arginine and Proline Metabolism	-0.592846866	-0.688141496	3.102270459
dimethylglycine	HMDB00092	Amino Acid	Glycine, Serine and Threonine Metabolism	0.492914051	0.100200409	ND
dimethylmalonic acid	HMDB02001	Lipid	Fatty Acid, Dicarboxylate	-0.129882541	-1.35983422	NS
erythritol	HMDB02994	Xenobiotics	Food Component/Plant	-0.553364108	-1.409056383	0.61989043
erythronate*	HMDB00613	Carbohydrate	Aminosugar Metabolism	-0.113801304	-0.707753876	0.905041571
ethylmalonate	HMDB00622	Amino Acid	Leucine, Isoleucine and Valine Metabolism	-0.401623019	0.508434957	2.045606546
fructose	HMDB00660	Carbohydrate	Fructose, Mannose and Galactose Metabolism	-0.028516023	-1.119513811	-2.80349644
fumarate	HMDB00134	Energy	TCA Cycle	-0.897430407	-0.901102149	0.25888257
galactitol (dulcitol)	HMDB00107	Carbohydrate	Fructose, Mannose and Galactose Metabolism	-0.409142202	-0.606058824	ND
gamma-glutamyl-epsilon-lysine	HMDB03869	Peptide	Gamma-glutamyl Amino Acid	-0.779403114	-0.607461	ND
gamma-glutamylglutamine	HMDB11738	Peptide	Gamma-glutamyl Amino Acid	-0.394628206	0.80671082	2.809940474
gamma-glutamylhistidine		Peptide	Gamma-glutamyl Amino Acid	-1.513301201	0.056306705	ND
gamma-glutamylleucine	HMDB11171	Peptide	Gamma-glutamyl Amino Acid	-1.401213916	-1.409284015	NS
gamma-glutamylthreonine	HMDB29159	Peptide	Gamma-glutamyl Amino Acid	0.522731965	-0.176107415	2.055720327
gamma-glutamylvaline	HMDB11172	Peptide	Gamma-glutamyl Amino Acid	0.529711188	0.980978177	NS
gamma-tocopherol/beta-tocopherol		Cofactors and Vitamins	Tocopherol Metabolism	-0.429925064	-0.433960981	ND
gluconate	HMDB00625	Xenobiotics	Food Component/Plant	-0.664969286	-0.533961104	0.714384588
glucose	HMDB00122	Carbohydrate	Glycolysis, Gluconeogenesis, and Pyruvate Metabolism	-0.698262003	-0.609203352	-3.197722042
glucuronate	HMDB00127	Carbohydrate	Aminosugar Metabolism	-0.832882895	-0.832542939	1.803434169
glutamate	HMDB00148	Amino Acid	Glutamate Metabolism	-0.637136067	-0.684108776	-0.504300159
glutamate, gamma-methyl ester		Amino Acid	Glutamate Metabolism	0.454058276	0.27945658	ND
glutamine	HMDB00641	Amino Acid	Glutamate Metabolism	0.580314951	-0.298076999	3.980567952
glutarate (pentanedioate)	HMDB00661	Amino Acid	Lysine Metabolism	-1.459769382	-0.505894233	ND
glutarylcarnitine (C5)	HMDB13130	Amino Acid	Lysine Metabolism	0.178115833	-0.561583352	1.813019868
glycerate	HMDB00139	Carbohydrate	Glycolysis, Gluconeogenesis, and Pyruvate Metabolism	-1.815653506	-2.309943729	-0.596899944
glycerol	HMDB00131	Lipid	Glycerolipid Metabolism	-1.096224356	-0.740059495	0.008934811
glycerol 3-phosphate	HMDB00126	Lipid	Glycerolipid Metabolism	-3.694002972	-2.341289323	ND
glycerophosphoethanolamine	HMDB00114	Lipid	Phospholipid Metabolism	-0.928307957	-0.491286866	ND
glycerophosphoinositol*		Lipid	Phospholipid Metabolism	0.167193272	0.379042949	ND
glycine	HMDB00123	Amino Acid	Glycine, Serine and Threonine Metabolism	-1.276486958	1.057879605	NS
glycylproline	HMDB00721	Peptide	Dipeptide	-1.342910643	-1.35715925	3.078073521
guanidinoacetate	HMDB00128	Amino Acid	Creatine Metabolism	-1.200486464	-0.463743185	ND
guanosine	HMDB00133	Nucleotide	Purine Metabolism, Guanine containing	-2.481487231	-0.722075728	NS
gulonate*	HMDB03290	Cofactors and Vitamins	Ascorbate and Aldarate Metabolism	-0.163485572	-0.800759552	1.618521351
heme	HMDB03178	Cofactors and Vitamins	Hemoglobin and Porphyrin Metabolism	-0.634755006	0.246104543	ND
hexanoylcarnitine	HMDB00705	Lipid	Fatty Acid Metabolism(Acyl Carnitine)	-0.999732151	-0.999176629	0.436661458
hippurate	HMDB00714	Xenobiotics	Benzoate Metabolism	0.486897127	-0.686266979	-0.337061218
histidine	HMDB00177	Amino Acid	Histidine Metabolism	-0.434113093	-0.522453536	-0.540749029
homoarginine	HMDB00670	Amino Acid	Urea cycle; Arginine and Proline Metabolism	0.179129941	1.408577743	ND
homocarnosine	HMDB00745	Peptide	Dipeptide Derivative	-0.989866864	-0.201683867	NS
homovanillate (HVA)	HMDB00118	Amino Acid	Phenylalanine and Tyrosine Metabolism	-0.833427263	-0.180307215	0.667388744
hypoxanthine	HMDB00157	Nucleotide	Purine Metabolism, (Hypo)Xanthine/Inosine containing	-0.507347762	-1.27234839	0.697486173
indoleacetate	HMDB00197	Amino Acid	Tryptophan Metabolism	-1.353557845	-1.50281382	1.640561179
indolelactate	HMDB00671	Amino Acid	Tryptophan Metabolism	-0.511596053	-0.524768771	0.946186291
inosine	HMDB00195	Nucleotide	Purine Metabolism, (Hypo)Xanthine/Inosine containing	0.001203624	0.287114764	3.908112136
isobutyrylcarnitine	HMDB00736	Amino Acid	Leucine, Isoleucine and Valine Metabolism	0.55532967	1.284151638	-0.162174628
isocitrate	HMDB00193	Energy	TCA Cycle	1.846227245	2.204689175	ND
isoleucine	HMDB00172	Amino Acid	Leucine, Isoleucine and Valine Metabolism	0.752047702	0.546500816	0.337794666
isovalerylcarnitine	HMDB00688	Amino Acid	Leucine, Isoleucine and Valine Metabolism	-0.464559256	0.581979886	0.573053412
kynurenine	HMDB00684	Amino Acid	Tryptophan Metabolism	-0.210974134	-1.814124324	1.509513483
kynurenate	HMDB00715	Amino Acid	Tryptophan Metabolism	-1.055482033	-1.05464405	1.590556886
lactate	HMDB00190	Carbohydrate	Glycolysis, Gluconeogenesis, and Pyruvate Metabolism	-1.286308761	-0.964085537	-1.076604942
leucine	HMDB00687	Amino Acid	Leucine, Isoleucine and Valine Metabolism	1.107159241	0.831701704	1.670663668
lidocaine	HMDB14426	Xenobiotics	Drug	-1.297212555	-1.296214791	ND
lysine	HMDB00182	Amino Acid	Lysine Metabolism	0.584549055	1.146292676	0.322209966
malate	HMDB00156	Energy	TCA Cycle	-1.77169358	-0.805111213	0.186784574
maleate	HMDB00176	Lipid	Fatty Acid, Dicarboxylate	-0.112485389	-0.536072434	0.500360989
malonate	HMDB00691	Lipid	Fatty Acid Synthesis	0.478215967	0.452623484	NS
mannitol/sorbitol	HMDB00247	Carbohydrate	Fructose, Mannose and Galactose Metabolism	-0.536443091	-1.056814724	0.64808382
mannose	HMDB00169	Carbohydrate	Fructose, Mannose and Galactose Metabolism	0.152375358	0.012701655	-2.856890138
methionine	HMDB00696	Amino Acid	Methionine, Cysteine, SAM and Taurine Metabolism	0.377271137	0.392925878	0.370571711
methionine sulfone		Amino Acid	Methionine, Cysteine, SAM and Taurine Metabolism	-2.384528061	-0.248508743	NS
methionine sulfoxide	HMDB02005	Amino Acid	Methionine, Cysteine, SAM and Taurine Metabolism	-1.293382528	-0.683423028	ND
methyl-4-hydroxybenzoate sulfate		Xenobiotics	Benzoate Metabolism	-0.828616983	-0.831195436	NS
methylmalonate (MMA)	HMDB00202	Lipid	Fatty Acid Metabolism (also BCAA Metabolism)	-0.024464131	1.321892891	ND

Cappuccio et al. Supplemental Table 1

Biochemical	HMDB	Super Pathway	Sub Pathway	ID 783	ID 795	ID 604266
methylsuccinate	HMDB01844	Amino Acid	Leucine, Isoleucine and Valine Metabolism	-1.179350234	-1.177167687	NS
methylsuccinoylcarnitine (1)		Amino Acid	Leucine, Isoleucine and Valine Metabolism	0.923054896	0.462102227	ND
myo-inositol	HMDB00211	Lipid	Inositol Metabolism	-0.693325803	-0.249455185	1.87255573
N1-Methyl-2-pyridone-5-carboxamide	HMDB04193	Cofactors and Vitamins	Nicotinate and Nicotinamide Metabolism	-0.369634812	-1.252142385	0.293430603
N1-methyladenosine	HMDB03331	Nucleotide	Purine Metabolism, Adenine containing	-1.512089307	-1.420607224	ND
N2,N2-dimethylguanosine	HMDB04824	Nucleotide	Purine Metabolism, Guanine containing	-0.785972596	-1.52222809	1.586896364
N2-acetyllysine/N6-acetyllysine		Amino Acid	Lysine Metabolism	0.233522291	-1.377501022	1.190885577
N2-methylguanosine	HMDB05862	Nucleotide	Purine Metabolism, Guanine containing	0.250635143	-0.453337662	2.974817794
N6,N6,N6-trimethyllysine	HMDB01325	Amino Acid	Lysine Metabolism	0.023922065	-0.477948695	1.932147312
N6-succinyladenosine	HMDB00912	Nucleotide	Purine Metabolism, Adenine containing	1.03195197	0.795921388	0.874149926
N-acetyl-3-methylhistidine*		Amino Acid	Histidine Metabolism	0.057806273	-0.376351942	NS
N-acetylalanine	HMDB00766	Amino Acid	Alanine and Aspartate Metabolism	-0.915877731	-0.408337259	2.393064364
N-acetylasparagine	HMDB06028	Amino Acid	Alanine and Aspartate Metabolism	-1.681295952	-1.054011814	1.985291315
N-acetylaspartate (NAA)	HMDB00812	Amino Acid	Alanine and Aspartate Metabolism	-1.713810352	-0.536485566	0.704846559
N-acetyl-aspartyl-glutamate (NAAG)	HMDB01067	Amino Acid	Glutamate Metabolism	-0.086372358	0.233996576	2.457414942
N-acetyl-beta-alanine		Nucleotide	Pyrimidine Metabolism, Uracil containing	-1.329353004	0.160240201	0.20958666
N-acetylglutamate	HMDB01138	Amino Acid	Glutamate Metabolism	-0.953528962	-0.083478081	NS
N-acetylglutamine	HMDB06029	Amino Acid	Glutamate Metabolism	-1.177946778	0.112778919	2.009611256
N-acetylglycine	HMDB00532	Amino Acid	Glycine, Serine and Threonine Metabolism	-1.205310774	-0.5329784	-0.264443897
N-acetylhistidine	HMDB32055	Amino Acid	Histidine Metabolism	-0.948339854	-0.424693734	2.793316077
N-acetylisoleucine		Amino Acid	Leucine, Isoleucine and Valine Metabolism	0.203813564	-0.035048103	1.193379837
N-acetylmethionine	HMDB11745	Amino Acid	Methionine, Cysteine, SAM and Taurine Metabolism	-1.298330874	-0.357498111	1.288330556
N-acetylneuraminate	HMDB00230	Carbohydrate	Aminosugar Metabolism	-0.146141428	-0.199537677	2.09264
N-acetylphenylalanine	HMDB00512	Amino Acid	Phenylalanine and Tyrosine Metabolism	-0.067890676	-1.258699581	NS
N-acetylputrescine	HMDB02064	Amino Acid	Polyamine Metabolism	-0.980691445	-0.919051442	3.803726394
N-acetylserine	HMDB02931	Amino Acid	Glycine, Serine and Threonine Metabolism	-0.352527768	-0.071764217	1.977047237
N-acetyltaurine		Amino Acid	Methionine, Cysteine, SAM and Taurine Metabolism	-0.843320424	-0.735609051	-0.163679332
N-acetylthreonine		Amino Acid	Glycine, Serine and Threonine Metabolism	-0.367641826	-0.619608588	-0.034236757
N-acetylvaline	HMDB11757	Amino Acid	Leucine, Isoleucine and Valine Metabolism	-0.785760378	-0.301860803	1.88077349
N-delta-acetylornithine		Amino Acid	Urea cycle; Arginine and Proline Metabolism	-0.058261248	1.892291643	1.745556206
N-formylmethionine	HMDB01015	Amino Acid	Methionine, Cysteine, SAM and Taurine Metabolism	-1.070999364	0.265599413	0.887801866
nicotinamide	HMDB01406	Cofactors and Vitamins	Nicotinate and Nicotinamide Metabolism	-1.517632271	-0.355486242	0.165322272
nicotinamide riboside	HMDB00855	Cofactors and Vitamins	Nicotinate and Nicotinamide Metabolism	0.249393234	0.374272158	ND
N-methylproline		Amino Acid	Urea cycle; Arginine and Proline Metabolism	1.822837331	2.020898619	NS
octanoylcarnitine	HMDB00791	Lipid	Fatty Acid Metabolism(Acyl Carnitine)	-0.288652873	0.229966332	1.425259133
oleamide	HMDB02117	Lipid	Fatty Acid, Amide	-0.172540284	-0.553452799	ND
ornithine	HMDB03374	Amino Acid	Urea cycle; Arginine and Proline Metabolism	-0.001648743	0.114472251	NS
orotate	HMDB00226	Nucleotide	Pyrimidine Metabolism, Orotate containing	-0.92607554	-0.438346938	1.615358492
orotidine	HMDB00788	Nucleotide	Pyrimidine Metabolism, Orotate containing	0.237657844	-0.011855086	1.526495554
O-sulfo-L-tyrosine		Xenobiotics	Chemical	0.122727093	-0.232223605	2.094395259
oxalate (ethanedioate)	HMDB02329	Cofactors and Vitamins	Ascorbate and Aldarate Metabolism	-1.66399836	-1.465257825	NS
palmitic amide		Lipid	Fatty Acid, Amide	-0.440851686	-0.569521964	ND
palmitoyl sphingomyelin (d18:1/16:0)		Lipid	Sphingolipid Metabolism	-0.283511776	-0.434660388	ND
pantothenate	HMDB00210	Cofactors and Vitamins	Pantothenate and CoA Metabolism	-0.047959943	-0.418863384	1.558709974
p-cresol sulfate	HMDB11635	Amino Acid	Phenylalanine and Tyrosine Metabolism	0.951561674	-0.86739518	-2.319029629
phenol sulfate	HMDB60015	Amino Acid	Phenylalanine and Tyrosine Metabolism	-0.791432045	-0.473784666	-1.322280297
phenylacetylglutamine	HMDB06344	Peptide	Acetylated Peptides	0.213437077	-1.659992004	-2.023524986
phenylalanine	HMDB00159	Amino Acid	Phenylalanine and Tyrosine Metabolism	0.828757662	0.171275052	0.580045199
phenyllactate (PLA)	HMDB00779	Amino Acid	Phenylalanine and Tyrosine Metabolism	-1.545514774	-1.55132341	0.742828864
phosphate	HMDB01429	Energy	Oxidative Phosphorylation	0.587342302	0.353421437	-0.446153527
picolinate	HMDB02243	Amino Acid	Tryptophan Metabolism	-0.653624182	-0.724429758	ND
pipecolate	HMDB00070	Amino Acid	Lysine Metabolism	0.044722599	0.093113151	-0.469338238
pro-hydroxy-pro	HMDB06695	Amino Acid	Urea cycle; Arginine and Proline Metabolism	1.032333875	-0.214835779	2.622669056
proline	HMDB00162	Amino Acid	Urea cycle; Arginine and Proline Metabolism	-0.665274753	-0.554266664	1.900101167
propionylcarnitine	HMDB00824	Lipid	Fatty Acid Metabolism (also BCAA Metabolism)	0.132522968	0.608436354	1.124314789
pseudouridine	HMDB00767	Nucleotide	Pyrimidine Metabolism, Uracil containing	0.694451879	-0.076553247	2.136269624
pyridoxal	HMDB01545	Cofactors and Vitamins	Vitamin B6 Metabolism	-0.958083847	-1.063909544	0.493924852
pyridoxate	HMDB00017	Cofactors and Vitamins	Vitamin B6 Metabolism	-0.943102888	-0.897181587	0.110672948
pyroglutamine*		Amino Acid	Glutamate Metabolism	-1.042746878	0.043487342	0.067036559
pyroglutamylglutamine		Peptide	Dipeptide	-1.3147238	-1.320791584	0.445841262
pyruvate	HMDB00243	Carbohydrate	Glycolysis, Gluconeogenesis, and Pyruvate Metabolism	-0.03358942	0.491015336	NS
quinate	HMDB03072	Xenobiotics	Food Component/Plant	0.74481935	0.657124844	ND
retinol (Vitamin A)	HMDB00305	Cofactors and Vitamins	Vitamin A Metabolism	-0.931665076	-0.928907455	ND
ribitol	HMDB00508	Carbohydrate	Pentose Metabolism	-0.383286546	-0.395832373	1.873541923
ribonate	HMDB00867	Carbohydrate	Pentose Metabolism	-0.175511063	-0.993123309	0.608069633
ribose	HMDB00283	Carbohydrate	Pentose Metabolism	0.256891206	0.926935404	ND
S-adenosylhomocysteine (SAH)	HMDB00939	Amino Acid	Methionine, Cysteine, SAM and Taurine Metabolism	-0.738715847	-0.7273768	0.815039155
salicylate	HMDB01895	Xenobiotics	Drug	1.007423935	1.862034135	ND
serine	HMDB00187	Amino Acid	Glycine, Serine and Threonine Metabolism	0.153565268	-0.277758927	1.385930906
S-methylcysteine	HMDB02108	Amino Acid	Methionine, Cysteine, SAM and Taurine Metabolism	-1.927709249	-0.876080486	0.732225256
spermidine	HMDB01257	Amino Acid	Polyamine Metabolism	-0.150618072	0.291948482	2.180627029
sphingomyelin (d18:1/14:0, d16:1/16:0)*		Lipid	Sphingolipid Metabolism	-0.258379804	-0.023575201	ND
sphingomyelin (d18:1/18:1, d18:2/18:0)		Lipid	Sphingolipid Metabolism	0.421440565	0.40836177	ND
sphingomyelin (d18:1/20:0, d16:1/22:0)*		Lipid	Sphingolipid Metabolism	0.621714854	0.198327492	ND
sphingomyelin (d18:1/24:1, d18:2/24:0)*		Lipid	Sphingolipid Metabolism	-0.06188275	0.074440783	ND
sphingomyelin (d18:2/16:0, d18:1/16:1)*		Lipid	Sphingolipid Metabolism	0.004528325	-1.377309756	ND
sphingosine	HMDB00252	Lipid	Sphingolipid Metabolism	0.989219359	-0.111028807	ND
stachydrine	HMDB04827	Xenobiotics	Food Component/Plant	1.650805361	1.490610743	0.230144434
stearoyl sphingomyelin (d18:1/18:0)	HMDB01348	Lipid	Sphingolipid Metabolism	-0.017938063	-0.144991409	ND
succinimide		Xenobiotics	Chemical	-1.108277156	-1.111245567	ND
succinate	HMDB00254	Energy	TCA Cycle	-0.62814289	-0.566064216	-0.360006941
succinylcarnitine		Energy	TCA Cycle	-0.213517639	-0.51489908	1.378407868
sucrose	HMDB00258	Carbohydrate	Disaccharides and Oligosaccharides	0.4998844	-1.309445464	ND
sulfate*	HMDB01448	Xenobiotics	Chemical	-0.310308247	-0.384509273	1.208430566
tartarate	HMDB00956	Xenobiotics	Food Component/Plant	-1.930123251	-1.925525064	ND
tartronate (hydroxymalonate)	HMDB35227	Xenobiotics	Bacterial/Fungal	-1.911844228	-2.423875435	-0.65951911
taurine	HMDB00251	Amino Acid	Methionine, Cysteine, SAM and Taurine Metabolism	0.54776527	0.411506628	0.945735345
theobromine	HMDB02825	Xenobiotics	Xanthine Metabolism	1.827803704	0.812509898	0.437773358
theophylline	HMDB01889	Xenobiotics	Xanthine Metabolism	1.710191771	0.882517447	1.117473862
threonine	HMDB00167	Amino Acid	Glycine, Serine and Threonine Metabolism	0.688705769	0.000932945	1.295430582
threonate	HMDB00943	Cofactors and Vitamins	Ascorbate and Aldarate Metabolism	-1.779935578	-1.477405394	0.664014553
tiglylcarnitine	HMDB02366	Amino Acid	Leucine, Isoleucine and Valine Metabolism	-0.461479322	0.99209944	0.63457934

Cappuccio et al. Supplemental Table 1

Biochemical	HMDB	Super Pathway	Sub Pathway	ID 783	ID 795	ID 604266
trans-4-hydroxyproline	HMDB00725	Amino Acid	Urea cycle; Arginine and Proline Metabolism	0.071443286	-0.850173069	2.34651815
trigonelline (N'-methylnicotinate)	HMDB00875	Cofactors and Vitamins	Nicotinate and Nicotinamide Metabolism	1.193924928	0.691082747	ND
trimethylamine N-oxide	HMDB00925	Lipid	Phospholipid Metabolism	0.758770305	-0.878851016	ND
trizma acetate		Xenobiotics	Chemical	-1.170730381	0.217238059	ND
tryptophan	HMDB00929	Amino Acid	Tryptophan Metabolism	-0.116825047	0.415542703	0.953498922
tryptophan betaine	HMDB61115	Amino Acid	Tryptophan Metabolism	1.079807093	0.806052256	1.613247274
tyrosine	HMDB00158	Amino Acid	Phenylalanine and Tyrosine Metabolism	1.149064659	1.47821603	0.419036688
uracil	HMDB00300	Nucleotide	Pyrimidine Metabolism, Uracil containing	0.578445561	0.654104456	1.031275071
urate	HMDB00289	Nucleotide	Purine Metabolism, (Hypo)Xanthine/Inosine containing	0.752637986	-0.344027092	0.120163095
urea	HMDB00294	Amino Acid	Urea cycle; Arginine and Proline Metabolism	-0.129004359	0.554257926	-0.185523141
uridine	HMDB00296	Nucleotide	Pyrimidine Metabolism, Uracil containing	0.191569108	0.894324959	0.160595168
valine	HMDB00883	Amino Acid	Leucine, Isoleucine and Valine Metabolism	1.021115233	0.849699996	1.138961759
xanthine	HMDB00292	Nucleotide	Purine Metabolism, (Hypo)Xanthine/Inosine containing	-0.504594881	-0.509555023	1.470480583
