# Supplementary material for: Biochemical phenotyping unravels novel metabolic abnormalities and potential biomarkers associated with treatment of GLUT1 deficiency with ketogenic diet
Source: PLoS One. 2017 Sep 29;12(9):e0184022. doi: 10.1371/journal.pone.0184022 (PMC5621665; doi:10.1371/journal.pone.0184022)
Supplement: S2 Table — (PDF) [file pone.0184022.s002.pdf]

Supplemental Table 2. Complete list of Z-scores from plasma samples of patients 1 to 6 on ketogenic diet.

CHEMICAL_NAME	HMDB	SUPER_PATHWAY	SUB_PATHWAY	ID-796	ID-784	ID-786	ID-791	ID-788	ID-789
1,2-dilinoleoyl-GPC (18:2/18:2)		Lipid	Phospholipid Metabolism	-0,322356362	-0,194459758	0,484096879	0,22677983	-1,517756517	-0,659939017
1,2-dipalmitoyl-GPC (16:0/16:0)	HMDB00564	Lipid	Phospholipid Metabolism	-0,738517403	-1,796311805	-0,538913369	-0,737054572	-0,078732448	-0,215398901
1,5-anhydroglucitol (1,5-AG)	HMDB02712	Carbohydrate	Glycolysis, Gluconeogenesis, and Pyruvate Metabolism	-0,93240674	-0,503850393	-1,397483554	-1,37649698	-1,221826708	-0,756434976
1-(1-enyl-oleoyl)-GPE (P-18:1)*		Lipid	Lysoplasmalogen	-0,625284738	0,002193851	0,413269099	-0,689975599	-0,142341846	0,623461859
1-(1-enyl-palmitoyl)-2-arachidonoyl-GPC (P-16:0/20:4)*		Lipid	Plasmalogen	-0,896732532	-0,225495186	-0,339764286	-1,331453384	0,572576262	0,610452168
1-(1-enyl-palmitoyl)-2-docosahexaenoyl-GPC (P-16:0/22:6)*		Lipid	Plasmalogen	-1,527707126	-0,338790699	-0,369494429	-0,997232681	-0,467165498	0,541700838
1-(1-enyl-palmitoyl)-2-linoleoyl-GPC (P-16:0/18:2)*		Lipid	Plasmalogen	-0,113342573	-0,138043108	1,422436383	-0,026295849	0,902163997	0,224067603
1-(1-enyl-palmitoyl)-2-myristoyl-GPC (P-16:0/14:0)*		Lipid	Plasmalogen	-0,056726575	-0,695617289	0,50159706	-0,403970582	0,505243239	0,727641748
1-(1-enyl-palmitoyl)-2-palmitoyl-GPC (P-16:0/16:0)*		Lipid	Plasmalogen	-0,767833204	0,079797274	0,359314437	-0,606551958	0,255631128	0,459516958
1-(1-enyl-palmitoyl)-GPC (P-16:0)*		Lipid	Lysoplasmalogen	0,45467235	-0,358119987	1,542754655	-0,684940771	1,060825928	1,0505562
1-(1-enyl-palmitoyl)-GPE (P-16:0)*		Lipid	Lysoplasmalogen	-0,6597367	-0,359480886	0,255536031	-0,748901842	-0,409898527	0,577171428
1-(1-enyl-stearoyl)-2-arachidonoyl-GPE (P-18:0/20:4)*	HMDB05779	Lipid	Plasmalogen	-0,779334584	0,007461125	0,131429392	-0,50208104	-0,251617522	0,7352096
1-(1-enyl-stearoyl)-2-linoleoyl-GPC (P-18:0/18:2)*		Lipid	Plasmalogen	0,188123548	1,045050582	1,794561064	0,844548252	1,388793423	0,526119612
1-(1-enyl-stearoyl)-2-linoleoyl-GPE (P-18:0/18:2)*		Lipid	Plasmalogen	-0,67773122	0,673796896	1,31309957	0,160387739	-0,052845534	0,744223771
1-(1-enyl-stearoyl)-GPE (P-18:0)*		Lipid	Lysoplasmalogen	-0,486501162	-0,015394912	0,820520504	-0,300911087	0,084475145	0,995086301
1-adrenoyl-GPC (22:4)*		Lipid	Lysolipid	-0,496190656	-0,774638729	0,027487793	-0,882617029	-0,134294147	0,120673642
1-arachidonoyl-GPC (20:4n6)*	HMDB10395	Lipid	Lysolipid	-0,314032686	-1,105321893	0,154586673	-0,833676168	-0,310027504	0,507406363
1-arachidonoyl-GPE (20:4n6)*	HMDB11517	Lipid	Lysolipid	-0,629137025	-1,473555093	-0,616876291	-0,362389906	-1,079471386	-0,182350592
1-arachidonoyl-GPI (20:4)*	HMDB61690	Lipid	Lysolipid	-0,680858194	-1,065298006	0,218004642	-0,694288044	-0,850967433	0,222392201
1-dihomo-linolenoyl-GPC (20:3n3 or 6)*	HMDB10394	Lipid	Lysolipid	-0,892815865	-1,37610579	-0,784855719	-1,310071348	-1,472393363	-0,385754167
1-dihomo-linolenoyl-GPE (20:3n3 or 6)*		Lipid	Lysolipid	-2,575813165	-2,578756542	-2,578078118	-2,576819039	-2,584101401	-2,575154635
1-dihomo-linoleoyl-GPC (20:2)*	HMDB10392	Lipid	Lysolipid	-1,138805115	-1,602483024	-1,086198924	-1,586743966	-1,515898782	-0,716796554
1-docosahexaenoyl-GPC (22:6)*	HMDB10404	Lipid	Lysolipid	-1,381624401	-0,924587953	-0,14263349	-0,960351262	-1,127829219	0,63940302
1-docosahexaenoyl-GPE (22:6)*		Lipid	Lysolipid	-1,071625407	-1,200680815	-0,449866698	-0,553867172	-0,93332486	0,255442856
1-docosapentaenoyl-GPC (22:5n3)*		Lipid	Lysolipid	-0,695808864	-1,48595637	0,405246128	-0,923334374	-0,473163368	0,807973091
1-docosapentaenoyl-GPC (22:5n6)*		Lipid	Lysolipid	-0,916056692	-0,541590756	-0,435557152	-1,157106954	-0,79346928	0,003446477

1-eicosapentaenoyl-GPC (20:5)*		Lipid	Lysolipid	-0,803355709	-0,920268183	0,034593131	-0,740407874	-0,567778304	1,287599725
1-eicosenoyl-GPC (20:1)*		Lipid	Lysolipid	-0,978085108	0,036226824	-0,244545748	-0,878806095	-0,33817514	0,179875152
1-linolenoyl-GPC (18:3)*		Lipid	Lysolipid	-2,481019159	-1,811764114	-1,428549782	-1,567375255	-2,607982789	-1,083178625
1-linoleoyl-2-arachidonoyl-GPC (18:2/20:4n6)*		Lipid	Phospholipid Metabolism	-0,569074977	-0,484903268	0,38243872	-0,340772002	-1,342483789	0,026909643
1-linoleoyl-GPA (18:2)*	HMDB07856	Lipid	Lysolipid	-0,121309659	0,027474998	1,010987743	0,35678872	-0,200647344	0,567651367
1-linoleoyl-GPE (18:2)*	HMDB11507	Lipid	Lysolipid	-0,764198604	-1,256098066	-0,474581251	0,187557161	-2,026085501	-1,067796677
1-linoleoyl-GPI (18:2)*		Lipid	Lysolipid	-0,7491271	-0,934230682	0,475863598	-0,198428865	-1,138094917	-1,157193979
1-linoleoylglycerol (18:2)		Lipid	Monoacylglycerol	0,420206865	-0,713296736	1,356643046	-0,022041956	-0,298790066	-0,325127247
1-methylhistidine	HMDB00001	Amino Acid	Histidine Metabolism	0,891652173	0,786980833	0,018747304	-0,388477773	-0,565887692	-0,070833831
1-methylimidazoleacetate	HMDB02820	Amino Acid	Histidine Metabolism	-1,020039563	-0,128435217	-0,274741567	-1,252597951	-1,607111764	-0,185725932
1-methylnicotinamide	HMDB00699	Cofactors and Vitamins	Nicotinate and Nicotinamide Metabolism	0,615450752	-0,029069448	-1,133899121	-1,476619446	-0,383429047	0,436839705
1-myristoyl-GPC (14:0)	HMDB10379	Lipid	Lysolipid	-0,168009048	-1,547353188	0,67657629	-1,011420305	-0,456181339	0,390397652
1-nonadecanoyl-GPC (19:0)		Lipid	Lysolipid	-0,329406172	-0,244656176	1,258487773	-0,017677053	0,621171018	1,186182909
1-oleoyl-2-dihomo-linolenoyl-GPC (18:1/20:3)*		Lipid	Phospholipid Metabolism	-1,568662046	-1,277019973	-1,376451533	-1,618761837	-2,16692537	-4,868709183
oleoyl-linoleoyl-glycerol (18:1/18:2) [1]		Lipid	Diacylglycerol	-0,56594103	-1,88969369	0,256464288	-0,899525378	-2,061653961	-2,404450734
oleoyl-linoleoyl-glycerol (18:1/18:2) [2]		Lipid	Diacylglycerol	-0,030060077	-1,230486044	0,338190703	-0,047981069	-1,241621925	-1,00969263
1-oleoyl-GPC (18:1)	HMDB02815	Lipid	Lysolipid	-0,59679734	0,113604935	0,743471614	-0,061310178	-0,508713346	0,854737544
1-oleoyl-GPE (18:1)	HMDB11506	Lipid	Lysolipid	-1,359872338	-0,035777996	-0,885813536	0,353585942	-1,808804311	-0,659565337
1-oleoyl-GPI (18:1)*		Lipid	Lysolipid	-1,274693222	0,457378985	0,311876409	0,159478293	-0,672409379	0,495489844
1-palmitoleoyl-GPC (16:1)*	HMDB10383	Lipid	Lysolipid	-0,604857553	-1,024266001	-0,047575263	-1,51518684	-1,342305322	-0,458420135
1-palmitoyl-2-adrenoyl-GPC (16:0/22:4)*		Lipid	Phospholipid Metabolism	-1,188861017	-1,318675093	-0,712918823	-1,365137945	-1,080236988	-0,298142579
1-palmitoyl-2-arachidonoyl-GPC (16:0/20:4n6)		Lipid	Phospholipid Metabolism	-0,807244062	-1,063324348	-0,480195097	-0,842188999	-0,6841708	0,116332744
1-palmitoyl-2-arachidonoyl-GPE (16:0/20:4)*	HMDB05323	Lipid	Phospholipid Metabolism	-0,815610729	-2,180585756	-0,87862411	-0,987784058	-0,583664844	-0,621636231
1-palmitoyl-2-docosahexaenoyl-GPC (16:0/22:6)		Lipid	Phospholipid Metabolism	-1,458189741	-0,665015037	-0,473286358	-0,819457955	-1,036412814	0,486495437
1-palmitoyl-2-docosahexaenoyl-GPE (16:0/22:6)*	HMDB05324	Lipid	Phospholipid Metabolism	-0,795740118	-1,529807711	-0,555489458	-0,753423219	-0,189977549	0,218089533
1-palmitoyl-2-eicosapentaenoyl-GPC (16:0/20:5)*		Lipid	Phospholipid Metabolism	-1,201422897	-0,92696018	-0,357851291	-1,104044435	-0,82016278	0,961415188
palmitoyl-linoleoyl-glycerol (16:0/18:2) [1]*	HMDB05207,HMDB07103	Lipid	Diacylglycerol	0,066493174	-1,672082795	0,699317789	-0,797125562	-1,489386116	-3,362914752
1-palmitoyl-2-linoleoyl-GPC (16:0/18:2)		Lipid	Phospholipid Metabolism	0,308666313	0,34190968	0,704910937	0,945558067	-0,381015038	0,514613045
palmitoyl-linoleoyl-glycerol (16:0/18:2) [2]*		Lipid	Diacylglycerol	0,326429587	-1,663476811	0,788591959	-0,237195551	-0,850766904	-1,229645971
1-palmitoyl-GPC (16:0)	HMDB10382	Lipid	Lysolipid	0,724652536	-0,42884291	1,705764664	0,032759562	0,251940752	1,307797913

1-palmitoyl-GPE (16:0)	HMDB11503	Lipid	Lysolipid	-1,182448339	-2,272496932	-0,196684859	-0,549280678	-0,791586144	0,135021591
1-palmitoyl-GPI (16:0)	HMDB61695	Lipid	Lysolipid	-2,104630914	-0,042660757	0,265717433	0,200512591	-2,103872265	0,153782769
1-palmityl-GPC (O-16:0)		Lipid	Lyso-phospho-ether	0,60836579	-0,338668696	0,771547564	-0,040107282	0,357802317	0,552206634
1-pentadecanoyl-GPC (15:0)*		Lipid	Lysolipid	1,295300571	-0,579339619	1,793979479	0,029534506	0,737723507	1,462679157
1-stearoyl-2-arachidonoyl-GPC (18:0/20:4)		Lipid	Phospholipid Metabolism	-1,459032196	-1,475401964	-0,570302207	-1,454599291	-1,191397563	-0,323377064
1-stearoyl-2-arachidonoyl-GPE (18:0/20:4)		Lipid	Phospholipid Metabolism	-0,869683383	-2,15800278	-0,613869707	-1,180455383	-0,745986336	-0,934617177
1-stearoyl-2-arachidonoyl-GPI (18:0/20:4)		Lipid	Phospholipid Metabolism	-0,771098091	-0,44393568	0,048153107	-0,627504954	-1,287467754	0,573179511
1-stearoyl-2-docosahexaenoyl-GPC (18:0/22:6)		Lipid	Phospholipid Metabolism	-2,640517287	-1,657808645	-1,026144937	-1,517658774	-2,106451131	-0,114053393
1-stearoyl-2-linoleoyl-GPC (18:0/18:2)*		Lipid	Phospholipid Metabolism	-1,255002636	-0,285864954	-0,118683242	0,032701275	-1,598491924	-0,308705727
1-stearoyl-2-linoleoyl-GPE (18:0/18:2)*		Lipid	Phospholipid Metabolism	-1,128371153	-1,049355466	-0,556898596	-0,22373936	-1,59833798	-1,726199308
1-stearoyl-GPC (18:0)	HMDB10384	Lipid	Lysolipid	-0,074648236	-0,52940841	1,481246795	-0,137609423	-0,317947622	0,920773333
1-stearoyl-GPE (18:0)	HMDB11130	Lipid	Lysolipid	-1,093351667	-2,020683745	0,406725693	-0,945927777	-0,976035517	-0,113426681
1-stearoyl-GPI (18:0)	HMDB61696	Lipid	Lysolipid	-0,218050775	-0,169085107	0,846086956	-0,132896894	-0,16282344	0,186671478
10-heptadecenoate (17:1n7)	HMDB60038	Lipid	Long Chain Fatty Acid	0,60506466	0,723069096	1,585788833	0,803701782	1,33383849	0,616992117
10-nonadecenoate (19:1n9)	HMDB13622	Lipid	Long Chain Fatty Acid	1,485971405	1,083497963	2,045900763	1,351672943	1,603187177	1,346875208
10-undecenoate (11:1n1)		Lipid	Medium Chain Fatty Acid	0,879609117	0,394575072	1,030852171	0,849271116	0,55355646	0,657635992
13-HODE + 9-HODE		Lipid	Fatty Acid, Monohydroxy	-0,030513623	0,640166645	0,105367743	0,685958137	-0,349508273	-1,203551381
15-methylpalmitate		Lipid	Fatty Acid, Branched	1,276559444	0,707001186	2,105940716	1,540958922	1,521800599	1,494595503
16-hydroxypalmitate	HMDB06294	Lipid	Fatty Acid, Monohydroxy	0,455876513	0,597302877	1,289597666	1,627079081	0,936158952	0,197389967
16a-hydroxy DHEA 3-sulfate		Lipid	Steroid	1,797830164	-0,649530082	0,403066835	0,759868852	1,450141978	-1,001131333
17-methylstearate		Lipid	Fatty Acid, Branched	1,56897785	0,926577603	2,193818992	1,107102402	1,635773841	1,682999463
2,3-dihydroxyisovalerate	HMDB12141	Xenobiotics	Food Component/Plant	-1,808742751	-1,809678076	1,028775294	-0,149407449	-0,666188088	-0,522556316
2-aminoheptanoate		Lipid	Fatty Acid, Amino	0,255996611	1,715322037	1,401461	2,025955075	1,759245468	1,488725023
2-aminophenol sulfate	HMDB61116	Xenobiotics	Chemical	-1,144773798	-0,359764816	0,476522105	0,777145674	-0,157979152	0,594928496
2-arachidonoyl-GPC (20:4)*	HMDB61699	Lipid	Lysolipid	-0,455254906	-0,968327032	0,318264676	-0,785122829	-0,252337474	0,439568903
2-hydroxy-3-methylvalerate	HMDB00317	Amino Acid	Leucine, Isoleucine and Valine Metabolism	1,318796137	0,822051554	1,303414509	0,941315046	0,617309929	-0,054041302
2-hydroxybutyrate/2-hydroxyisobutyrate		Amino Acid	Methionine, Cysteine, SAM and Taurine Metabolism	2,603193658	1,517301178	1,669890773	1,48620276	2,602641822	0,747199343
2-hydroxydecanoate		Lipid	Fatty Acid, Monohydroxy	-0,073722427	1,339245339	0,677766634	1,352508047	-1,286518558	-0,132241936
2-hydroxyglutarate	HMDB00606	Lipid	Fatty Acid, Dicarboxylate	-0,936159259	-0,956625896	-0,227681813	-1,512032828	-0,736652883	-0,128143485
2-hydroxyoctanoate	HMDB02264	Lipid	Fatty Acid, Monohydroxy	0,157141945	0,89712973	0,149576847	0,029216315	-1,656564303	-0,670797549
2-hydroxypalmitate	HMDB31057	Lipid	Fatty Acid, Monohydroxy	0,567529281	0,662427372	0,865053452	0,779898773	0,446348379	0,91522543

2-hydroxystearate		Lipid	Fatty Acid, Monohydroxy	0,071478019	0,227989039	1,174254939	0,464734205	-0,284053222	0,707929321
2-linoleoyl-GPC (18:2)*		Lipid	Lysolipid	-0,537554001	-0,591977778	0,37580121	0,160046661	-1,174352309	-0,490198467
2-methylbutyrylcarnitine (C5)	HMDB00378	Amino Acid	Leucine, Isoleucine and Valine Metabolism	-0,842279163	-0,553272469	0,173931646	-1,087061567	-1,066254454	-1,592226752
2-oleoyl-GPC (18:1)*	HMDB61701	Lipid	Lysolipid	-1,069813746	-0,293290921	0,705490183	-0,074018988	-0,775534537	0,720610324
2-oleoyl-GPE (18:1)*		Lipid	Lysolipid	-2,636762203	-2,640103886	-2,641096224	0,189335685	-2,641494207	-0,967203374
2-palmitoyl-GPC (16:0)*	HMDB61702	Lipid	Lysolipid	0,687238511	-0,810324007	1,384104824	-0,41874334	-0,392929372	1,109160641
2-palmitoyl-GPE (16:0)*		Lipid	Lysolipid	-0,589175466	-1,295259954	0,389114771	-0,511579173	-0,538661447	0,182018892
2-stearoyl-GPE (18:0)*		Lipid	Lysolipid	-0,974018504	-3,034941346	0,05073793	-1,035389527	-0,292951115	-1,350553403
21-hydroxypregnenolone disulfate	HMDB04026	Lipid	Steroid	1,234530155	-0,53994867	-0,162707146	0,715965369	0,934097919	-0,301980269
3-(3-hydroxyphenyl)propionate	HMDB00375	Amino Acid	Phenylalanine and Tyrosine Metabolism	-1,30067342	-1,303250628	-0,091314428	1,069227277	0,090429674	1,025946159
3-(4-hydroxyphenyl)lactate	HMDB00755	Amino Acid	Phenylalanine and Tyrosine Metabolism	-0,365783257	-0,243969962	0,821126165	-0,906590991	0,244436815	-0,592831578
3-aminoisobutyrate	HMDB03911	Nucleotide	Pyrimidine Metabolism, Thymine containing	0,033949585	0,671611149	0,636016393	-0,405182239	0,427740119	0,667518639
3-carboxy-4-methyl-5-propyl-2-furanpropanoate (CMPF)	HMDB61112	Lipid	Fatty Acid, Dicarboxylate	-0,386649381	0,434851903	1,084109206	0,797859285	0,02831988	0,931076373
3-hydroxy-2-ethylpropionate	HMDB00396	Amino Acid	Leucine, Isoleucine and Valine Metabolism	1,550855416	0,764643349	2,363716717	0,980543888	0,744741408	2,016076512
3-hydroxybutyrate (BHBA)	HMDB00357	Lipid	Ketone Bodies	1,839602784	2,613833711	3,000129609	2,539623654	2,434469824	2,315091476
3-hydroxydecanoate	HMDB02203	Lipid	Fatty Acid, Monohydroxy	0,514343786	1,477404164	1,185667279	0,711527181	1,017594827	0,652289696
3-hydroxyhippurate	HMDB06116	Xenobiotics	Benzoate Metabolism	-1,516738522	-0,395702166	-0,466489561	0,337070151	-0,037452265	0,607109391
3-hydroxyisobutyrate	HMDB00336	Amino Acid	Leucine, Isoleucine and Valine Metabolism	-2,668330078	-2,66698349	-2,665479607	-2,668799776	-2,658508343	-2,669333642
3-hydroxylaurate	HMDB00387	Lipid	Fatty Acid, Monohydroxy	1,541185171	1,924267198	1,612408996	0,905671891	1,661829426	1,549618206
3-hydroxyoctanoate	HMDB01954	Lipid	Fatty Acid, Monohydroxy	-0,276810686	0,607421909	0,393072458	0,614662252	0,232142801	-0,149887898
3-hydroxypyridine sulfate		Xenobiotics	Chemical	-1,515492286	-1,512864052	-1,516406819	-0,596801846	-1,510808011	-1,51375955
3-hydroxysebacate	HMDB00350	Lipid	Fatty Acid, Monohydroxy	-0,515286764	0,463948444	0,890581058	0,723823905	0,221723023	0,076363224
3-indoxyl sulfate	HMDB00682	Amino Acid	Tryptophan Metabolism	-0,031483767	-0,32957288	0,766314159	0,044128288	-1,156633914	-0,502015385
3-methoxytyrosine	HMDB01434	Amino Acid	Phenylalanine and Tyrosine Metabolism	-1,180197168	-0,022741536	-0,122875543	-0,535785407	-0,943275153	-0,983475973
3-methyl catechol sulfate (1)		Xenobiotics	Benzoate Metabolism	1,204232665	-0,857224489	-1,150363396	1,554810047	-0,242014143	-1,149335512
3-methyl-2-oxobutyrate	HMDB00019	Amino Acid	Leucine, Isoleucine and Valine Metabolism	1,158740933	1,580653314	0,992081005	1,060967914	1,027288039	1,163458302
3-methyl-2-oxovalerate	HMDB03736	Amino Acid	Leucine, Isoleucine and Valine Metabolism	1,256160644	1,54026973	0,8205058	0,68682855	0,508394283	0,580433946
3-methylglutarylcarnitine (2)	HMDB00552	Amino Acid	Lysine Metabolism	-0,194806739	0,21087905	0,040652912	-0,323552326	-1,267747955	-1,765827319
3-phosphoglycerate	HMDB00807	Carbohydrate	Glycolysis, Gluconeogenesis, and Pyruvate Metabolism	0,063613966	-0,958764222	-0,969411008	-1,681616403	-0,054321273	-1,089530695
4-allylphenol sulfate		Xenobiotics	Food Component/Plant	-0,316525589	-0,148818317	-0,159548828	0,756442565	-0,115091223	0,349926645

4-androsten-3beta,17beta-diol disulfate (1)	HMDB03818	Lipid	Steroid	1,364963363	-0,650485586	0,28503084	1,201426531	1,398696084	-0,592777157
4-androsten-3beta,17beta-diol disulfate (2)	HMDB03818	Lipid	Steroid	1,496279691	-1,243666219	-0,305764404	1,127587464	1,344424917	-1,966197424
4-ethylphenylsulfate		Xenobiotics	Benzoate Metabolism	1,109851142	-0,268800351	0,621014718	0,510855274	-0,320750979	-0,28667669
4-guanidinobutanoate	HMDB03464	Amino Acid	Guanidino and Acetamido Metabolism	-1,664245363	-0,676553333	0,414140307	-1,261514862	-1,56735071	-0,92147939
4-hydroxychlorothalonil		Xenobiotics	Chemical	-0,903919426	-1,915415058	-1,04114333	-1,404039687	-1,777557515	-0,609458016
4-hydroxyhippurate	HMDB13678	Xenobiotics	Benzoate Metabolism	-1,867497455	-0,417271126	-0,701333333	-1,256542711	-2,491523258	-0,940342545
4-hydroxyphenylpyruvate	HMDB00707	Amino Acid	Phenylalanine and Tyrosine Metabolism	-1,366270702	0,073580835	-0,526747841	-0,66932019	-1,0539338	-0,132311935
4-imidazoleacetate	HMDB02024	Amino Acid	Histidine Metabolism	1,121752878	0,444620359	-2,13178954	0,008743919	-0,33764733	0,47349261
4-methyl-2-oxopentanoate	HMDB00695	Amino Acid	Leucine, Isoleucine and Valine Metabolism	1,465459808	0,858606415	0,972313196	0,781595904	0,558643093	0,546008509
4-methylcatechol sulfate		Xenobiotics	Benzoate Metabolism	0,156442248	0,876389596	0,842855644	0,748005383	0,743832658	0,507078508
4-vinylphenol sulfate	HMDB04072	Xenobiotics	Benzoate Metabolism	-0,77206902	-0,349672337	0,183358026	0,55093081	-0,971558954	-0,634913274
5,6-dihydrothymine	HMDB00079	Nucleotide	Pyrimidine Metabolism, Thymine containing	-0,038051573	0,452078846	-0,44768725	-1,86384198	-0,08127244	0,42252824
5-dodecenoate (12:1n7)	HMDB00529	Lipid	Medium Chain Fatty Acid	0,237143513	0,310707461	0,518664479	0,667830737	0,572804706	0,422586721
5-hydroxylysine	HMDB00450	Amino Acid	Lysine Metabolism	-1,35687835	-0,341820594	-0,992488467	-0,634761377	-1,198852616	0,224513635
5-methylthioadenosine (MTA)	HMDB01173	Amino Acid	Polyamine Metabolism	-2,567546017	-0,956229529	-1,749317013	-1,68665257	-3,111542497	-1,658645355
5-methyluridine (ribothymidine)	HMDB00884	Nucleotide	Pyrimidine Metabolism, Uracil containing	-0,213855235	-0,153933654	0,744437017	-0,495485801	-1,16406424	0,413521791
5-oxoproline	HMDB00267	Amino Acid	Glutathione Metabolism	1,655782886	-0,307094371	-0,322362049	-0,013054291	1,009277135	0,519983068
5alpha-androstan-3beta,17beta-diol disulfate	HMDB00493	Lipid	Steroid	1,395376769	-0,958691138	-0,957065776	0,966317303	0,720146785	-0,956388559
5alpha-pregnan-3beta,20alpha-diol disulfate		Lipid	Steroid	1,288897073	-0,52027744	-2,014015115	1,105363306	0,943637787	-0,743475241
6-oxopiperidine-2-carboxylate		Amino Acid	Lysine Metabolism	-0,48132042	0,53149637	-0,297703956	-0,378210896	-1,194319668	-2,771794474
7-alpha-hydroxy-3-oxo-4-cholestenoate (7-Hoca)	HMDB12458	Lipid	Sterol	-0,809970367	-0,375327242	-1,105960969	-1,317812853	-1,147541868	-1,263591764
7-methylguanine	HMDB00897	Nucleotide	Purine Metabolism, Guanine containing	-0,363987994	-0,209457494	-0,256702169	-1,249521148	-0,843839646	-0,240852082
acetylcarnitine	HMDB00201	Lipid	Fatty Acid Metabolism(Acyl Carnitine)	0,61227044	1,023715942	1,164295565	0,926862671	0,216857382	0,228322575
adenine	HMDB00034	Nucleotide	Purine Metabolism, Adenine containing	-0,659948565	0,670377181	0,525428276	0,163867129	-0,903056276	0,213937806
adenosine 5'-monophosphate (AMP)	HMDB00045	Nucleotide	Purine Metabolism, Adenine containing	-1,279200591	-1,173071412	-1,230926454	-1,06299566	0,31284147	-0,94784321
alanine	HMDB00161	Amino Acid	Alanine and Aspartate Metabolism	-1,212968976	-1,753518899	-1,381858122	-1,581407855	-1,169214187	-1,463417022
allantoin	HMDB00462	Nucleotide	Purine Metabolism, (Hypo)Xanthine/Inosine containing	-2,249244149	-1,150982371	0,516357996	-1,194176771	-2,187483785	-0,816308039
alpha-hydroxyisocaproate	HMDB00746	Amino Acid	Leucine, Isoleucine and Valine Metabolism	2,302621267	0,84147864	0,369156151	0,591392156	1,178674769	0,504865936

alpha-hydroxyisovalerate	HMDB00407	Amino Acid	Leucine, Isoleucine and Valine Metabolism	1,697829465	0,937906212	2,399693032	1,694070133	2,058843999	0,178852426
alpha-ketoglutarate	HMDB00208	Energy	TCA Cycle	-0,323645497	-0,307960921	0,828143801	0,083471802	0,635549914	0,981738201
alpha-tocopherol	HMDB01893	Cofactors and Vitamins	Tocopherol Metabolism	-0,357507601	-0,336053455	0,201746349	-0,263764207	-0,69797966	-0,928610474
androsterone sulfate	HMDB02759	Lipid	Steroid	1,473607963	-0,690017084	-1,286871476	1,1059951	0,366005985	-0,693748275
arabitol/xylitol		Carbohydrate	Pentose Metabolism	-0,812898708	-0,44262781	0,674656893	-0,624915879	-0,227768958	0,260274172
arabonate/xylonate		Carbohydrate	Pentose Metabolism	-1,323011439	-0,507864136	1,210511534	0,373760944	-1,796765801	1,337563865
arachidate (20:0)	HMDB02212	Lipid	Long Chain Fatty Acid	-0,121194996	2,623204589	1,552750278	0,647287769	0,067744204	0,480127369
arachidonate (20:4n6)	HMDB01043	Lipid	Polyunsaturated Fatty Acid (n3 and n6)	0,50466071	-0,009083313	1,155795856	-0,295957264	0,280913747	0,623575061
arginine	HMDB00517	Amino Acid	Urea cycle; Arginine and Proline Metabolism	-1,787427591	-1,360744683	-0,205600826	-0,431209758	-0,287881868	-0,814128042
asparagine	HMDB00168	Amino Acid	Alanine and Aspartate Metabolism	-0,230316913	-0,697605529	-0,109878748	0,379498742	-0,147143379	-0,967168345
aspartate	HMDB00191	Amino Acid	Alanine and Aspartate Metabolism	-0,259925341	-0,989758638	-0,672364988	-0,92882631	-0,381660338	-1,143258855
azelate (nonanedioate)	HMDB00784	Lipid	Fatty Acid, Dicarboxylate	-0,385216306	0,252373628	-0,842680469	0,180379733	-0,468235506	-1,311518937
beta-hydroxyisovalerate	HMDB00754	Amino Acid	Leucine, Isoleucine and Valine Metabolism	0,059908252	1,71645536	0,700719437	-0,204779536	0,563587115	1,262516228
betaine	HMDB00043	Amino Acid	Glycine, Serine and Threonine Metabolism	-0,483182822	0,804110904	2,068417435	0,542809182	-0,306245264	0,001895681
biliverdin	HMDB01008	Cofactors and Vitamins	Hemoglobin and Porphyrin Metabolism	0,632952319	-0,595698424	0,41560981	0,712142869	0,655993854	-0,280511422
butyrylcarnitine	HMDB02013	Lipid	Fatty Acid Metabolism (also BCAA Metabolism)	-0,837176461	1,099834171	-0,637395699	-0,822083974	-1,981118512	-0,600960913
C-glycosyltryptophan		Amino Acid	Tryptophan Metabolism	-1,132190411	-0,846925103	-0,690088291	-1,386425686	-1,789396666	-1,138811542
caprate (10:0)	HMDB00511	Lipid	Medium Chain Fatty Acid	-0,505496444	-0,587145277	-0,166310162	1,300103026	-0,558007122	-0,265152496
carnitine	HMDB00062	Lipid	Carnitine Metabolism	-0,47101138	-1,588787844	-1,547466012	-0,507679194	-2,335706438	-2,770991675
catechol sulfate	HMDB59724	Xenobiotics	Benzoate Metabolism	-0,802506325	0,033895145	-0,106121272	-0,029975313	-0,718631816	0,279950759
chenodeoxycholate	HMDB00518	Lipid	Primary Bile Acid Metabolism	-0,596891974	-0,846330537	-0,36934321	-0,47769262	-1,21409034	-0,558693327
cholate	HMDB00619	Lipid	Primary Bile Acid Metabolism	-1,47490246	0,22751057	0,205805975	0,422540854	-1,756029303	0,806232939
cholesterol	HMDB00067	Lipid	Sterol	-1,533608516	-0,422149739	0,217979501	-0,699536786	-1,040018059	-0,144266776
choline	HMDB00097	Lipid	Phospholipid Metabolism	-1,361555696	-0,025148898	-0,049471684	-1,530940403	-1,065220611	0,026585156
choline phosphate	HMDB01565	Lipid	Phospholipid Metabolism	-0,6409668	-0,041618056	0,106554768	-0,26938477	-1,633771346	-0,046818241
cis-4-decenoyl carnitine		Lipid	Fatty Acid Metabolism(Acyl Carnitine)	1,040798188	-0,483904981	-0,033759621	0,441005601	-0,578654643	-0,569279704
citrate	HMDB00094	Energy	TCA Cycle	-0,02070815	0,359539279	0,79904857	1,027897842	0,102213756	0,538651751
citrulline	HMDB00904	Amino Acid	Urea cycle; Arginine and Proline Metabolism	0,036446034	0,298546913	1,21485881	-0,961301653	-0,448770683	0,739183191
cortisol	HMDB00063	Lipid	Steroid	-0,374582835	0,074369932	-0,263377213	-0,823116664	0,131033997	0,488182258
cortisone	HMDB02802	Lipid	Steroid	0,46084314	0,314989212	-0,367800891	-0,281573249	0,776697492	0,801852446

creatine	HMDB00064	Amino Acid	Creatine Metabolism	-3,739297378	0,33284462	-0,410272868	-0,762423365	-1,504476718	-0,117379741
creatinine	HMDB00562	Amino Acid	Creatine Metabolism	0,809811677	-0,71844289	0,31000098	-0,196821251	-0,134844738	-0,663226935
cys-gly, oxidized		Amino Acid	Glutathione Metabolism	1,231295861	0,118312733	-0,344162919	0,008306568	0,337738088	0,585619666
cystathionine	HMDB00099	Amino Acid	Methionine, Cysteine, SAM and Taurine Metabolism	-0,45678226	-0,551885518	-1,163472546	-1,296142862	-1,087861416	-0,900649788
cysteine	HMDB00574	Amino Acid	Methionine, Cysteine, SAM and Taurine Metabolism	-0,481465478	-0,25644965	0,336089078	-0,367129238	-0,482621556	-0,146371488
cysteine s-sulfate	HMDB00731	Amino Acid	Methionine, Cysteine, SAM and Taurine Metabolism	0,143182257	-2,333982107	-1,31484598	-2,192296786	-0,530479319	-2,983315165
decanoylcarnitine	HMDB00651	Lipid	Fatty Acid Metabolism(Acyl Carnitine)	0,539984479	0,900709093	0,220752623	1,49721982	-0,384993662	0,197885927
dehydroisoandrosterone sulfate (DHEA-S)	HMDB01032	Lipid	Steroid	1,641755662	-0,585246316	-0,549330192	0,86623895	0,827610664	-1,125736014
deoxycarnitine	HMDB01161	Lipid	Carnitine Metabolism	0,87725418	-0,744346894	-0,060440433	-0,394225833	-0,654278957	-0,688867178
deoxycholate	HMDB00626	Lipid	Secondary Bile Acid Metabolism	0,245408579	-2,531289576	1,149820845	0,973019531	-0,777485658	-0,154606491
dihomo-linoleate (20:2n6)	HMDB05060	Lipid	Polyunsaturated Fatty Acid (n3 and n6)	0,927360534	0,836813959	0,947444946	0,119556774	1,160906972	-0,243116667
dihomo-linolenate (20:3n3 or n6)	HMDB02925	Lipid	Polyunsaturated Fatty Acid (n3 and n6)	0,351282364	0,044491116	0,47492751	-0,773440684	-0,174633318	-0,349401557
dimethylarginine (SDMA + ADMA)	HMDB01539	Amino Acid	Urea cycle; Arginine and Proline Metabolism	-1,015408911	-0,52401574	-0,654153801	-1,253905405	-1,005011101	-0,094319076
dimethylglycine	HMDB00092	Amino Acid	Glycine, Serine and Threonine Metabolism	-0,66968046	1,17105255	1,237193831	0,105058836	0,889837651	-0,292216442
docosadienoate (22:2n6)	HMDB61714	Lipid	Polyunsaturated Fatty Acid (n3 and n6)	0,535677274	0,552897653	0,92609439	0,270878362	0,998161569	-0,033939336
docosahexaenoate (DHA; 22:6n3)	HMDB02183	Lipid	Polyunsaturated Fatty Acid (n3 and n6)	-0,271727935	0,287455839	0,806991598	-0,442490813	-0,04316431	0,864302415
docosapentaenoate (n3 DPA; 22:5n3)	HMDB01976	Lipid	Polyunsaturated Fatty Acid (n3 and n6)	0,514481935	0,201523495	1,769259977	-0,058104175	0,688973614	0,773769107
docosapentaenoate (n6 DPA; 22:5n6)	HMDB01976	Lipid	Polyunsaturated Fatty Acid (n3 and n6)	0,503686318	0,509987605	0,081961876	-1,070029067	0,254298132	0,583101854
dopamine sulfate (2)		Amino Acid	Phenylalanine and Tyrosine Metabolism	-1,616443171	-0,096805784	-0,108540286	0,282864469	-1,398309865	-0,803612768
EDTA	HMDB15109	Xenobiotics	Chemical	-0,237309996	-0,700447693	-0,759926326	-0,689110582	-0,686383681	-0,850805148
eicosanodioate		Lipid	Fatty Acid, Dicarboxylate	-0,400903511	0,123851032	0,640656699	0,848883374	-1,188844443	-0,950494992
eicosapentaenoate (EPA; 20:5n3)	HMDB01999	Lipid	Polyunsaturated Fatty Acid (n3 and n6)	-0,270831609	-0,508022827	0,922297878	-0,676508498	-0,238921801	0,896500744
eicosenoate (20:1)	HMDB02231	Lipid	Long Chain Fatty Acid	0,764384515	2,049981288	1,425021767	0,813966293	1,522089656	0,330568221
ergothioneine	HMDB03045	Xenobiotics	Food Component/Plant	-1,651046696	-1,653707061	0,892567247	0,872310279	0,177681214	1,052595365
erythritol	HMDB02994	Xenobiotics	Food Component/Plant	2,670007	0,771538231	-1,53372835	-1,323444288	-2,258604236	-1,274213346
erythronate*	HMDB00613	Carbohydrate	Aminosugar Metabolism	-0,767119951	-0,498053622	0,577267661	-1,260483385	-2,511668883	-0,135351334
ethylmalonate	HMDB00622	Amino Acid	Leucine, Isoleucine and Valine Metabolism	-1,139459697	1,757302493	0,053833288	-1,30759863	-1,10139061	0,809648289
eugenol sulfate		Xenobiotics	Food Component/Plant	-0,117123045	-0,21889985	0,567930192	-0,071300797	-0,971174903	-0,751771139
ferulic acid 4-sulfate	HMDB29200	Xenobiotics	Food Component/Plant	-0,93417132	-0,939773658	-0,934256262	-0,940912602	-0,935116834	-0,935910762

fructose	HMDB00660	Carbohydrate	Fructose, Mannose and Galactose Metabolism	-1,213082291	-0,629472656	0,402945814	-0,307643299	-0,859943355	-0,041637043
fumarate	HMDB00134	Energy	TCA Cycle	1,441766684	0,426795886	0,457513513	0,125734884	1,158854379	2,027146922
gamma-CEHC	HMDB01931	Cofactors and Vitamins	Tocopherol Metabolism	-0,699465119	0,090096032	0,339650236	-0,450779495	-0,539445207	0,284881378
gamma-glutamyl-epsilon-lysine	HMDB03869	Peptide	Gamma-glutamyl Amino Acid	-0,065383947	-0,320263347	-0,547613691	-0,521898499	-0,89611524	-0,728118743
gamma-glutamylalanine	HMDB29142	Peptide	Gamma-glutamyl Amino Acid	-1,100390036	-2,311484654	-1,783501627	-1,660252893	-1,263082252	-1,171423135
gamma-glutamylglutamate	HMDB11737	Peptide	Gamma-glutamyl Amino Acid	-0,793581465	-0,353994229	-0,528398204	-0,501173013	-1,397191616	-0,336129396
gamma-glutamylglutamine	HMDB11738	Peptide	Gamma-glutamyl Amino Acid	0,154798127	-0,841853009	-0,783585326	-0,329521209	-0,406262621	-0,181875985
gamma-glutamylglycine	HMDB11667	Peptide	Gamma-glutamyl Amino Acid	-0,552141201	-0,471514249	0,920621851	0,153835958	0,134439083	0,785172944
gamma-glutamylisoleucine*	HMDB11170	Peptide	Gamma-glutamyl Amino Acid	0,227145366	0,402446252	0,599209175	-0,365226416	-0,127853139	-0,209672295
gamma-glutamylleucine	HMDB11171	Peptide	Gamma-glutamyl Amino Acid	0,892160196	0,776378827	0,534342718	0,010798589	0,297603095	-0,017452589
gamma-glutamylmethionine	HMDB29155	Peptide	Gamma-glutamyl Amino Acid	-1,22290965	-0,834128089	-1,042652061	-0,818519792	-0,852953971	-1,044487237
gamma-glutamylthreonine	HMDB29159	Peptide	Gamma-glutamyl Amino Acid	-0,205891302	-0,269814087	-0,14941844	-0,195277676	-0,376347427	-1,172017685
gamma-glutamyltyrosine	HMDB11741	Peptide	Gamma-glutamyl Amino Acid	-2,004681761	-0,818511046	-0,817142031	-2,065669431	-1,236845496	-0,634424387
gamma-glutamylvaline	HMDB11172	Peptide	Gamma-glutamyl Amino Acid	0,453705419	0,459303917	0,285387302	-0,503355524	-0,477125199	-0,526565222
gluconate	HMDB00625	Xenobiotics	Food Component/Plant	-1,639571256	-1,647354427	-1,823979786	-1,707652959	-2,154022183	-1,120763359
glucose	HMDB00122	Carbohydrate	Glycolysis, Gluconeogenesis, and Pyruvate Metabolism	-1,362692926	-1,237683174	-0,856665409	-1,306018455	-1,306690448	-1,252827841
glucuronate	HMDB00127	Carbohydrate	Aminosugar Metabolism	-0,931478008	-0,746465042	0,161596095	-0,849594797	0,008150358	0,181887738
glutamate	HMDB00148	Amino Acid	Glutamate Metabolism	-0,574372832	-0,641374861	-0,55413606	-0,369529736	-0,619489073	-0,831485343
glutamine	HMDB00641	Amino Acid	Glutamate Metabolism	-0,284889269	-1,340206414	-0,600732579	-0,389457838	-0,605232691	-1,069539183
glutarate (pentanedioate)	HMDB00661	Amino Acid	Lysine Metabolism	-0,507943487	0,520194906	-0,608037589	-0,452011745	-0,60341802	-0,421255447
glutarylcarnitine (C5)	HMDB13130	Amino Acid	Lysine Metabolism	0,06183642	-1,042559493	-0,846526826	-1,489971979	-0,938123829	-1,061103721
glycerate	HMDB00139	Carbohydrate	Glycolysis, Gluconeogenesis, and Pyruvate Metabolism	-1,253210123	0,049235606	0,81953794	0,126423672	-1,842721127	0,662575894
glycerol	HMDB00131	Lipid	Glycerolipid Metabolism	-0,680434838	1,030895557	1,014536359	1,014550797	0,552578988	1,225236079
glycerol 3-phosphate	HMDB00126	Lipid	Glycerolipid Metabolism	0,8622927	0,238127849	0,637694315	0,25286386	0,002777806	0,481565323
glycerophosphoethanolamine	HMDB00114	Lipid	Phospholipid Metabolism	-0,489320825	0,058704372	0,25366358	0,221343863	-0,614292019	0,0296213
glycerophosphorylcholine (GPC)	HMDB00086	Lipid	Phospholipid Metabolism	0,910084438	0,211013236	0,974706556	0,7280203	0,545601073	1,080714531
glycine	HMDB00123	Amino Acid	Glycine, Serine and Threonine Metabolism	-0,942323365	-0,836349921	0,8455778	0,099041871	0,838123228	0,765624519
glycochenodeoxycholate	HMDB00637	Lipid	Primary Bile Acid Metabolism	-1,452727448	-0,615720758	-0,909734613	-0,459044093	-3,393723481	-0,834756189
glycocholate	HMDB00138	Lipid	Primary Bile Acid Metabolism	-1,38072955	-0,328695166	-0,384034577	-0,339297571	-2,364634346	-0,451506657
glycocholenate sulfate*		Lipid	Secondary Bile Acid Metabolism	0,899999111	0,671464014	-0,288602841	0,323646141	-0,425303503	-1,382625166
glycodeoxycholate	HMDB00631	Lipid	Secondary Bile Acid Metabolism	-0,016859641	-2,977625187	0,526012765	0,596898108	-1,552667858	-0,425316979

glycohyocholate		Lipid	Secondary Bile Acid Metabolism	-0,202936317	0,426675104	0,138713206	0,066347225	-1,414399538	-0,034134866
glycolithocholate sulfate*	HMDB02639	Lipid	Secondary Bile Acid Metabolism	0,774605179	-0,544736285	0,505499423	0,352568381	-0,310373794	-0,886097786
glycoursodeoxycholate	HMDB00708	Lipid	Secondary Bile Acid Metabolism	-1,911021267	0,325716161	-0,216528634	0,126878848	-2,66532197	-1,004235939
guanidinoacetate	HMDB00128	Amino Acid	Creatine Metabolism	1,589938068	0,172942466	2,163199606	0,997241092	2,450809225	2,044568323
gulonate*	HMDB03290	Cofactors and Vitamins	Ascorbate and Aldarate Metabolism	-5,81915532	-0,498148084	-0,099256946	-1,17440259	-0,905465319	-0,002667306
heme	HMDB03178	Cofactors and Vitamins	Hemoglobin and Porphyrin Metabolism	1,313650974	0,162318713	0,056591508	-0,711448924	0,686277495	-3,108167145
hexanoylcarnitine	HMDB00705	Lipid	Fatty Acid Metabolism(Acyl Carnitine)	0,05382045	0,045895816	0,126143491	0,64119677	-0,309695365	-0,262995604
hippurate	HMDB00714	Xenobiotics	Benzoate Metabolism	-0,572052323	-1,323303551	0,554480493	0,285884103	-0,277625046	0,521310442
histidine	HMDB00177	Amino Acid	Histidine Metabolism	-0,829427363	-2,354197947	-1,575958817	-1,296324257	-1,525298412	-1,520574854
homoarginine	HMDB00670	Amino Acid	Urea cycle; Arginine and Proline Metabolism	0,838007251	-0,6228827	-1,66132972	-0,570143328	-0,055893406	-1,666874865
hydroquinone sulfate	HMDB02434	Xenobiotics	Drug	-1,200026795	0,634802965	0,606275454	1,216071065	-1,200675824	0,035523589
3-hydroxybutyrylcarnitine (1)	HMDB13127	Lipid	Fatty Acid Metabolism(Acyl Carnitine)	1,35659694	2,129765743	2,82084422	2,315072025	1,771389706	1,565539997
hyocholate	HMDB00760	Lipid	Secondary Bile Acid Metabolism	-0,317580676	0,51778816	0,735731997	0,813298003	-1,003213249	0,358067163
hypotaurine	HMDB00965	Amino Acid	Methionine, Cysteine, SAM and Taurine Metabolism	-1,800530042	-0,464127001	-0,293843814	-1,206387804	-0,592096384	-0,66312701
hypoxanthine	HMDB00157	Nucleotide	Purine Metabolism, (Hypo)Xanthine/Inosine containing	0,710781784	0,499599167	-0,671271582	-0,321838331	0,182812979	0,485686374
imidazole lactate	HMDB02320	Amino Acid	Histidine Metabolism	0,372677418	-1,062015608	1,68570539	-0,079580985	0,097358625	-0,317055876
imidazole propionate	HMDB02271	Amino Acid	Histidine Metabolism	-1,363079754	-0,081756069	-0,730964426	-1,14073071	-1,155108986	-0,303843727
iminodiacetate (IDA)	HMDB11753	Xenobiotics	Chemical	-2,077627733	-2,409666715	-1,454283072	-1,894788514	-2,113431828	-2,222674899
indole-3-carboxylic acid	HMDB03320	Amino Acid	Tryptophan Metabolism	2,075723069	1,329247037	0,08357324	0,588334865	-0,546705281	0,11524119
indoleacetate	HMDB00197	Amino Acid	Tryptophan Metabolism	0,368829028	-0,01652236	-0,504208229	-0,638973148	-0,850936812	-0,225808211
indolelactate	HMDB00671	Amino Acid	Tryptophan Metabolism	0,202939289	0,107168392	1,194183029	0,089434276	-0,559810753	-0,520926272
indolepropionate	HMDB02302	Amino Acid	Tryptophan Metabolism	0,26883284	-0,626548892	0,539866699	0,72398357	0,149866568	0,663249128
inosine	HMDB00195	Nucleotide	Purine Metabolism, (Hypo)Xanthine/Inosine containing	1,669183711	1,390911287	1,309475151	1,441618408	2,392086265	1,266312088
inosine 5'-monophosphate (IMP)	HMDB00175	Nucleotide	Purine Metabolism, (Hypo)Xanthine/Inosine containing	-0,909774666	-0,909782418	-0,910628835	-0,909153911	-0,910270067	-0,908525499
isobutyrylcarnitine	HMDB00736	Amino Acid	Leucine, Isoleucine and Valine Metabolism	-1,59920948	-0,184649886	-1,008234758	-1,196104319	-0,98498376	-1,286258078
isoleucine	HMDB00172	Amino Acid	Leucine, Isoleucine and Valine Metabolism	0,966086877	0,825415241	0,639868549	-0,026027397	0,925826821	-0,248067501
isoleucylglycine		Peptide	Dipeptide	-0,554183885	0,768074258	-0,11669404	-0,257547267	-2,272274693	0,403192761
isoleucylleucine/leucylisoleucine		Peptide	Dipeptide	-0,578996039	0,136990276	-1,016562003	-0,86155738	-2,279092524	-1,240253944

isovalerylcarnitine	HMDB00688	Amino Acid	Leucine, Isoleucine and Valine Metabolism	-1,461917477	-1,229728411	-1,937931904	-1,60859287	-1,977460117	-2,08091455
isovalerylglycine	HMDB00678	Amino Acid	Leucine, Isoleucine and Valine Metabolism	-1,187691733	0,536826809	-1,188990022	-0,540806405	0,304485327	0,424861615
kynurenate	HMDB00715	Amino Acid	Tryptophan Metabolism	-1,155564037	0,367282634	0,016736222	-0,68301263	-1,258036532	-0,135629727
kynurenine	HMDB00684	Amino Acid	Tryptophan Metabolism	-0,817881923	-0,006026927	-0,645557333	-0,861078671	-1,731033215	-1,199534596
lactate	HMDB00190	Carbohydrate	Glycolysis, Gluconeogenesis, and Pyruvate Metabolism	0,231115816	-0,410455875	-0,545558677	-0,912283679	0,752174442	0,69513606
laurate (12:0)	HMDB00638	Lipid	Medium Chain Fatty Acid	-0,122042786	-0,589054018	0,394915917	1,083646864	-0,071788389	0,158272173
laurylcarnitine	HMDB02250	Lipid	Fatty Acid Metabolism(Acyl Carnitine)	0,431928145	0,638419198	0,572998922	0,815184879	0,547446766	0,879919472
leucine	HMDB00687	Amino Acid	Leucine, Isoleucine and Valine Metabolism	1,643977397	0,848730174	0,578785526	0,343053754	1,401865855	0,106727976
leucylglycine		Peptide	Dipeptide	-1,295341249	0,164200763	-1,295231134	-1,290216199	-1,29053811	-1,291382563
linoleate (18:2n6)	HMDB00673	Lipid	Polyunsaturated Fatty Acid (n3 and n6)	0,669695966	0,396734269	1,048046529	0,257881374	0,614582231	-0,352441732
linolenate [alpha or gamma; (18:3n3 or 6)]	HMDB03073	Lipid	Polyunsaturated Fatty Acid (n3 and n6)	-0,98684828	-0,642164216	0,043710753	-0,72969472	-0,364264398	-1,029621815
linoleoylcarnitine*	HMDB06469	Lipid	Fatty Acid Metabolism(Acyl Carnitine)	0,779027265	-0,344319803	0,234232442	-0,456662723	0,610264041	-0,428954221
lysine	HMDB00182	Amino Acid	Lysine Metabolism	0,456009963	-0,394538296	-1,41300992	-0,43478723	0,345617528	-1,038899357
maleate	HMDB00176	Lipid	Fatty Acid, Dicarboxylate	-1,962608045	-1,232060781	-0,239588815	-1,181795537	-1,583861342	-0,566360201
mannitol/sorbitol	HMDB00247	Carbohydrate	Fructose, Mannose and Galactose Metabolism	-1,681184489	-1,477148806	-1,544336935	-0,75708565	-1,326875458	-1,605903637
mannose	HMDB00169	Carbohydrate	Fructose, Mannose and Galactose Metabolism	1,61085699	0,288895403	0,195720687	0,74007805	1,649147693	-0,091854941
margarate (17:0)	HMDB02259	Lipid	Long Chain Fatty Acid	1,3982408	1,00848763	2,152898164	1,519158081	1,695152952	1,030583529
mead acid (20:3n9)	HMDB10378	Lipid	Polyunsaturated Fatty Acid (n3 and n6)	0,153799924	0,433288583	1,134892445	-0,3555326	0,660468699	0,730909082
methionine	HMDB00696	Amino Acid	Methionine, Cysteine, SAM and Taurine Metabolism	-1,226911615	-0,892731306	-0,717452202	-0,992565222	-0,476171409	-1,102827088
methionine sulfone		Amino Acid	Methionine, Cysteine, SAM and Taurine Metabolism	0,06721624	0,180139404	2,453667288	-0,051303569	-0,256380376	0,738994687
methionine sulfoxide	HMDB02005	Amino Acid	Methionine, Cysteine, SAM and Taurine Metabolism	-2,253406345	-1,00358727	-0,713804995	-0,673749005	-1,847858389	-0,905526622
methyl glucopyranoside (alpha + beta)		Xenobiotics	Food Component/Plant	-1,114851866	-1,530672178	-1,532327059	-0,64106752	-1,527563896	-0,158538336
methyl-4-hydroxybenzoate sulfate		Xenobiotics	Benzoate Metabolism	-0,265766039	-0,15301078	-1,250010071	-0,853221909	-1,331080322	0,639287743
methylsuccinate	HMDB01844	Amino Acid	Leucine, Isoleucine and Valine Metabolism	-1,390920513	-0,173809782	0,335293975	-1,055112809	-1,053833953	-0,145686736
myo-inositol	HMDB00211	Lipid	Inositol Metabolism	-2,343953379	-0,877051857	-0,198162581	-1,56187413	-2,05028941	-0,336353191
myristate (14:0)	HMDB00806	Lipid	Long Chain Fatty Acid	0,904767544	0,412912848	1,484040694	1,527434332	1,288870569	1,020325001
myristoleate (14:1n5)	HMDB02000	Lipid	Long Chain Fatty Acid	0,644033048	0,580578483	0,984286692	0,880161493	1,052405055	0,776125409
myristoleoylcarnitine*		Lipid	Fatty Acid Metabolism(Acyl Carnitine)	0,189993596	0,913615476	0,504013414	0,482168552	0,857038799	0,290766833
myristoylcarnitine	HMDB05066	Lipid	Fatty Acid Metabolism(Acyl Carnitine)	0,42667693	0,252703941	1,227717563	0,756250012	1,123338991	1,334952182

N-(2-furoyl)glycine	HMDB00439	Xenobiotics	Food Component/Plant	-1,473948728	-1,474963157	-1,174888854	-1,144148473	-1,474065715	-1,473179855
N-acetyl-aspartyl-glutamate (NAAG)	HMDB01067	Amino Acid	Glutamate Metabolism	-1,154516865	0,286097737	0,155566437	0,119220517	-1,85992456	0,935243621
N-acetyl-beta-alanine		Nucleotide	Pyrimidine Metabolism, Uracil containing	-0,847434468	0,405594403	-0,090073644	-0,073335241	-1,739375767	1,53062844
N-acetylalanine	HMDB00766	Amino Acid	Alanine and Aspartate Metabolism	-0,371342045	0,289600179	0,157647056	-0,958387324	-0,944463806	0,244686242
N-acetylarginine	HMDB04620	Amino Acid	Urea cycle; Arginine and Proline Metabolism	-0,633153202	-0,805962407	0,310296442	-0,219356851	-1,058779721	-0,651258634
N-acetylaspartate (NAA)	HMDB00812	Amino Acid	Alanine and Aspartate Metabolism	-1,330822318	-1,051273936	-0,825583821	-0,959709226	-1,564756284	-0,519297524
N-acetylcarnosine	HMDB12881	Peptide	Dipeptide Derivative	1,383749167	-0,383492498	0,67315732	0,440631492	-0,249529992	-1,189221631
N-acetylglycine	HMDB00532	Amino Acid	Glycine, Serine and Threonine Metabolism	1,583264031	2,25301139	2,390683469	1,994876379	2,502317186	3,684576699
N-acetylhistidine	HMDB32055	Amino Acid	Histidine Metabolism	-1,546439971	-0,217938633	0,081398785	-0,603828223	-1,764442094	0,554681468
N-acetylleucine	HMDB11756	Amino Acid	Leucine, Isoleucine and Valine Metabolism	-0,482102559	0,168376202	1,021828618	-0,308047315	-0,162275313	0,133649498
N-acetylmethionine	HMDB11745	Amino Acid	Methionine, Cysteine, SAM and Taurine Metabolism	-0,641170903	0,022558397	-1,765737259	-0,983231302	-1,198792036	-0,536355552
N-acetylneuraminate	HMDB00230	Carbohydrate	Aminosugar Metabolism	-1,383320544	-0,243222891	-0,940249107	-1,118936265	-2,86319974	-0,02802899
N-acetylphenylalanine	HMDB00512	Amino Acid	Phenylalanine and Tyrosine Metabolism	-0,018945408	0,050138589	0,422988562	0,061362193	0,087045276	1,707202281
N-acetylputrescine	HMDB02064	Amino Acid	Polyamine Metabolism	-1,842030513	-0,734667177	-0,957763031	-1,732042153	-2,401448828	-0,765500879
N-acetylserine	HMDB02931	Amino Acid	Glycine, Serine and Threonine Metabolism	-1,77473797	-0,633914694	-1,019314156	-1,645718247	-1,535929132	-0,338352092
N-acetyltaurine		Amino Acid	Methionine, Cysteine, SAM and Taurine Metabolism	-0,614049366	0,328205636	0,234726107	0,196804617	-0,53693253	1,154990365
N-acetylthreonine		Amino Acid	Glycine, Serine and Threonine Metabolism	-0,353883496	-0,307298369	-1,032563513	-1,315850333	-1,864151626	0,175394553
N-acetyltryptophan	HMDB13713	Amino Acid	Tryptophan Metabolism	-0,682079953	-0,49200687	-0,680729271	-0,990074006	-0,574168486	2,174114313
N-acetyltyrosine	HMDB00866	Amino Acid	Phenylalanine and Tyrosine Metabolism	-2,29143479	-2,305113702	-0,379393256	-2,298968023	-0,14261843	1,206068005
N-acetylvaline	HMDB11757	Amino Acid	Leucine, Isoleucine and Valine Metabolism	-0,309106486	0,579124867	-0,939037267	-0,477795056	-1,900396079	0,667298212
N-delta-acetylornithine		Amino Acid	Urea cycle; Arginine and Proline Metabolism	-0,36300242	-0,011561341	-0,268054659	-1,709485377	-2,606571627	0,947691325
N-formylmethionine	HMDB01015	Amino Acid	Methionine, Cysteine, SAM and Taurine Metabolism	-0,870846822	-0,457294298	-0,004035807	-1,146089589	-1,483304257	-0,768607981
N-formylphenylalanine		Amino Acid	Phenylalanine and Tyrosine Metabolism	-1,691767083	-1,689720687	-1,691249499	-1,684949606	-1,686159384	-1,686889518
N-methylproline		Amino Acid	Urea cycle; Arginine and Proline Metabolism	0,421883679	-0,033351041	-0,393420388	-0,437992077	-0,136644557	1,052362016
N-palmitoylglycine		Lipid	Fatty Acid Metabolism(Acyl Glycine)	0,911287279	-0,036642616	0,909620783	0,970851175	0,908651948	1,339077988
N-palmitoyl-sphingosine (d18:1/16:0)	HMDB04949	Lipid	Sphingolipid Metabolism	0,277470196	0,305706397	0,386158417	0,966191938	0,875390909	0,073480605
N1-Methyl-2-pyridone-5-carboxamide	HMDB04193	Cofactors and Vitamins	Nicotinate and Nicotinamide Metabolism	0,293519307	-0,451844691	-2,490996004	-1,089880817	-1,999665885	-0,632397318
N1-methyladenosine	HMDB03331	Nucleotide	Purine Metabolism, Adenine containing	-0,526859124	0,119447886	-0,148448367	-1,187441943	-1,619766069	-0,083250815

N6,N6,N6-trimethyllysine	HMDB01325	Amino Acid	Lysine Metabolism	-0,051389671	1,333147349	0,222901323	-1,08909691	-0,887493284	-0,36509411
N6-succinyladenosine	HMDB00912	Nucleotide	Purine Metabolism, Adenine containing	-1,74041371	0,292953591	-0,619704303	-1,749559477	-1,741462108	0,241837869
nicotinamide	HMDB01406	Cofactors and Vitamins	Nicotinate and Nicotinamide Metabolism	-1,693197615	-1,798852408	-1,716741444	-2,6751639	-0,855755861	-1,208251176
nonadecanoate (19:0)	HMDB00772	Lipid	Long Chain Fatty Acid	1,107494971	1,415788751	2,955639178	1,590405486	1,692414296	1,508741108
O-methylcatechol sulfate		Xenobiotics	Benzoate Metabolism	-0,99004067	-0,140705497	-0,08870079	-0,207751465	-1,199720757	-0,216161522
O-sulfo-L-tyrosine		Xenobiotics	Chemical	-1,147072546	0,948383157	0,546407147	-0,530349014	-2,056046479	-0,319558027
octanoylcarnitine	HMDB00791	Lipid	Fatty Acid Metabolism(Acyl Carnitine)	0,487229331	0,615184056	0,031268355	0,890082132	-0,063633548	0,576510787
oleate/vaccenate (18:1)		Lipid	Long Chain Fatty Acid	0,670020559	1,171703753	1,201138427	0,754863695	1,071790879	0,376503049
oleoyl ethanolamide	HMDB02088	Lipid	Endocannabinoid	1,60987986	2,060156586	1,397832737	1,329031223	2,483124719	1,648059452
oleoylcarnitine	HMDB05065	Lipid	Fatty Acid Metabolism(Acyl Carnitine)	0,760531339	1,598116854	1,185478882	0,888550417	1,94761066	1,325616191
ornithine	HMDB03374	Amino Acid	Urea cycle; Arginine and Proline Metabolism	0,763917617	0,042060508	0,531484385	-0,289199441	0,80565522	0,533866781
orotate	HMDB00226	Nucleotide	Pyrimidine Metabolism, Orotate containing	-1,325897879	-0,072310663	-0,814446155	-0,485313766	-0,368581415	-0,950112459
oxalate (ethanedioate)	HMDB02329	Cofactors and Vitamins	Ascorbate and Aldarate Metabolism	-1,031529868	-0,603048917	0,30924751	-0,485035284	-2,985254062	-0,097288497
p-cresol sulfate	HMDB11635	Amino Acid	Phenylalanine and Tyrosine Metabolism	0,571000822	-0,0282366	0,516417872	0,199780594	-0,159922413	0,095767186
p-cresol-glucuronide*	HMDB11686	Amino Acid	Phenylalanine and Tyrosine Metabolism	0,123599964	-1,129584128	-0,017522088	-0,411202891	-0,151154498	-0,402201339
palmitate (16:0)	HMDB00220	Lipid	Long Chain Fatty Acid	0,914350412	0,831154693	1,350581216	0,925841509	1,239473489	0,693789355
palmitoleate (16:1n7)	HMDB03229	Lipid	Long Chain Fatty Acid	0,244142204	0,823944911	1,198267514	0,11090929	1,099094939	0,378687128
palmitoyl ethanolamide	HMDB02100	Lipid	Endocannabinoid	0,505295641	0,139642531	-0,144783191	-0,213710619	0,981983823	0,882951001
palmitoyl sphingomyelin (d18:1/16:0)		Lipid	Sphingolipid Metabolism	0,214927725	0,560262115	1,6198307	0,638205236	0,777474992	1,306479601
palmitoylcarnitine	HMDB00222	Lipid	Fatty Acid Metabolism(Acyl Carnitine)	0,450847633	0,173205782	1,456785516	0,667129457	1,150502614	1,679969912
pantothenate	HMDB00210	Cofactors and Vitamins	Pantothenate and CoA Metabolism	0,12950726	0,271992531	-1,255842391	-1,075878304	-1,461902213	0,304649246
phenol sulfate	HMDB60015	Amino Acid	Phenylalanine and Tyrosine Metabolism	0,203243861	1,329632001	0,599823743	0,155401186	-0,464754523	-0,683183029
phenylacetylcarnitine		Peptide	Acetylated Peptides	-0,591438248	-0,73867218	-0,428012531	-0,89163324	0,01913876	0,058479389
phenylacetylglutamine	HMDB06344	Peptide	Acetylated Peptides	0,106105408	-0,15617057	0,328599512	-0,824785937	-0,909840282	-0,749320855
phenylalanine	HMDB00159	Amino Acid	Phenylalanine and Tyrosine Metabolism	-1,117506721	-0,958052583	-1,474073123	-1,701605144	-0,589725927	-1,540710683
phenylalanylphenylalanine		Peptide	Dipeptide	0,144316764	0,668178867	-0,017416058	-0,39508622	-0,737407105	-0,494370673
phenylalanyltryptophan		Peptide	Dipeptide	-0,301698369	0,837473089	0,710181795	0,128611695	-0,646231721	-0,547880313
phenyllactate (PLA)	HMDB00779	Amino Acid	Phenylalanine and Tyrosine Metabolism	-0,205417353	-0,425345894	0,202557488	-0,650997805	-0,414736469	-1,312869707
phenylpyruvate	HMDB00205	Amino Acid	Phenylalanine and Tyrosine Metabolism	-0,808321183	0,110392916	0,339451271	-0,704083086	-2,816245461	-1,257551394

phosphate	HMDB01429	Energy	Oxidative Phosphorylation	0,037852294	0,579074132	-0,454337672	0,216905057	-0,437533985	0,722670571
phosphatidylcholine (16:0/22:5n3, 18:1/20:4)*		Lipid	Phospholipid Metabolism	-1,594815986	-1,219495522	-0,056241227	-1,10084843	-0,817351066	0,31179458
phosphatidylcholine (18:0/20:5, 16:0/22:5n6)*		Lipid	Phospholipid Metabolism	-4,675936536	-4,660562295	-4,674937417	-4,667463181	-4,675881256	0,805414895
phosphoethanolamine	HMDB00224	Lipid	Phospholipid Metabolism	-0,174431284	-0,15961049	0,055169236	-0,245777356	-0,096098373	-0,101388162
picolinate	HMDB02243	Amino Acid	Tryptophan Metabolism	-0,417010342	0,612082477	-0,123983633	-0,349174467	-0,403875999	-0,224789933
pipecolate	HMDB00070	Amino Acid	Lysine Metabolism	-0,570846756	0,386176972	-1,031858469	-0,023275615	-0,353178016	-0,188952764
piperine	HMDB29377	Xenobiotics	Food Component/Plant	0,364123276	-1,215020976	-1,216138436	0,025807695	-0,040981689	-0,153694023
pregn steroid monosulfate*		Lipid	Steroid	1,452863107	-0,427118394	-0,300258752	0,560580278	0,78722516	-0,850071085
pregnen-diol disulfate*		Lipid	Steroid	0,916785032	-1,047794627	-0,26665515	0,50339539	0,525749843	-0,675714
pregnenolone sulfate	HMDB00774	Lipid	Steroid	0,706556453	-1,239144737	-1,432403316	-0,1899361	-0,375634438	-1,53462473
pro-hydroxy-pro	HMDB06695	Amino Acid	Urea cycle; Arginine and Proline Metabolism	-0,938111721	0,139961835	-0,432220093	-0,94881897	-2,133953578	0,07878444
proline	HMDB00162	Amino Acid	Urea cycle; Arginine and Proline Metabolism	-0,353775202	-1,094359646	-0,750114614	-0,060361577	0,61209404	-0,312206084
prolylserine		Peptide	Dipeptide	-1,178675391	0,559867805	-0,874877452	-0,153175636	-1,747334103	0,181717365
propionylcarnitine	HMDB00824	Lipid	Fatty Acid Metabolism (also BCAA Metabolism)	-1,702301462	-1,472348769	-1,83305076	-1,509273321	-2,445207748	-1,812254564
propionylglycine	HMDB00783	Lipid	Fatty Acid Metabolism (also BCAA Metabolism)	-1,431064683	0,642749031	0,211558044	0,03576049	-1,432853646	0,297492748
propyl 4-hydroxybenzoate sulfate		Xenobiotics	Benzoate Metabolism	-0,28004435	-0,160030087	-0,964389864	-0,965206212	-0,964104663	-0,719314384
pseudouridine	HMDB00767	Nucleotide	Pyrimidine Metabolism, Uracil containing	-0,830429814	-0,608182334	-0,546964657	-1,656356029	-1,944695155	-0,75817095
pyridoxate	HMDB00017	Cofactors and Vitamins	Vitamin B6 Metabolism	-1,293093961	-0,792050515	-0,890203044	-0,98387694	-0,992979706	-0,067302854
pyroglutamine*		Amino Acid	Glutamate Metabolism	1,87957793	-0,626673439	0,610947992	-0,689650513	-0,593264183	-0,565653917
pyroglutamylvaline		Peptide	Dipeptide	-1,76397267	0,058852234	0,359884611	-1,756744709	-1,138847494	-1,459755521
pyrraline		Xenobiotics	Food Component/Plant	-1,567629756	1,032781248	-1,565713682	-1,566292278	-1,569672417	-0,023068222
pyruvate	HMDB00243	Carbohydrate	Glycolysis, Gluconeogenesis, and Pyruvate Metabolism	1,077173159	-0,461299176	-1,145160374	-0,856926372	1,237075393	0,970796756
quinolinate	HMDB00232	Cofactors and Vitamins	Nicotinate and Nicotinamide Metabolism	-0,869969283	-0,150213137	-1,052713934	-0,95547704	-2,350144302	-1,061000131
retinol (Vitamin A)	HMDB00305	Cofactors and Vitamins	Vitamin A Metabolism	-0,39759363	-0,477203482	-0,103049275	-0,821483339	-0,350918301	0,298507505
S-allylcysteine		Xenobiotics	Food Component/Plant	-0,770417631	-0,766187719	-0,76565589	-0,771124751	-0,76942821	-0,770746875
S-methylcysteine	HMDB02108	Amino Acid	Methionine, Cysteine, SAM and Taurine Metabolism	0,305004515	-0,283340104	-0,389578116	-0,209005543	-0,18103488	-0,327628709
salicylate	HMDB01895	Xenobiotics	Drug	-0,392333441	0,21342665	1,353215273	-0,63120177	-0,479199494	-0,401581886
sarcosine	HMDB00271	Amino Acid	Glycine, Serine and Threonine Metabolism	-2,985118643	-2,01677409	-1,63829763	-1,350384692	-0,971382089	-2,410337384
sebacate (decanedioate)	HMDB00792	Lipid	Fatty Acid, Dicarboxylate	-1,817384648	-1,032780203	0,151644592	0,94137152	-1,572776832	-1,243592908

serine	HMDB00187	Amino Acid	Glycine, Serine and Threonine Metabolism	-0,143549025	0,370352714	0,762181526	0,907240331	1,703911886	0,678599337
spermidine	HMDB01257	Amino Acid	Polyamine Metabolism	-1,685396907	-1,111632982	-0,342848631	-1,98221695	-0,552625192	-0,972073595
sphinganine	HMDB00269	Lipid	Sphingolipid Metabolism	-0,451577496	-0,146874921	0,223283252	-0,313149373	3,98E-05	-0,086725086
sphinganine-1-phosphate	HMDB01383	Lipid	Sphingolipid Metabolism	0,534509068	0,154663303	-0,025445444	0,067298495	0,540480629	0,558829032
sphingomyelin (d18:1/18:1, d18:2/18:0)		Lipid	Sphingolipid Metabolism	0,811278853	-0,229796777	0,615922777	-0,259839701	1,019925512	0,894671389
sphingomyelin (d18:2/14:0, d18:1/14:1)*		Lipid	Sphingolipid Metabolism	-0,428669498	-1,576085898	0,412411969	-0,767128847	-0,0762335	0,237358341
sphingomyelin (d18:2/16:0, d18:1/16:1)*		Lipid	Sphingolipid Metabolism	0,085979879	-0,182334082	0,505928351	0,213650584	0,655503542	0,155957227
sphingosine	HMDB00252	Lipid	Sphingolipid Metabolism	-0,495731489	-0,196984058	-0,771343656	-0,885126689	0,253371305	-0,6243803
sphingosine 1-phosphate	HMDB00277	Lipid	Sphingolipid Metabolism	0,628920239	0,509390015	-0,2140198	-0,037715162	0,73586825	0,979090877
stachydrine	HMDB04827	Xenobiotics	Food Component/Plant	0,228747497	-0,170136364	0,140147382	0,329379384	-0,38864001	1,394356568
stearate (18:0)	HMDB00827	Lipid	Long Chain Fatty Acid	1,147809345	1,455707544	2,007861567	0,95552601	1,169372505	0,751164568
stearidonate (18:4n3)	HMDB06547	Lipid	Polyunsaturated Fatty Acid (n3 and n6)	-0,407276932	-0,2483526	0,243889535	-0,856890143	0,123157811	-0,278255327
stearoylcarnitine	HMDB00848	Lipid	Fatty Acid Metabolism(Acyl Carnitine)	0,818928235	0,910766723	1,964246579	1,268308226	1,424888913	1,935233327
suberate (octanedioate)	HMDB00893	Lipid	Fatty Acid, Dicarboxylate	-1,45260714	-1,460683198	0,122395658	0,663565928	0,366991676	-1,451776087
succinate	HMDB00254	Energy	TCA Cycle	0,399037147	0,299508163	0,171215326	0,018594558	0,492782227	0,902394732
succinimide		Xenobiotics	Chemical	0,582202292	0,512786982	1,410642564	0,985845652	1,26627125	0,549541445
succinylcarnitine		Energy	TCA Cycle	-1,656163297	-1,624204044	-2,046233084	-1,698889765	-2,242435481	-2,200461504
sucrose	HMDB00258	Carbohydrate	Disaccharides and Oligosaccharides	-1,27939743	-1,274848508	-1,279397888	-1,275687937	-1,278992028	-1,274918055
sulfate*	HMDB01448	Xenobiotics	Chemical	-1,874510917	-0,553027334	-1,985816315	-1,323448448	-0,751969188	0,100253332
tartarate	HMDB00956	Xenobiotics	Food Component/Plant	-1,336799332	-0,170345709	-0,335847179	0,015192139	-1,330764128	0,0038063
tartronate (hydroxymalonate)	HMDB35227	Xenobiotics	Bacterial/Fungal	-2,048792541	0,212633415	-0,084783188	0,814247611	-3,394003708	0,751568622
taurine	HMDB00251	Amino Acid	Methionine, Cysteine, SAM and Taurine Metabolism	-1,422298068	-0,605126159	-0,486322068	-0,285823856	-0,131288722	-0,25126238
tauro-beta-muricholate	HMDB00932	Lipid	Primary Bile Acid Metabolism	0,634472003	0,565039506	1,394488897	0,494089847	0,13303049	0,37410025
taurochenodeoxycholate	HMDB00951	Lipid	Primary Bile Acid Metabolism	-1,329561259	-0,37279587	-0,949111365	-0,362532878	-2,327065073	0,023396118
taurocholate	HMDB00036	Lipid	Primary Bile Acid Metabolism	-1,396492139	-0,26250217	-0,529404794	0,011794873	-1,848198702	0,174224697
taurocholenate sulfate		Lipid	Secondary Bile Acid Metabolism	0,762868577	0,291963171	-0,67455346	0,354208466	-1,284227431	-0,285643799
taurolithocholate 3-sulfate	HMDB02580	Lipid	Secondary Bile Acid Metabolism	0,95486278	-0,857481793	0,34149829	0,607833865	-0,482686527	-0,323684094
tauroursodeoxycholate	HMDB00874	Lipid	Secondary Bile Acid Metabolism	-0,984786497	0,352299056	-0,98161418	-0,108421318	-0,983316668	-0,987214415
theobromine	HMDB02825	Xenobiotics	Xanthine Metabolism	0,493668693	0,475788756	-0,221612596	-1,190754861	0,503925206	-1,189891481
threonate	HMDB00943	Cofactors and Vitamins	Ascorbate and Aldarate Metabolism	0,496712878	-0,195566623	1,213363738	0,019740529	-1,350766423	0,266320793

threonine	HMDB00167	Amino Acid	Glycine, Serine and Threonine Metabolism	0,110042623	-0,370344412	-0,457475442	0,274025981	0,709314454	-1,078045084
thymol sulfate	HMDB01878	Xenobiotics	Food Component/Plant	-0,14670438	1,681143736	-1,237964848	-1,238811905	-1,241362974	-1,239895943
thyroxine	HMDB01918	Amino Acid	Phenylalanine and Tyrosine Metabolism	-0,369402213	0,644055108	-0,393705161	0,31876433	-0,335733843	-0,441586133
tiglylcarnitine	HMDB02366	Amino Acid	Leucine, Isoleucine and Valine Metabolism	-0,403567715	-0,233737778	-0,382186653	-0,391322282	-0,364987002	-0,304203679
trans-4-hydroxyproline	HMDB00725	Amino Acid	Urea cycle; Arginine and Proline Metabolism	-0,377665836	-0,322797904	-0,658349551	-0,480371986	-0,331333187	0,168840543
trigonelline (N'-methylnicotinate)	HMDB00875	Cofactors and Vitamins	Nicotinate and Nicotinamide Metabolism	-1,510081849	0,059842112	0,618377033	-0,782312044	-0,974643835	0,141294437
trimethylamine N-oxide	HMDB00925	Lipid	Phospholipid Metabolism	0,04723743	-0,222176381	-0,015646459	-1,022567343	0,179845465	0,435492259
tryptophan	HMDB00929	Amino Acid	Tryptophan Metabolism	-0,628915056	-0,278810639	-0,674782768	-0,997440523	-1,374873543	-0,689694938
tryptophan betaine	HMDB61115	Amino Acid	Tryptophan Metabolism	-1,811028966	-0,708423775	-0,865064712	-0,139047025	0,122501825	-0,953536472
tyrosine	HMDB00158	Amino Acid	Phenylalanine and Tyrosine Metabolism	-1,995026155	-0,950012035	-1,221970396	-1,983127514	-0,471174538	-1,168539269
urate	HMDB00289	Nucleotide	Purine Metabolism, (Hypo)Xanthine/Inosine containing	0,53955642	0,062260135	-0,031639115	0,141313674	0,77966851	-0,175154896
urea	HMDB00294	Amino Acid	Urea cycle; Arginine and Proline Metabolism	-1,066760653	0,858666168	-0,929156187	-1,143235566	-1,463134804	-0,652026053
uridine	HMDB00296	Nucleotide	Pyrimidine Metabolism, Uracil containing	0,856068583	0,37499504	0,638165685	-0,270677037	0,364764376	0,387706559
ursodeoxycholate	HMDB00946	Lipid	Secondary Bile Acid Metabolism	-1,574947081	-0,101573148	0,745874364	1,027055258	-1,57348428	-0,252152872
valine	HMDB00883	Amino Acid	Leucine, Isoleucine and Valine Metabolism	0,846231854	1,095870561	0,399640987	0,164260482	0,681435852	0,05899951
valylglycine	HMDB29127	Peptide	Dipeptide	-0,96459597	0,678865783	-0,240264809	-0,498331534	-1,921641014	0,388437916
vanillic alcohol sulfate		Amino Acid	Phenylalanine and Tyrosine Metabolism	-0,929173464	-0,926921251	-0,928220762	-0,929157995	-0,928397477	-0,928154776
vanillylmandelate (VMA)	HMDB00291	Amino Acid	Phenylalanine and Tyrosine Metabolism	-1,641749184	-0,219359842	-0,299196484	-0,42275828	-2,76779117	-0,483560392
xanthine	HMDB00292	Nucleotide	Purine Metabolism, (Hypo)Xanthine/Inosine containing	1,035865238	1,516641506	0,036326565	0,239621258	1,512721391	1,232499603
glycochenodeoxycholate glucuronide (1)		Lipid	Primary Bile Acid Metabolism	0,996139908	-1,622136795	0,393301814	0,035884227	-1,623861638	-1,622923538
suberoylcarnitine		Lipid	Fatty Acid Metabolism(Acyl Carnitine)	-0,077183187	-0,373154028	0,879270539	0,071314228	0,629496341	0,631398261
glycochenodeoxycholate sulfate		Lipid	Primary Bile Acid Metabolism	-0,662277505	0,15631895	-0,173752664	-2,530218966	-2,523907987	0,042894119
3-hydroxybutyrylcarnitine (2)		Lipid	Fatty Acid Metabolism(Acyl Carnitine)	-0,650849935	0,062146048	0,330795889	-0,227243021	-0,269324144	-0,487233106
6-hydroxyindole sulfate		Xenobiotics	Chemical	0,043570486	-0,062968334	1,041496123	0,032587291	-0,970405799	-0,501722832
lactosyl-N-palmitoyl-sphingosine (d18:1/16:0)		Lipid	Sphingolipid Metabolism	0,084321629	0,357243669	0,61104381	0,844809473	-0,190641024	-0,569001422
1,2,3-benzenetriol sulfate (2)		Xenobiotics	Chemical	-1,319602661	0,73675803	0,150412246	-0,812108351	0,435152087	0,390793101
acisoga		Amino Acid	Polyamine Metabolism	-0,199726625	-0,294664288	-0,064210683	-0,667930539	-0,435530766	-0,494247994

hexadecanedioate	HMDB00672	Lipid	Fatty Acid, Dicarboxylate	0,422524775	-0,545410723	0,83358546	1,576918745	0,264853637	0,449498291
hexanoylglycine	HMDB00701	Lipid	Fatty Acid Metabolism(Acyl Glycine)	1,237023347	0,787020567	1,596127355	0,814856105	1,671683716	1,3252315
tetradecanedioate	HMDB00872	Lipid	Fatty Acid, Dicarboxylate	0,170852045	-1,502162292	0,155101606	1,22446876	-0,575158167	-0,809551237
tyramine O-sulfate	HMDB06409	Amino Acid	Phenylalanine and Tyrosine Metabolism	-1,428297442	-1,4315207	-1,433188062	-1,432632307	-1,433340664	-1,431487073
N-methylpipecolate		Xenobiotics	Chemical	-0,904738241	-0,348315797	0,176773668	0,012107825	-1,99275245	-0,130168527
2-aminooctanoate	HMDB00991	Lipid	Fatty Acid, Amino	0,831841817	1,312363888	0,137037145	0,027651133	-0,803002712	0,185146256
trans-urocanate	HMDB00301	Amino Acid	Histidine Metabolism	-4,119498716	0,730692393	-0,717700647	-1,29644984	-1,47167325	-0,602688239
3-methylhistidine	HMDB00479	Amino Acid	Histidine Metabolism	-0,508786479	1,700001463	-0,239716831	-0,796611976	-0,596717069	-0,48529587
9,10-DiHOME	HMDB04704	Lipid	Fatty Acid, Dihydroxy	-0,169392262	2,341976066	0,196547757	0,317564604	-1,066988839	2,914895287
N-acetylglutamate	HMDB01138	Amino Acid	Glutamate Metabolism	-1,575587342	0,478650389	-0,113615545	-0,682958323	-0,744515033	-0,295253748
octadecanedioate	HMDB00782	Lipid	Fatty Acid, Dicarboxylate	0,45955719	0,587707763	1,746102538	1,190827061	0,474470425	0,48057277
oleamide	HMDB02117	Lipid	Fatty Acid, Amide	0,760238155	0,897771464	0,190369947	0,948673986	0,325670846	0,650641396
docosadioate		Lipid	Fatty Acid, Dicarboxylate	-0,784108431	-0,02740959	0,15706938	-0,105076385	-0,938219819	-0,641418326
pyridoxal	HMDB01545	Cofactors and Vitamins	Vitamin B6 Metabolism	-1,722725047	-0,254874349	-1,272248207	-1,213752901	-1,719048769	0,105919059
linoleamide (18:2n6)		Lipid	Fatty Acid, Amide	0,687573365	0,717484373	0,269005842	0,735286032	0,353822015	0,61677596
1-oleoylglycerol (18:1)	HMDB11567	Lipid	Monoacylglycerol	-0,09426255	-0,488102038	1,261734906	0,02595629	-0,045618697	-0,266183297
N4-acetylcytidine	HMDB05923	Nucleotide	Pyrimidine Metabolism, Cytidine containing	-0,429640378	-0,958629373	0,575249994	-2,259155181	-2,256792611	-1,0767995
1-palmitoylglycerol (16:0)	HMDB31074	Lipid	Monoacylglycerol	-0,43804497	-0,612188601	0,447580301	-0,214031967	-0,594195135	-0,440570211
1-(1-enyl-stearoyl)-2-docosahexaenoyl-GPC (P-18:0/22:6)*		Lipid	Plasmalogen	-0,976498641	0,104158455	-0,050526198	-0,761869162	-0,160555505	0,520252581
isobutyrylglycine	HMDB00730	Amino Acid	Leucine, Isoleucine and Valine Metabolism	-1,082876461	0,641302411	-1,079290692	-1,075799705	0,117961953	-1,07562156
carnosine	HMDB00033	Peptide	Dipeptide Derivative	-1,128252683	-1,125396025	-1,127829192	-1,126511589	-1,125068088	-1,125434101
cystine	HMDB00192	Amino Acid	Methionine, Cysteine, SAM and Taurine Metabolism	2,361369302	1,687633295	1,227633558	1,600949087	2,447704158	1,291767407
dodecanedioate	HMDB00623	Lipid	Fatty Acid, Dicarboxylate	-0,268821688	-1,387820849	-0,05173304	1,07933034	-1,343971827	-0,80218999
erucate (22:1n9)	HMDB02068	Lipid	Long Chain Fatty Acid	-0,031559413	1,813391019	0,929512616	0,70943485	0,839557368	0,816389223
malate	HMDB00156	Energy	TCA Cycle	0,549142453	-0,241975696	0,433218544	0,046616461	0,578965052	0,837032883
malonate	HMDB00691	Lipid	Fatty Acid Synthesis	0,072590448	0,239408232	1,94790123	0,958036744	0,053402139	0,535975333
maltose	HMDB00163	Carbohydrate	Glycogen Metabolism	-1,379859808	-1,37608262	-1,377105187	-1,37481084	-1,379082695	-1,379472797
2-aminoadipate	HMDB00510	Amino Acid	Lysine Metabolism	-0,45239938	0,186648769	-0,207422021	-0,101359916	0,027014255	-0,476485274
2-pyrrolidinone	HMDB02039	Xenobiotics	Chemical	0,487738385	-0,235910142	1,463940203	-0,584491865	0,353628435	0,134257976
aconitate [cis or trans]	HMDB00072	Energy	TCA Cycle	0,350310688	0,74236935	1,271006014	1,121890148	0,72840028	1,075517529

homostachydrine*	HMDB33433	Xenobiotics	Food Component/Plant	-1,672477842	0,574051559	1,340210105	0,696620758	-1,670938401	0,889653288
phosphatidylcholine (14:0/14:0, 16:0/12:0)		Lipid	Phospholipid Metabolism	-0,790548747	-1,695053528	-0,408884476	-0,218363697	-0,372864034	-0,044296559
1-(1-enyl-palmitoyl)-2-arachidonoyl-GPE (P-16:0/20:4)*		Lipid	Plasmalogen	-1,032638102	-0,151296128	-0,555896185	-0,593982255	-0,931779444	0,109675369
1-(1-enyl-palmitoyl)-2-linoleoyl-GPE (P-16:0/18:2)*		Lipid	Plasmalogen	-0,529596403	0,454570467	0,325893987	-0,111247115	-0,2466269	0,439791057
1-(1-enyl-palmitoyl)-2-oleoyl-GPC (P-16:0/18:1)*		Lipid	Plasmalogen	-0,880446679	0,417539333	1,05518415	-0,381459778	0,783590877	1,255854625
1-(1-enyl-palmitoyl)-2-palmitoleoyl-GPC (P-16:0/16:1)*		Lipid	Plasmalogen	-1,715697483	0,079844819	0,04233339	-1,528862571	-0,458435097	-0,198277125
1-(1-enyl-stearoyl)-2-arachidonoyl-GPC (P-18:0/20:4)		Lipid	Plasmalogen	-0,007864263	0,513472892	0,541623803	-0,253762592	0,939559602	1,139332189
1-(1-enyl-stearoyl)-2-oleoyl-GPC (P-18:0/18:1)		Lipid	Plasmalogen	-0,701090562	1,181770305	1,484059686	0,35516881	0,670904792	1,226329365
1-(1-enyl-stearoyl)-2-oleoyl-GPE (P-18:0/18:1)		Lipid	Plasmalogen	-1,114100835	1,014514953	0,424348563	0,046182214	-0,335725805	0,953057134
1-arachidoyl-GPC (20:0)	HMDB10390	Lipid	Lysolipid	-2,38829206	0,093206057	0,232693217	-0,222704297	-0,6816855	0,056096622
1-lignoceroyl-GPC (24:0)		Lipid	Lysolipid	-2,683072026	-0,734052213	-2,683019985	-2,687016058	-0,800245626	0,219276638
1-margaroyl-2-linoleoyl-GPC (17:0/18:2)*		Lipid	Phospholipid Metabolism	-0,398578247	-0,808736158	0,888365913	0,28496175	-0,775433477	-0,048983681
1-margaroyl-2-oleoyl-GPC (17:0/18:1)*		Lipid	Phospholipid Metabolism	-0,994006551	-0,39387486	0,243095334	-0,068800502	-0,544368823	1,226238751
1-myristoyl-2-linoleoyl-GPC (14:0/18:2)*		Lipid	Phospholipid Metabolism	-0,680418276	-1,442105807	0,152361208	-1,278621459	-1,313153564	-1,193759701
1-myristoyl-2-palmitoyl-GPC (14:0/16:0)		Lipid	Phospholipid Metabolism	-0,654088115	-1,569182267	-0,010145852	-0,465091356	-0,230409477	0,452979407
1-oleoyl-2-docosahexaenoyl-GPC (18:1/22:6)*		Lipid	Phospholipid Metabolism	-2,228735474	-1,093680114	-0,466534796	-1,272545514	-2,172907106	-0,588955904
1-palmitoyl-2-linoleoyl-GPE (16:0/18:2)	HMDB05322	Lipid	Phospholipid Metabolism	-0,624992631	-1,67566931	-0,771663274	-0,280568475	-1,067390912	-1,781452458
1-palmitoyl-2-oleoyl-GPC (16:0/18:1)		Lipid	Phospholipid Metabolism	-1,31012786	-0,222825491	-0,586417638	-0,332204717	-0,945714931	0,700250397
1-palmitoyl-2-palmitoleoyl-GPC (16:0/16:1)*		Lipid	Phospholipid Metabolism	-1,618624537	-1,100886694	-1,00238739	-1,296568734	-1,395459624	-0,626668487
1-palmityl-2-oleoyl-GPC (O-16:0/18:1)*		Lipid	Phospholipid Metabolism	-0,576170165	0,504455059	-0,166977733	0,162038864	0,045122597	-0,224545243
1-pentadecanoyl-2-linoleoyl-GPC (15:0/18:2)*		Lipid	Phospholipid Metabolism	0,836943919	-0,136407244	1,781115217	0,509710381	0,156375068	0,536245605
1-stearoyl-2-docosahexaenoyl-GPE (18:0/22:6)*	HMDB05334	Lipid	Phospholipid Metabolism	-2,109382933	-1,891562584	-1,217539868	-1,472411301	-1,200571517	-0,966545522
1-stearoyl-2-docosapentaenoyl-GPC (18:0/22:5n3)*		Lipid	Phospholipid Metabolism	-2,146230034	-2,153263291	-0,458313759	-1,649540255	-2,170963578	-0,737776838
1-stearoyl-2-docosapentaenoyl-GPC (18:0/22:5n6)*		Lipid	Phospholipid Metabolism	-1,48167657	-0,907907006	-1,383029693	-1,800632707	-1,125607248	-0,443538993
1-stearoyl-2-oleoyl-GPE (18:0/18:1)		Lipid	Phospholipid Metabolism	-1,617090648	-0,343116524	-0,764724019	-0,124752213	-1,581127475	-1,086971542
adenosine 3',5'-cyclic monophosphate (cAMP)	HMDB00058	Nucleotide	Purine Metabolism, Adenine containing	-0,467886582	1,257212856	0,643463349	-0,791502583	-0,76757816	1,124201667
palmitoyl dihydrosphingomyelin (d18:0/16:0)*		Lipid	Sphingolipid Metabolism	-0,965542972	0,098080549	0,329938028	-0,101899958	-0,465924811	1,030008496
prolylglycine		Peptide	Dipeptide	-1,191980685	-0,120217341	-0,116697622	-1,056082209	-1,465232643	-0,57371268

sphingomyelin (d18:1/14:0, d16:1/16:0)*		Lipid	Sphingolipid Metabolism	0,386235112	-0,133577235	1,627901546	0,21523444	0,72715736	1,333990809
sphingomyelin (d18:1/15:0, d16:1/17:0)*		Lipid	Sphingolipid Metabolism	0,946466531	0,016778108	1,918438696	0,563665897	1,357303742	1,633438865
sphingomyelin (d18:1/17:0, d17:1/18:0, d19:1/16:0)		Lipid	Sphingolipid Metabolism	0,663747403	-0,479784614	1,076912317	0,100064264	0,861072692	1,39641459
sphingomyelin (d18:1/20:0, d16:1/22:0)*		Lipid	Sphingolipid Metabolism	0,356431503	0,30873139	0,967379581	-0,161011747	0,291284696	0,824610127
sphingomyelin (d18:1/20:1, d18:2/20:0)*		Lipid	Sphingolipid Metabolism	0,333536447	0,004748704	-0,533486001	-0,989873677	0,476510381	-0,176812233
sphingomyelin (d18:1/24:1, d18:2/24:0)*		Lipid	Sphingolipid Metabolism	-1,232559746	-0,957728476	-1,44862934	-1,241024929	-1,306295169	-0,629263941
sphingomyelin (d18:2/23:0, d18:1/23:1, d17:1/24:1)*		Lipid	Sphingolipid Metabolism	0,358198787	-0,358573705	1,500502173	0,228982137	1,262756645	2,346031162
sphingomyelin (d18:2/24:1, d18:1/24:2)*		Lipid	Sphingolipid Metabolism	-1,208053641	-2,006972544	-2,140424369	-1,928222651	-1,167147288	-1,464776013
stearoyl sphingomyelin (d18:1/18:0)	HMDB01348	Lipid	Sphingolipid Metabolism	0,715215639	-0,355426517	0,32140061	-0,57434204	0,586855699	0,682688306
palmitoylcholine		Lipid	Fatty Acid Metabolism (Acyl Choline)	-0,520532537	0,073814777	-0,224601751	-0,727360673	-0,046959385	0,933271811
N-acetylcitrulline	HMDB00856	Amino Acid	Urea cycle; Arginine and Proline Metabolism	-1,116037673	-1,117821099	-0,363795646	-1,114315995	-1,116079742	-0,484231308
S-adenosylhomocysteine (SAH)	HMDB00939	Amino Acid	Methionine, Cysteine, SAM and Taurine Metabolism	-0,430742853	-1,670648147	-0,434164387	-1,671287286	0,055634998	-1,671812895
1-palmitoyl-2-stearoyl-GPC (16:0/18:0)		Lipid	Phospholipid Metabolism	-1,25004243	-1,45476724	-0,150981895	-0,785040526	-1,029147161	-0,003073711
1-stearoyl-2-dihomo-linolenoyl-GPC (18:0/20:3n3 or 6)*		Lipid	Phospholipid Metabolism	-1,919264791	-1,846637862	-1,547784494	-1,985417879	-2,591546458	-1,092934212
1-stearoyl-2-oleoyl-GPC (18:0/18:1)		Lipid	Phospholipid Metabolism	-2,014244673	-0,002890963	-0,431377574	-0,225311926	-1,183890575	0,572887626
behenoyl sphingomyelin (d18:1/22:0)*		Lipid	Sphingolipid Metabolism	0,397895702	0,724127202	1,155508362	0,517656085	0,069898635	0,945947753
sphingomyelin (d18:1/21:0, d17:1/22:0, d16:1/23:0)*		Lipid	Sphingolipid Metabolism	0,961407539	0,258199629	2,350575376	0,505457446	1,421920855	2,59570697
sphingomyelin (d18:1/22:1, d18:2/22:0, d16:1/24:1)*		Lipid	Sphingolipid Metabolism	0,26308043	-0,206586316	0,308255243	-0,267795712	0,326911789	0,317363506
1-stearoyl-2-meadoyl-GPC (18:0/20:3n9)*		Lipid	Phospholipid Metabolism	-1,334910865	-0,633090815	-0,673047844	-0,825971304	-0,748924338	0,293059661
1-methylurate	HMDB03099	Xenobiotics	Xanthine Metabolism	0,599218028	-0,311927078	-0,174256248	-1,937957802	-0,546652489	-0,282128396
7-methylurate		Xenobiotics	Xanthine Metabolism	0,023778142	0,485282699	0,131842943	-1,535885561	0,552688141	-1,535121937
2-hydroxyhippurate (salicylurate)	HMDB00840	Xenobiotics	Benzoate Metabolism	-0,733621718	-0,177690389	0,945884637	-1,309502372	-1,332051293	-0,871173662
2-isopropylmalate	HMDB00402	Xenobiotics	Food Component/Plant	-1,001409289	-0,016270302	0,274049162	0,471578267	-0,998885202	0,151439051
2-linoleoylglycerol (18:2)	HMDB11538	Lipid	Monoacylglycerol	0,380112962	-0,656050368	1,190844485	-0,096331361	-0,014986848	-0,138100815
hydantoin-5-propionic acid	HMDB01212	Amino Acid	Histidine Metabolism	1,40809111	0,658173862	0,819014075	0,624941869	0,792317072	0,157771936
N6-acetyllysine	HMDB00206	Amino Acid	Lysine Metabolism	-1,50095669	0,164262957	0,056025631	-0,909317122	-1,051352867	0,20770819
quinate	HMDB03072	Xenobiotics	Food Component/Plant	-1,144939979	-0,515223954	-0,154562955	0,013048717	-1,143719872	-0,347718433
ribitol	HMDB00508	Carbohydrate	Pentose Metabolism	-0,67678876	0,209621832	-2,184920645	0,90330315	-0,090201068	-0,136775792
4-vinylguaiacol sulfate		Xenobiotics	Food Component/Plant	-0,687362337	-0,683987279	-0,689659955	-0,685973376	-0,688431301	-0,682862602

galactonate	HMDB00565	Carbohydrate	Fructose, Mannose and Galactose Metabolism	-0,922646783	-1,245979031	-1,415525112	-1,23242041	-1,421106384	0,192349084
orotidine	HMDB00788	Nucleotide	Pyrimidine Metabolism, Orotate containing	-0,253615916	0,264088047	-0,017497609	-0,185353363	-0,700288229	0,257300337
4-androsten-3alpha,17alpha-diol monosulfate (3)		Lipid	Steroid	1,884564693	-1,255411339	-1,25539939	1,189871679	0,905375026	-0,617198513
epiandrosterone sulfate	HMDB00365	Lipid	Steroid	1,645426068	-0,888738075	-1,064440676	1,272254659	0,401806673	-1,060882188
homocitrulline	HMDB00679	Amino Acid	Urea cycle; Arginine and Proline Metabolism	-1,049796559	-0,36446311	-0,661191563	-0,693893762	-2,169035535	0,01353038
taurodeoxycholate	HMDB00896	Lipid	Secondary Bile Acid Metabolism	0,134002278	-0,043404008	0,299198949	0,460745095	-0,141519662	0,120802904
4-androsten-3beta,17beta-diol monosulfate (1)	HMDB03818	Lipid	Steroid	2,035269195	-0,95930965	0,034348904	1,000513423	1,501196844	-1,098662758
carboxyibuprofen		Xenobiotics	Drug	-0,427536723	-0,428027778	-0,430513434	-0,430203237	-0,428948766	-0,426685665
ibuprofen	HMDB01925	Xenobiotics	Drug	-0,357910107	-0,357624773	-0,356114754	-0,353892196	-0,356963996	-0,356579533
1-palmitoyl-2-oleoyl-GPE (16:0/18:1)	HMDB05320	Lipid	Phospholipid Metabolism	-1,462054352	-1,424416064	-1,282015196	-0,625689689	-1,239960718	-1,193614629
beta-alanine	HMDB00056	Nucleotide	Pyrimidine Metabolism, Uracil containing	-0,890130001	-1,14577841	-0,582888324	-1,606579289	-1,900494975	-2,069791138
docosatrienoate (22:3n3)	HMDB02823	Lipid	Polyunsaturated Fatty Acid (n3 and n6)	1,298332154	1,559331753	0,786696858	0,002184627	0,079415682	0,409294496
gamma-glutamylhistidine		Peptide	Gamma-glutamyl Amino Acid	-0,675084067	-0,883733418	0,12929657	-0,742377416	-1,270995962	-0,814094908
anserine	HMDB00194	Peptide	Dipeptide Derivative	-0,482995766	-0,483443012	-0,480927384	-0,485637214	-0,485086368	-0,482693411
2-palmitoleoyl-GPC (16:1)*		Lipid	Lysolipid	0,221440696	-1,063348984	-0,018540621	-1,060317597	-1,060976279	-1,064556029
lactose	HMDB00186	Carbohydrate	Disaccharides and Oligosaccharides	-0,534300031	-0,5330345	-0,534976682	-0,530364755	-0,534720982	-0,534836941
3-phenylpropionate (hydrocinnamate)	HMDB00764	Amino Acid	Phenylalanine and Tyrosine Metabolism	0,811125725	-1,15743356	1,143032947	1,614571795	0,704435695	1,406523063
cysteinylglycine	HMDB00078	Amino Acid	Glutathione Metabolism	0,322625463	-0,023163479	0,857945118	-0,294720629	0,121051016	0,653078175
ribonate	HMDB00867	Carbohydrate	Pentose Metabolism	-1,801906894	-1,924412381	-0,786139131	-2,265202181	-2,544430723	-1,371854602
uracil	HMDB00300	Nucleotide	Pyrimidine Metabolism, Uracil containing	-1,124134804	1,514022987	-1,121613379	-1,110951807	-0,432337342	-1,124434543
cinnamoylglycine	HMDB11621	Xenobiotics	Food Component/Plant	0,10349848	-1,162954862	1,497869586	0,475667478	1,01987512	1,264350612
N-acetyl-1-methylhistidine*		Amino Acid	Histidine Metabolism	0,379040058	0,533303199	0,507603105	0,402434677	-0,186511186	0,456366853
5,6-dihydrouracil	HMDB00076	Nucleotide	Pyrimidine Metabolism, Uracil containing	1,097888818	-0,6747313	-0,67414956	-0,661748197	1,487573975	-0,668443638
pimelate (heptanedioate)	HMDB00857	Lipid	Fatty Acid, Dicarboxylate	0,515781032	-0,637622338	0,267372408	0,875568107	0,929304595	0,261542286
3-methyladipate	HMDB00555	Lipid	Fatty Acid, Dicarboxylate	1,3665165	1,625468237	2,969338157	2,088576986	2,237226273	2,552547407
8-hydroxyoctanoate		Lipid	Fatty Acid, Monohydroxy	-0,64998354	-0,650078279	-0,656923023	-0,648839076	-0,655768314	-0,656311683
2-aminobutyrate	HMDB00650	Amino Acid	Methionine, Cysteine, SAM and Taurine Metabolism	1,849940736	0,895679731	0,841440046	0,428986772	2,342668365	0,864419957
N-acetylglucosamine/N-acetylgalactosamine		Carbohydrate	Aminosugar Metabolism	0,453475044	0,800640849	0,324327483	0,449133744	0,331753543	0,51066436
2-stearoyl-GPI (18:0)*		Lipid	Lysolipid	-1,254599748	-0,238920946	-1,256290679	-0,270073995	-0,18500542	0,248439457

4-acetamidobutanoate	HMDB03681	Amino Acid	Polyamine Metabolism	-1,426459309	0,098146033	-0,145953268	-1,552168708	-2,000945883	0,3754573
9-hydroxystearate		Lipid	Fatty Acid, Monohydroxy	-0,007866415	0,38724242	0,537999585	0,817281223	0,054733606	0,29367081
caprylate (8:0)	HMDB00482	Lipid	Medium Chain Fatty Acid	-0,710896792	-0,489026726	-0,622157539	0,984208301	-0,696543187	-0,590804524
1-oleoyl-2-linoleoyl-GPC (18:1/18:2)*		Lipid	Phospholipid Metabolism	-1,407925614	0,585555423	-1,995774088	-1,21273336	-1,377379278	-0,828807682
1-palmitoyl-2-dihomo-linolenoyl-GPC (16:0/20:3n3 or 6)*		Lipid	Phospholipid Metabolism	-0,942349325	-1,069558897	-0,510156866	-0,856874773	-1,413292053	-0,757886024
palmitic amide		Lipid	Fatty Acid, Amide	0,746333857	0,832972274	0,083546812	0,941091435	0,291452359	0,588836992
3-methoxytyramine sulfate		Amino Acid	Phenylalanine and Tyrosine Metabolism	-0,957699043	-0,240621633	-0,951958898	-0,948966756	-0,952826348	-0,950221665
N-palmitoyltaurine		Lipid	Endocannabinoid	-0,597287598	-0,588254607	2,138613929	0,791487626	-0,594851731	1,312466157
N-stearoyltaurine		Lipid	Endocannabinoid	-0,662617376	-0,667442435	2,329077273	1,714026133	0,894497365	1,865551258
3-ureidopropionate	HMDB00026	Nucleotide	Pyrimidine Metabolism, Uracil containing	0,221648535	0,169889647	0,673292085	0,213475988	0,363644351	0,432992681
glycerophosphoglycerol		Lipid	Glycerolipid Metabolism	-0,352791948	0,551019959	0,194460127	0,165855989	-0,645085787	0,480531467
1-palmitoleoyl-2-linoleoyl-GPC (16:1/18:2)*		Lipid	Phospholipid Metabolism	-0,187080941	-0,861517551	-0,648277507	-1,356943127	-2,183699147	-1,66272718
1-palmitoyl-2-alpha-linolenoyl-GPC (16:0/18:3n3)*		Lipid	Phospholipid Metabolism	-2,308033986	-1,504174365	-0,493808442	-1,046602485	-1,019931719	-0,683632521
1-pentadecanoylglycerol (15:0)		Lipid	Monoacylglycerol	-1,151198424	-1,141265173	0,729317392	0,776718482	-0,005105213	-1,149748273
gamma-glutamylphenylalanine	HMDB00594	Peptide	Gamma-glutamyl Amino Acid	0,130975882	0,052629314	0,219000887	-0,805953265	-0,137715294	-0,265674259
guanidinosuccinate	HMDB03157	Amino Acid	Guanidino and Acetamido Metabolism	-0,780188532	0,866057772	-0,785134539	-0,78425546	-0,784562108	0,696801268
N6-carboxymethyllysine		Carbohydrate	Advanced Glycation End-product	-0,441373822	-0,612711208	-0,55506764	0,04322169	-0,587642437	-0,430479555
3-methoxycatechol sulfate (1)		Xenobiotics	Benzoate Metabolism	-1,346051191	-0,1961519	-0,209971992	-1,344597853	-0,188616359	-0,220112661
N-acetylisoleucine		Amino Acid	Leucine, Isoleucine and Valine Metabolism	0,170269983	0,865326243	1,189548522	-0,772066694	-0,211266708	-0,055857755
1-stearyl-GPC (O-18:0)*		Lipid	Lyso-phospho-ether	-2,031199865	0,303654614	0,932837973	0,056710404	0,553594787	0,794320808
1-(1-enyl-stearoyl)-GPC (P-18:0) *		Lipid	Lysoplasmalogen	0,456213895	0,583350306	1,33713101	-0,018594539	1,164047166	1,320292963
1-linoleoyl-GPC (18:2)	HMDB10386	Lipid	Lysolipid	-0,357885325	-0,546315062	0,212829729	-0,051498762	-1,142385916	-0,117513096
N2,N2-dimethylguanosine	HMDB04824	Nucleotide	Purine Metabolism, Guanine containing	-0,496964835	-0,095944332	-0,07417314	-1,075285269	-1,098845864	-0,517640675
xanthurenate	HMDB00881	Amino Acid	Tryptophan Metabolism	-1,292223299	0,429514168	-0,353506618	-1,291612754	-1,294775682	-0,11726546
prolylvaline		Peptide	Dipeptide	-0,67093697	-0,666883083	-0,671908958	-0,659457417	-0,66179151	-0,665000008
2-hydroxyadipate	HMDB00321	Lipid	Fatty Acid, Dicarboxylate	-0,771708529	0,029518086	0,229123756	-0,765975293	-0,769958136	-0,770671866
2-piperidinone		Xenobiotics	Food Component/Plant	-0,266715811	0,15978067	-0,180882649	-0,29507369	-0,345774264	-0,167600521
1-margaroyl-GPC (17:0)	HMDB12108	Lipid	Lysolipid	0,705397154	-0,83551557	1,729827222	0,110115115	0,432515824	1,294521093
riboflavin (Vitamin B2)	HMDB00244	Cofactors and Vitamins	Riboflavin Metabolism	-0,675020946	-0,681240527	-0,678194172	-0,676170036	-0,683897108	-0,68462464
adrenate (22:4n6)	HMDB02226	Lipid	Polyunsaturated Fatty Acid (n3 and n6)	0,878084099	0,817969437	1,45370783	0,229459296	0,95172256	0,25145475

pyroglutamylglutamine		Peptide	Dipeptide	-1,044603324	0,705982915	-1,042744211	-1,036734014	-0,576441719	0,719927613
N-oleoyltaurine		Lipid	Endocannabinoid	-0,066096107	0,387230676	1,000088338	-0,132893744	-0,817990689	-0,055959042
4-acetaminophen sulfate	HMDB59911	Xenobiotics	Drug	-0,459984587	1,368644075	-0,461149341	-0,460404823	-0,46165971	-0,462418336
4-acetamidophenol	HMDB01859	Xenobiotics	Drug	-0,402251962	1,478188678	-0,405102526	-0,401178045	-0,403576911	-0,400885411
4-acetamidophenylglucuronide	HMDB10316	Xenobiotics	Drug	-0,382187572	1,232425276	-0,381951454	-0,383537862	-0,38623117	-0,384154006
2-hydroxyacetaminophen sulfate*		Xenobiotics	Drug	-0,376306866	1,484088905	-0,374867333	-0,377798446	-0,376502792	-0,374715108
leucylleucine	HMDB28933	Peptide	Dipeptide	-0,971067039	-0,962699017	-0,970301081	-0,972206159	-0,970488826	-0,96951172
3-methylxanthine	HMDB01886	Xenobiotics	Xanthine Metabolism	0,326855692	-0,048252886	-0,733992894	-0,73835546	1,102619676	-0,733202219
theophylline	HMDB01889	Xenobiotics	Xanthine Metabolism	0,668057934	-0,75406932	-0,752233468	-0,750560316	-0,754769317	-0,753637067
caffeine	HMDB01847	Xenobiotics	Xanthine Metabolism	0,484434525	-0,842001145	-0,842460406	-0,841955784	-0,842972204	-0,841468422
1,7-dimethylurate	HMDB11103	Xenobiotics	Xanthine Metabolism	0,701528196	-0,704780179	-0,704491301	-0,704023439	-0,702503292	-0,706393692
5-acetylamino-6-amino-3-methyluracil	HMDB04400	Xenobiotics	Xanthine Metabolism	0,858207514	-0,639155106	-0,640113229	-0,640205567	-0,397834845	-0,640966028
3,7-dimethylurate	HMDB01982	Xenobiotics	Xanthine Metabolism	-0,568471148	-0,563755131	-0,565788789	-0,571202592	-0,257566201	-0,574449701
1-palmitoyl-2-meadoyl-GPC (16:0/20:3n9)*		Lipid	Phospholipid Metabolism	0,321029911	0,394649938	0,3476752	0,320254868	0,083322622	0,879301296
alpha-ketobutyrate	HMDB00005	Amino Acid	Methionine, Cysteine, SAM and Taurine Metabolism	2,410858634	1,364629947	1,373260049	1,451842471	2,042762622	0,918609994
1-margaroyl-GPE (17:0)*		Lipid	Lysolipid	-1,040415203	-1,038565265	0,87211629	-1,041050834	-1,045137492	0,152332578
1-palmitoyl-GPA (16:0)	HMDB00327	Lipid	Lysolipid	-1,205214301	-1,200817561	0,857235158	-1,201167639	-1,208409369	0,422838987
1-arachidonoyl-GPA (20:4)		Lipid	Lysolipid	-1,084248138	-1,085232761	0,770323272	-1,08683316	-1,083709523	0,585012794
2-docosahexaenoyl-GPC (22:6)*		Lipid	Lysolipid	-1,172346358	-0,50875804	0,402336251	-0,390533748	-0,543243338	0,722544453
2-oxindole-3-acetate		Xenobiotics	Food Component/Plant	-0,666404005	0,913620669	2,187426808	-0,668442286	-0,666685611	-0,665248117
2-methylmalonyl carnitine	HMDB13133	Lipid	Fatty Acid Synthesis	-1,116996429	-1,108425759	-1,112004786	-1,112036371	-1,110314908	-1,110381487
glycylleucine	HMDB00759	Peptide	Dipeptide	-0,61697155	-0,610738628	2,839775162	-0,611388086	-0,615950422	-0,614430887
DSGEGDFXAEGGGVR*		Peptide	Fibrinogen Cleavage Peptide	-0,520570681	-0,517503181	-0,520480312	-0,51701346	1,472228154	-0,520036712
2-hydroxyphenylacetate	HMDB00669	Amino Acid	Phenylalanine and Tyrosine Metabolism	0,376521037	0,307237421	0,257264946	0,07196149	-0,395312978	-0,655740151
glycerophosphoinositol*		Lipid	Phospholipid Metabolism	-0,872468901	-0,873335823	-0,873863015	-0,875590451	-0,876090532	-0,873160974
4-acetylphenol sulfate		Xenobiotics	Drug	-0,987173283	-0,892686451	-0,978284298	-0,979548342	-0,987434582	1,055454102
umbelliferone sulfate		Xenobiotics	Food Component/Plant	-0,61923878	-0,620277995	-0,613839322	-0,613771126	-0,613017731	-0,147413998
saccharin	HMDB29723	Xenobiotics	Food Component/Plant	-0,601541742	0,267211949	1,991565904	1,261603185	0,521187101	1,470380725
isovalerate	HMDB00718	Amino Acid	Leucine, Isoleucine and Valine Metabolism	-0,813218097	-0,807343411	-0,813965633	-0,809240273	-0,807048062	-0,814739195
N-acetylkynurenine (2)		Amino Acid	Tryptophan Metabolism	-0,740327668	-0,742518585	-0,735669247	-0,737306051	-0,739380514	2,388423389
2-oleoylglycerol (18:1)		Lipid	Monoacylglycerol	-1,354703007	-1,349381959	1,298164298	-1,350283575	-1,353379911	-1,354578002

o-cresol sulfate		Amino Acid	Phenylalanine and Tyrosine Metabolism	1,231825825	-0,22160817	-0,658442764	1,335709311	-0,659361448	0,298537141
5alpha-pregnan-3(alpha or beta),20beta-diol disulfate		Lipid	Steroid	1,350159999	-0,768481713	-0,763234713	-0,766345335	-0,766894441	-0,765040898
1-stearoyl-GPS (18:0)*		Lipid	Lysolipid	-0,800691835	-0,800686689	-0,798173058	-0,80119974	-0,798594854	-0,798738023
dihydroferulic acid		Xenobiotics	Food Component/Plant	-1,112403369	-1,108555241	-1,112949044	0,929176373	-1,110629071	-1,105698576
alpha-hydroxycaproate	HMDB01624	Lipid	Fatty Acid, Monohydroxy	1,720493294	-0,769671497	1,901453122	-0,770530181	1,819572153	-0,76587565
1-arachidonylglycerol (20:4)	HMDB11572	Lipid	Monoacylglycerol	-1,044057479	-1,040044067	0,467852047	-1,04757758	-0,092042205	-0,022698242
1-linolenoylglycerol (18:3)	HMDB11569	Lipid	Monoacylglycerol	-0,824672815	-1,2437703	0,134035117	-1,245149921	-1,248863826	-1,243808005
1-dihomo-linolenylglycerol (20:3)		Lipid	Monoacylglycerol	-1,161985395	-0,523329012	-1,166195746	-1,162793102	-1,162462478	-0,055256784
caproate (6:0)	HMDB00535	Lipid	Medium Chain Fatty Acid	-1,14235922	-1,138819241	-1,143037266	1,17530583	-1,143380282	-1,143153495
anthranilate	HMDB01123	Amino Acid	Tryptophan Metabolism	-0,940279641	-0,942770484	-0,938963011	-0,94064418	-0,938384514	-0,941129876
cysteine sulfinic acid	HMDB00996	Amino Acid	Methionine, Cysteine, SAM and Taurine Metabolism	-0,745971674	-0,743522342	-0,739450857	-0,748874506	-0,737911547	-0,747469656
phenylalanylalanine		Peptide	Dipeptide	-0,927186385	-0,934575188	-0,937592545	0,740656302	0,350862648	-0,929082538
3-hydroxy-3-methylglutarate	HMDB00355	Lipid	Mevalonate Metabolism	-1,496684623	1,091541016	1,487205556	-0,208011578	0,410474241	1,639839354
N-carbamoylaspartate	HMDB00828	Nucleotide	Pyrimidine Metabolism, Orotate containing	0,534726057	-0,415985585	-0,787727611	-0,672396908	0,017136996	-0,2233771
glycylphenylalanine	HMDB28848	Peptide	Dipeptide	-0,405250196	-0,396695921	-0,384072355	-0,395043543	-0,384545336	-0,382925446
2-palmitoylglycerol (16:0)	HMDB11533	Lipid	Monoacylglycerol	0,347786687	-1,196029713	0,558139875	0,461400605	-1,195158814	-1,199036603
adipate	HMDB00448	Lipid	Fatty Acid, Dicarboxylate	-0,324462075	-0,23482405	1,048385133	0,399899978	0,951239392	0,169104896
ethyl paraben sulfate		Xenobiotics	Chemical	0,110958483	2,451216482	-0,797666843	-0,063310717	-0,274220536	0,590831581
ranitidine	HMDB01930	Xenobiotics	Drug	-0,348232928	-0,351454157	-0,348743497	-0,35013626	-0,352570026	-0,352756827
N6-carbamoylthreonyladenosine	HMDB41623	Nucleotide	Purine Metabolism, Adenine containing	-0,597873559	-0,161406683	-0,118820754	-1,110905002	-0,415471247	-0,321865223
