# Supplementary material for: Biochemical phenotyping unravels novel metabolic abnormalities and potential biomarkers associated with treatment of GLUT1 deficiency with ketogenic diet
Source: PLoS One. 2017 Sep 29;12(9):e0184022. doi: 10.1371/journal.pone.0184022 (PMC5621665; doi:10.1371/journal.pone.0184022)
Supplement: S3 Table — (PDF) [file pone.0184022.s003.PDF]

Supplemental Table 3. Complete list of Z-scores from urine samples of patients 1, 2, 3, 4 and 6 on ketogenic diet.

CHEMICAL_NAME	HMDB	SUPER_PATHWAY	SUB_PATHWAY	ID-797	ID-785	ID-787	ID-792	ID-790
glycine	HMDB00123	Amino Acid	Glycine, Serine and Threonine Metabolism	-1,078891712	0,075769581	-0,458499959	1,261362444	0,033986787
N-acetylglycine	HMDB00532	Amino Acid	Glycine, Serine and Threonine Metabolism	-0,597238179	1,724848039	3,151659687	3,980531707	1,034779987
sarcosine	HMDB00271	Amino Acid	Glycine, Serine and Threonine Metabolism	-2,143294662	0,15493415	0,288761831	0,67037105	0,530821994
dimethylglycine	HMDB00092	Amino Acid	Glycine, Serine and Threonine Metabolism	-0,491513658	-0,469627769	0,370914004	0,507983592	0,045721834
betaine	HMDB00043	Amino Acid	Glycine, Serine and Threonine Metabolism	-0,810130984	-0,544221012	-0,411756761	-0,215964761	0,574153945
serine	HMDB00187	Amino Acid	Glycine, Serine and Threonine Metabolism	-1,367676644	0,16658455	-1,072156083	0,400601267	-0,354869311
N-acetylserine	HMDB02931	Amino Acid	Glycine, Serine and Threonine Metabolism	-1,490783784	0,0452723	0,357992854	1,436369308	0,08453894
threonine	HMDB00167	Amino Acid	Glycine, Serine and Threonine Metabolism	-1,50357604	-0,468032672	-1,659201241	0,364699299	-1,694557351
N-acetylthreonine		Amino Acid	Glycine, Serine and Threonine Metabolism	-1,515264337	-2,974862065	-2,956899458	-2,958031845	-2,965051983
allo-threonine	HMDB04041	Amino Acid	Glycine, Serine and Threonine Metabolism	-0,018949405	0,150826493	-0,264514164	-0,208917846	-4,14581358
alanine	HMDB00161	Amino Acid	Alanine and Aspartate Metabolism	-3,389309473	-1,541214146	-3,303962626	-2,455222715	-1,026145776
N-acetylalanine	HMDB00766	Amino Acid	Alanine and Aspartate Metabolism	-0,95294549	0,348638501	0,875754545	0,183224155	1,190269056
aspartate	HMDB00191	Amino Acid	Alanine and Aspartate Metabolism	-1,398316252	0,045345719	-0,095534714	-0,477954737	-0,559243432
asparagine	HMDB00168	Amino Acid	Alanine and Aspartate Metabolism	-1,662892237	-0,011424998	-1,300289973	0,184669983	-1,617922064
N-acetylasparagine	HMDB06028	Amino Acid	Alanine and Aspartate Metabolism	-2,15058341	-0,218853157	-0,866035129	0,530076994	-1,42291174
N-acetylaspartate (NAA)	HMDB00812	Amino Acid	Alanine and Aspartate Metabolism	-1,732621815	0,094885374	-0,818121333	0,021263335	0,642163677
N-carbamoylalanine		Amino Acid	Alanine and Aspartate Metabolism	-0,030407459	-0,42687643	0,344793576	0,099169018	0,503201134
glutamate	HMDB00148	Amino Acid	Glutamate Metabolism	-0,876477514	0,488687355	-1,234913336	-0,978692664	0,101786666
glutamine	HMDB00641	Amino Acid	Glutamate Metabolism	-1,100444323	-0,201112668	-1,039856172	0,481840741	-1,337971859
N-acetylglutamate	HMDB01138	Amino Acid	Glutamate Metabolism	-1,461417355	0,707757237	-0,142638626	0,2824237	0,506414226
N-acetylglutamine	HMDB06029	Amino Acid	Glutamate Metabolism	-1,443383192	-0,265887596	0,673809038	1,395748512	-1,388857151
N-acetyl-aspartyl-glutamate (NAAG)	HMDB01067	Amino Acid	Glutamate Metabolism	-0,891561102	1,393999806	0,129914009	0,893544067	1,323958532
gamma-aminobutyrate (GABA)	HMDB00112	Amino Acid	Glutamate Metabolism	-0,541720589	0,435938159	0,606315666	-0,001775444	1,184712777
carboxyethyl-GABA	HMDB02201	Amino Acid	Glutamate Metabolism	-2,095883022	-0,207436615	-0,259373409	2,158309358	-0,222894428
4-hydroxyglutamate	HMDB01344	Amino Acid	Glutamate Metabolism	-0,816743453	-0,029773308	-0,723273452	0,257687135	-0,804370664
N-methylglutamate		Amino Acid	Glutamate Metabolism	0,545586239	0,08587531	0,32465096	0,099745546	1,113296877
pyroglutamine*		Amino Acid	Glutamate Metabolism	0,749696435	-0,147229742	1,040532799	1,525138311	0,105240576
N-methyl-GABA		Amino Acid	Glutamate Metabolism	-1,791164851	0,127327962	-1,785949784	-0,590395145	-1,790512917

gamma-carboxyglutamate	HMDB41900	Amino Acid	Glutamate Metabolism	0,24896036	0,741095413	-0,120886126	0,208131363	0,206193222
histidine	HMDB00177	Amino Acid	Histidine Metabolism	-0,968516823	0,346520966	-3,20362439	-0,278975064	-2,53986654
N-acetylhistidine	HMDB32055	Amino Acid	Histidine Metabolism	-2,291886839	0,538094267	-1,178412682	-0,192906396	-0,535888755
1-methylhistidine	HMDB00001	Amino Acid	Histidine Metabolism	0,927913734	1,906430663	-0,214752968	0,891449718	0,067214653
3-methylhistidine	HMDB00479	Amino Acid	Histidine Metabolism	-0,400817211	2,222205451	-0,398589189	-0,313546003	-0,290092143
N-acetyl-3-methylhistidine*		Amino Acid	Histidine Metabolism	-1,451197531	1,751107655	-0,367362062	-0,257379492	0,228526365
N-acetyl-1-methylhistidine*		Amino Acid	Histidine Metabolism	-0,321520539	0,913446263	1,442984089	1,203941364	-0,236925909
hydantoin-5-propionic acid	HMDB01212	Amino Acid	Histidine Metabolism	1,114258155	1,316409158	0,707995837	0,864649597	0,42786338
trans-urocanate	HMDB00301	Amino Acid	Histidine Metabolism	-2,304548367	1,823258111	-0,569818943	0,305791774	0,485247103
cis-urocanate		Amino Acid	Histidine Metabolism	-1,168313395	0,728123436	-0,864024876	0,432917348	0,345102582
imidazole propionate	HMDB02271	Amino Acid	Histidine Metabolism	-2,141881084	0,396092242	-1,225617647	-0,865251426	-0,041849696
imidazole lactate	HMDB02320	Amino Acid	Histidine Metabolism	-0,12168864	0,807178184	2,432563482	1,537043913	1,014521437
histamine	HMDB00870	Amino Acid	Histidine Metabolism	-0,958611128	1,166033889	0,304759467	0,490119141	-0,714032587
1-methylhistamine	HMDB00898	Amino Acid	Histidine Metabolism	-1,893775943	0,540146246	-0,713788076	0,003563627	0,11050855
1-methylimidazoleacetate	HMDB02820	Amino Acid	Histidine Metabolism	-2,324830242	0,900253285	-0,633470088	-1,373935864	-0,20302953
4-imidazoleacetate	HMDB02024	Amino Acid	Histidine Metabolism	-0,476362514	0,116246438	-0,068011447	-0,632576467	0,695241241
N-acetylhistamine	HMDB13253	Amino Acid	Histidine Metabolism	-1,275037487	1,359930262	-0,258921499	-0,271803767	1,135266196
lysine	HMDB00182	Amino Acid	Lysine Metabolism	-1,192436977	0,104414333	-1,394492524	-0,851582757	-1,003463273
N2-acetyllysine	HMDB00446	Amino Acid	Lysine Metabolism	-1,010095722	1,415455835	0,128808266	-0,238100747	0,229259862
N6-acetyllysine	HMDB00206	Amino Acid	Lysine Metabolism	-1,596691084	1,483624785	-1,051796112	0,470038149	0,053387046
N6,N6,N6-trimethyllysine	HMDB01325	Amino Acid	Lysine Metabolism	-0,333306023	1,765957068	0,176243563	0,082519811	0,017043855
5-hydroxylysine	HMDB00450	Amino Acid	Lysine Metabolism	-1,102720354	-0,034498616	-1,418058593	-0,429574365	-0,233497344
saccharopine	HMDB00279	Amino Acid	Lysine Metabolism	1,603738243	1,726957681	-0,203637471	0,92651865	-0,077600974
2-aminoadipate	HMDB00510	Amino Acid	Lysine Metabolism	0,060547987	1,021883737	-0,155299846	0,209326868	-0,36423125
2-oxoadipate	HMDB00225	Amino Acid	Lysine Metabolism	-0,246128318	0,607316086	-0,09783753	0,368254258	0,765650438
glutarate (pentanedioate)	HMDB00661	Amino Acid	Lysine Metabolism	-0,929203797	0,940716015	-0,913435001	-2,497917925	0,108378081
glutarylcarnitine (C5)	HMDB13130	Amino Acid	Lysine Metabolism	0,371000359	0,833983604	0,04796025	0,300595336	0,083440596
3-methylglutarylcarnitine (1)	HMDB00552	Amino Acid	Lysine Metabolism	-2,825556292	0,736713667	-2,832488311	-0,512457735	-2,826302173
3-methylglutarylcarnitine (2)	HMDB00552	Amino Acid	Lysine Metabolism	-0,004713248	1,583507938	0,741611913	0,877114234	-0,489405652
N-acetyl-cadaverine		Amino Acid	Lysine Metabolism	-1,306980843	-0,55535235	1,121324555	-0,155435617	-0,154411111
5-aminovalerate	HMDB03355	Amino Acid	Lysine Metabolism	-0,295825896	-0,304073686	0,226991318	-0,093517037	0,014017958
5-(galactosylhydroxy)-L-lysine		Amino Acid	Lysine Metabolism	-0,585753117	0,483850555	-0,638064429	-0,066814183	-0,025801597

N2,N6-diacetyllysine		Amino Acid	Lysine Metabolism	-2,081740152	0,60858869	0,834570089	0,928128038	-0,209226607
6-oxopiperidine-2-carboxylate		Amino Acid	Lysine Metabolism	-0,176299212	0,960872381	-0,040852377	1,177407643	0,273015094
phenylalanine	HMDB00159	Amino Acid	Phenylalanine and Tyrosine Metabolism	-1,840230105	-0,199168606	-1,837939998	-0,71443525	-1,151944448
N-acetylphenylalanine	HMDB00512	Amino Acid	Phenylalanine and Tyrosine Metabolism	-1,927228754	-0,722417069	-0,372983988	-0,458908993	0,51907756
phenyllactate (PLA)	HMDB00779	Amino Acid	Phenylalanine and Tyrosine Metabolism	-0,736129602	-0,235965675	0,615814319	-0,245431058	0,056407773
4-hydroxyphenylacetate	HMDB00020	Amino Acid	Phenylalanine and Tyrosine Metabolism	-0,804228071	1,544988083	-0,271471407	-0,511809329	1,506412597
3-hydroxyphenylacetate	HMDB00440	Amino Acid	Phenylalanine and Tyrosine Metabolism	-0,043730242	1,045179409	1,207633541	0,961676329	1,474871729
tyrosine	HMDB00158	Amino Acid	Phenylalanine and Tyrosine Metabolism	-1,184205826	-0,207062249	-1,36906328	-0,776272344	-0,785136416
N-acetyltyrosine	HMDB00866	Amino Acid	Phenylalanine and Tyrosine Metabolism	-1,650783515	-0,606096026	-0,475166509	-0,55837719	0,112938705
tyramine	HMDB00306	Amino Acid	Phenylalanine and Tyrosine Metabolism	-0,989258267	-1,287996631	-1,433816141	-0,69000602	-0,485025279
m-tyramine	HMDB04989	Amino Acid	Phenylalanine and Tyrosine Metabolism	-0,126403586	-0,321324494	-1,246237453	-0,971426288	-0,319023625
4-hydroxyphenylpyruvate	HMDB00707	Amino Acid	Phenylalanine and Tyrosine Metabolism	-0,552809241	0,235211592	0,468639246	0,407300782	0,771284106
3-(4-hydroxyphenyl)lactate	HMDB00755	Amino Acid	Phenylalanine and Tyrosine Metabolism	-1,099824187	0,28181755	0,572755604	-0,135418739	-0,575208045
phenol sulfate	HMDB60015	Amino Acid	Phenylalanine and Tyrosine Metabolism	0,169500277	1,311795034	0,612521793	0,151480217	0,048202181
p-cresol sulfate	HMDB11635	Amino Acid	Phenylalanine and Tyrosine Metabolism	0,760658159	0,590631375	0,74129983	0,683849961	0,744619483
o-cresol sulfate		Amino Acid	Phenylalanine and Tyrosine Metabolism	0,661107428	0,29495782	-0,675416636	1,33312117	0,246685569
dopamine	HMDB00073	Amino Acid	Phenylalanine and Tyrosine Metabolism	0,148613482	0,749560153	0,260151926	0,455520806	-0,371766777
vanillylmandelate (VMA)	HMDB00291	Amino Acid	Phenylalanine and Tyrosine Metabolism	-1,261411716	1,691114614	-0,48521687	0,429075208	0,164118903
3-methoxytyrosine	HMDB01434	Amino Acid	Phenylalanine and Tyrosine Metabolism	-0,669250807	-1,161350191	-1,6827737	0,228740407	-1,392169432
3-methoxytyramine	HMDB00022	Amino Acid	Phenylalanine and Tyrosine Metabolism	-0,954112876	0,639193854	0,096159876	-0,092093308	-0,176128764
3-methoxytyramine sulfate		Amino Acid	Phenylalanine and Tyrosine Metabolism	-3,053194401	0,73918316	-0,598988121	-0,961341068	0,011360504
3,4-dihydroxyphenylacetate	HMDB01336	Amino Acid	Phenylalanine and Tyrosine Metabolism	0,055400469	0,560154603	0,236670363	0,693824798	0,089672505
homovanillate (HVA)	HMDB00118	Amino Acid	Phenylalanine and Tyrosine Metabolism	-0,827768639	1,02596451	-0,258843678	0,426916475	0,60485803
homovanillate sulfate	HMDB11719	Amino Acid	Phenylalanine and Tyrosine Metabolism	-1,645037987	0,40235417	-0,743936191	-1,050521139	-0,29525265
gentisate	HMDB00152	Amino Acid	Phenylalanine and Tyrosine Metabolism	-0,712489233	0,664132032	0,868850159	0,049610357	0,823579626
phenylpropionylglycine	HMDB00860	Amino Acid	Phenylalanine and Tyrosine Metabolism	0,451491123	-0,417646183	1,141868591	1,141848462	1,44114964
3-(3-hydroxyphenyl)propionate sulfate		Amino Acid	Phenylalanine and Tyrosine Metabolism	-0,968777467	0,479199604	0,232089689	0,882836417	1,200250164
3-(3-hydroxyphenyl)propionate	HMDB00375	Amino Acid	Phenylalanine and Tyrosine Metabolism	-1,058541558	0,301732194	0,143902514	1,430832379	1,407179231
5-hydroxymethyl-2-furoic acid	HMDB02432	Amino Acid	Phenylalanine and Tyrosine Metabolism	-1,346598109	-0,211352348	-0,76155714	-1,73672626	-1,248905665
2-hydroxyphenylacetate	HMDB00669	Amino Acid	Phenylalanine and Tyrosine Metabolism	0,986957358	1,239506807	0,595908261	0,745411743	0,753955912
dopamine sulfate (1)		Amino Acid	Phenylalanine and Tyrosine Metabolism	-0,695766938	0,508916754	0,434730592	0,521345902	0,25112234
dopamine sulfate (2)		Amino Acid	Phenylalanine and Tyrosine Metabolism	-0,979534962	0,351170102	0,016036407	0,317022445	-0,228573036

p-cresol-glucuronide*	HMDB11686	Amino Acid	Phenylalanine and Tyrosine Metabolism	0,602954947	0,344261982	0,56122917	0,704166117	0,702683256
tyramine O-sulfate	HMDB06409	Amino Acid	Phenylalanine and Tyrosine Metabolism	-0,135645696	-0,258212747	-0,733884802	-1,619659144	1,972888232
vanillic alcohol sulfate		Amino Acid	Phenylalanine and Tyrosine Metabolism	1,197787902	0,437508944	0,277951467	-0,387342084	1,466563298
4-hydroxycinnamate sulfate		Amino Acid	Phenylalanine and Tyrosine Metabolism	-0,693333333	-1,228218786	0,697258794	0,739189259	-1,226533039
3,4-dihydroxyphenylacetate sulfate		Amino Acid	Phenylalanine and Tyrosine Metabolism	-0,173442779	0,763332151	0,096608985	0,690328592	0,922392093
tryptophan	HMDB00929	Amino Acid	Tryptophan Metabolism	-0,91011899	0,149356679	-0,874741442	-0,053183761	-0,679815584
N-acetyltryptophan	HMDB13713	Amino Acid	Tryptophan Metabolism	-2,15262379	-0,313827908	-0,579676796	-0,531067996	0,648900348
tryptamine	HMDB00303	Amino Acid	Tryptophan Metabolism	-0,726100725	1,730333426	-0,296866281	0,891829654	-0,182714991
indolelactate	HMDB00671	Amino Acid	Tryptophan Metabolism	-1,693159744	0,342506755	0,799442221	0,50050595	0,037623578
indoleacetate	HMDB00197	Amino Acid	Tryptophan Metabolism	-0,417792915	-0,866595461	-0,901103904	-0,505347385	1,061955024
3-indoxyl sulfate	HMDB00682	Amino Acid	Tryptophan Metabolism	0,222733168	0,654533078	0,732966767	0,600620562	0,318828055
kynurenine	HMDB00684	Amino Acid	Tryptophan Metabolism	-2,274565031	0,286207501	-1,897101808	-0,653085776	-0,782896422
kynurenate	HMDB00715	Amino Acid	Tryptophan Metabolism	-1,643263434	0,607888599	-0,92699217	-0,331516259	0,244817032
3-hydroxykynurenine	HMDB00732	Amino Acid	Tryptophan Metabolism	-1,399978174	0,394004752	-0,994937756	0,058385723	-0,723639701
3-hydroxyanthranilate	HMDB01476	Amino Acid	Tryptophan Metabolism	-0,334010971	-0,119202935	-0,81990542	-0,350743089	-0,122230217
xanthurenate	HMDB00881	Amino Acid	Tryptophan Metabolism	-0,65924911	1,550288154	-0,305592416	0,450469343	0,728916155
picolinate	HMDB02243	Amino Acid	Tryptophan Metabolism	-0,602913285	0,133152118	-0,925010502	0,047006018	-0,558474662
5-hydroxyindoleacetate	HMDB00763	Amino Acid	Tryptophan Metabolism	-1,626212817	1,083815991	1,676035803	-0,204748759	0,349978607
serotonin	HMDB00259	Amino Acid	Tryptophan Metabolism	-0,375160749	-0,424574103	-0,149193832	0,171582104	0,449444548
indoleacetylglutamine	HMDB13240	Amino Acid	Tryptophan Metabolism	-1,131748883	-0,938847499	-1,18469029	-1,242745498	-1,453458914
tryptophan betaine	HMDB61115	Amino Acid	Tryptophan Metabolism	-1,291044809	-1,289912159	-1,290028166	-0,613305667	-1,29172728
C-glycosyltryptophan		Amino Acid	Tryptophan Metabolism	-0,198896064	1,114004421	-0,67813454	0,304089459	0,225351572
N-acetylkynurenine (2)		Amino Acid	Tryptophan Metabolism	-1,524454189	-0,538395008	-1,523617686	-1,09335272	0,358027771
leucine	HMDB00687	Amino Acid	Leucine, Isoleucine and Valine Metabolism	-0,638854841	0,819096033	-0,383052511	0,425976672	-0,838995977
N-acetylleucine	HMDB11756	Amino Acid	Leucine, Isoleucine and Valine Metabolism	-1,027827983	0,043720231	1,031457644	1,430785213	0,053999875
4-methyl-2-oxopentanoate	HMDB00695	Amino Acid	Leucine, Isoleucine and Valine Metabolism	0,5328517	1,565333063	2,172177846	2,580589374	1,24961036
isovalerylglycine	HMDB00678	Amino Acid	Leucine, Isoleucine and Valine Metabolism	-0,38826558	0,536242017	0,023902782	-0,681500348	0,207593727
isovalerylcarnitine	HMDB00688	Amino Acid	Leucine, Isoleucine and Valine Metabolism	-0,058575175	-0,091838174	-0,379235914	-0,179101547	0,499930889
3-methylcrotonylglycine	HMDB00459	Amino Acid	Leucine, Isoleucine and Valine Metabolism	0,387734063	1,254295095	1,086250272	0,540428589	2,113055818
beta-hydroxyisovalerate	HMDB00754	Amino Acid	Leucine, Isoleucine and Valine Metabolism	0,299380304	1,787202902	1,722484992	1,663171467	1,800591114
beta-hydroxyisovaleroylcarnitine		Amino Acid	Leucine, Isoleucine and Valine Metabolism	-0,429406371	0,14610818	-0,400211962	0,095263797	0,079548585
3-methylglutaconate	HMDB00522	Amino Acid	Leucine, Isoleucine and Valine Metabolism	-0,465513838	3,214320021	1,854973986	1,291710301	3,068921069

alpha-hydroxyisovalerate	HMDB00407	Amino Acid	Leucine, Isoleucine and Valine Metabolism	0,352741365	0,931067245	1,831081795	1,72875883	0,768872892
methylsuccinate	HMDB01844	Amino Acid	Leucine, Isoleucine and Valine Metabolism	-0,586214819	1,744033889	0,356516727	0,207124565	0,445984623
isoleucine	HMDB00172	Amino Acid	Leucine, Isoleucine and Valine Metabolism	-0,663608235	0,828191585	0,278982295	0,467986298	-0,338942417
N-acetylisoleucine		Amino Acid	Leucine, Isoleucine and Valine Metabolism	-0,316672167	-0,599738376	1,307138854	1,236039966	0,041289013
3-methyl-2-oxobutyrate	HMDB00019	Amino Acid	Leucine, Isoleucine and Valine Metabolism	1,707125259	2,725686722	3,746535048	3,974914091	1,871689266
3-methyl-2-oxovalerate	HMDB03736	Amino Acid	Leucine, Isoleucine and Valine Metabolism	1,278447545	2,325916049	3,081901057	3,356842929	1,8568188
2-methylbutyrylcarnitine (C5)	HMDB00378	Amino Acid	Leucine, Isoleucine and Valine Metabolism	-0,247269561	-0,940679868	-0,700039025	-0,570414975	-1,506662254
2-methylbutyrylglycine	HMDB00339	Amino Acid	Leucine, Isoleucine and Valine Metabolism	0,024689601	0,700706052	0,445756926	0,390094431	1,249126874
tiglylcarnitine	HMDB02366	Amino Acid	Leucine, Isoleucine and Valine Metabolism	-0,065831337	1,185439078	-0,028663493	0,242653399	0,209439182
tigloylglycine	HMDB00959	Amino Acid	Leucine, Isoleucine and Valine Metabolism	0,365805969	-0,007078636	1,533875814	0,83855921	-0,119342902
3-hydroxy-2-ethylpropionate	HMDB00396	Amino Acid	Leucine, Isoleucine and Valine Metabolism	1,968754807	2,276096095	3,918606948	4,22868214	3,415973526
ethylmalonate	HMDB00622	Amino Acid	Leucine, Isoleucine and Valine Metabolism	-0,232636821	1,691155593	0,432094622	-0,654210687	1,003776691
valine	HMDB00883	Amino Acid	Leucine, Isoleucine and Valine Metabolism	-0,289921747	1,389100146	0,286998514	1,27745685	-0,446896247
N-acetylvaline	HMDB11757	Amino Acid	Leucine, Isoleucine and Valine Metabolism	-0,493209495	1,517620785	0,778519354	0,917036422	0,591280309
isobutyrylcarnitine	HMDB00736	Amino Acid	Leucine, Isoleucine and Valine Metabolism	-0,64007636	-0,765350635	-1,298218801	-1,08018967	-1,371773495
isobutyrylglycine	HMDB00730	Amino Acid	Leucine, Isoleucine and Valine Metabolism	-0,061249415	1,5386027	0,720373065	0,454908105	1,110352449
3-hydroxyisobutyrate	HMDB00336	Amino Acid	Leucine, Isoleucine and Valine Metabolism	-0,087933527	1,588114819	-2,678183564	-2,67626175	1,151278101
methionine	HMDB00696	Amino Acid	Methionine, Cysteine, SAM and Taurine Metabolism	-1,621601978	0,112921938	-1,110085937	-0,059420542	-0,803770216
N-acetylmethionine	HMDB11745	Amino Acid	Methionine, Cysteine, SAM and Taurine Metabolism	-2,046435896	-0,175009702	-0,377280343	0,237190944	-0,127804484
N-formylmethionine	HMDB01015	Amino Acid	Methionine, Cysteine, SAM and Taurine Metabolism	-0,571349851	0,168506478	0,814037625	0,697100229	0,750368741
methionine sulfone		Amino Acid	Methionine, Cysteine, SAM and Taurine Metabolism	-0,217470853	1,024201808	0,876562419	0,2851887	0,1810305
methionine sulfoxide	HMDB02005	Amino Acid	Methionine, Cysteine, SAM and Taurine Metabolism	-2,181354516	-1,280107029	-3,691360118	-2,620989046	-1,750744504
N-acetylmethionine sulfoxide		Amino Acid	Methionine, Cysteine, SAM and Taurine Metabolism	-3,067421704	-0,765592136	-0,136255656	-0,13519248	-1,535125174
S-adenosylmethionine (SAM)	HMDB01185	Amino Acid	Methionine, Cysteine, SAM and Taurine Metabolism	-0,898288261	0,785453028	-0,179408452	0,763567397	-0,098955934
S-adenosylhomocysteine (SAH)	HMDB00939	Amino Acid	Methionine, Cysteine, SAM and Taurine Metabolism	-0,48112336	0,493513295	0,128931556	0,106576281	-0,320238362
cystathionine	HMDB00099	Amino Acid	Methionine, Cysteine, SAM and Taurine Metabolism	-0,955883175	-0,09484547	-1,834427715	-1,54159521	-1,283908811
cysteine	HMDB00574	Amino Acid	Methionine, Cysteine, SAM and Taurine Metabolism	-0,280457365	0,904196512	0,190283383	0,245464236	0,511249578
N-acetylcysteine	HMDB01890	Amino Acid	Methionine, Cysteine, SAM and Taurine Metabolism	-2,192141443	1,514868713	-0,00715563	0,991459515	1,609867117
cystine	HMDB00192	Amino Acid	Methionine, Cysteine, SAM and Taurine Metabolism	-0,776781784	-1,144234101	-1,679557251	-6,418158892	-2,05264059
S-methylcysteine	HMDB02108	Amino Acid	Methionine, Cysteine, SAM and Taurine Metabolism	0,112157856	-0,684936713	0,09506556	-0,624875196	-2,001500835

cysteine s-sulfate	HMDB00731	Amino Acid	Methionine, Cysteine, SAM and Taurine Metabolism	0,291634817	0,337884426	-0,313247592	1,069061676	-0,504353398
hypotaurine	HMDB00965	Amino Acid	Methionine, Cysteine, SAM and Taurine Metabolism	-0,00134218	1,350941302	0,582727187	0,992881689	0,480295051
taurine	HMDB00251	Amino Acid	Methionine, Cysteine, SAM and Taurine Metabolism	0,475996956	1,119662806	-0,293164591	0,451227	-0,244021339
N-acetyltaurine		Amino Acid	Methionine, Cysteine, SAM and Taurine Metabolism	-0,698551478	1,143264897	0,426685775	0,792700205	1,668379212
N-methyltaurine		Amino Acid	Methionine, Cysteine, SAM and Taurine Metabolism	-0,48319978	-0,486216708	-0,484864027	-0,485624962	-0,485670915
taurocyamine	HMDB03584	Amino Acid	Methionine, Cysteine, SAM and Taurine Metabolism	-0,754279594	0,885859289	1,611928353	0,922573088	0,504564636
2-hydroxybutyrate/2-hydroxyisobutyrate		Amino Acid	Methionine, Cysteine, SAM and Taurine Metabolism	0,576422551	0,778405317	1,186211306	1,082580291	0,537448894
arginine	HMDB00517	Amino Acid	Urea cycle; Arginine and Proline Metabolism	-2,772167924	-0,395075836	-1,057531651	-1,428256901	-0,344827213
urea	HMDB00294	Amino Acid	Urea cycle; Arginine and Proline Metabolism	-0,162326642	1,296048258	-0,331447123	0,017291704	0,523852336
ornithine	HMDB03374	Amino Acid	Urea cycle; Arginine and Proline Metabolism	-0,801924579	-0,503589535	-0,294314087	-0,619235327	-0,629758762
proline	HMDB00162	Amino Acid	Urea cycle; Arginine and Proline Metabolism	-1,524973577	-0,773531748	-1,329344147	-0,893527317	-0,596731342
citrulline	HMDB00904	Amino Acid	Urea cycle; Arginine and Proline Metabolism	-1,312709697	-0,512117172	-1,069741159	-0,901116916	-1,146222119
argininosuccinate	HMDB00052	Amino Acid	Urea cycle; Arginine and Proline Metabolism	1,164518297	1,205183582	0,10615513	-1,126992997	1,118208644
homoarginine	HMDB00670	Amino Acid	Urea cycle; Arginine and Proline Metabolism	-2,081828874	1,235894109	-1,801861994	-1,364338156	-4,5043191
homocitrulline	HMDB00679	Amino Acid	Urea cycle; Arginine and Proline Metabolism	-1,073271031	0,629643931	-1,866939885	-1,19828504	-0,248400834
N-acetylarginine	HMDB04620	Amino Acid	Urea cycle; Arginine and Proline Metabolism	-0,784726339	0,016676209	0,045221351	0,375262168	-0,167822636
N-acetylproline		Amino Acid	Urea cycle; Arginine and Proline Metabolism	0,079058638	0,662338419	2,261336664	0,742633732	1,102598955
N-delta-acetylornithine		Amino Acid	Urea cycle; Arginine and Proline Metabolism	-0,110482305	1,413921086	-1,121057852	-2,608199465	-2,610305863
N2,N5-diacetylornithine		Amino Acid	Urea cycle; Arginine and Proline Metabolism	-0,742703722	0,488465612	0,204327644	-0,12512908	0,223929537
N-alpha-acetylornithine	HMDB03357	Amino Acid	Urea cycle; Arginine and Proline Metabolism	0,204547846	0,496431444	1,748345241	0,626431941	0,288181541
N-methylproline		Amino Acid	Urea cycle; Arginine and Proline Metabolism	-0,171202145	-0,335018274	-0,069257633	-0,123860354	1,720676206
3-hydroxyproline	HMDB02113	Amino Acid	Urea cycle; Arginine and Proline Metabolism	-0,319505387	-0,520329846	0,301740271	0,772164616	0,569828844
trans-4-hydroxyproline	HMDB00725	Amino Acid	Urea cycle; Arginine and Proline Metabolism	-1,039468438	-0,51110418	-1,197274357	-0,585234377	0,229690826
pro-hydroxy-pro	HMDB06695	Amino Acid	Urea cycle; Arginine and Proline Metabolism	-1,059859485	0,532605074	-0,230697175	0,044326051	0,115938041
N-acetylcitrulline	HMDB00856	Amino Acid	Urea cycle; Arginine and Proline Metabolism	-1,4647761	0,177221502	-0,456043348	0,151406825	-0,195556432
creatine	HMDB00064	Amino Acid	Creatine Metabolism	-1,833484811	1,173145052	0,12776986	-0,474229746	0,659938136
creatinine	HMDB00562	Amino Acid	Creatine Metabolism	NA	NA	NA	NA	NA
N-methylhydantoin	HMDB03646	Amino Acid	Creatine Metabolism	-1,059962454	-1,059950578	-1,064500284	0,240972781	1,367987499
guanidinoacetate	HMDB00128	Amino Acid	Creatine Metabolism	-0,958245399	0,981049022	0,717177612	1,14160916	0,507759277
acisoga		Amino Acid	Polyamine Metabolism	0,429747432	0,777169711	0,603192395	-2,079209285	0,212340904
spermidine	HMDB01257	Amino Acid	Polyamine Metabolism	2,107313002	0,052063566	-1,114393494	0,486275333	-1,721150723

5-methylthioadenosine (MTA)	HMDB01173	Amino Acid	Polyamine Metabolism	-1,918993962	-0,215844335	-1,520488897	-0,477066473	0,082411255
N-acetylputrescine	HMDB02064	Amino Acid	Polyamine Metabolism	-2,247352867	0,641215703	-0,929283959	-0,356498734	-0,16199921
4-acetamidobutanoate	HMDB03681	Amino Acid	Polyamine Metabolism	-1,900163739	0,606952228	-0,718605215	-0,783253246	0,683683201
1-methylguanidine	HMDB01522	Amino Acid	Guanidino and Acetamido Metabolism	-0,675552447	0,95517026	1,20496577	0,391102424	0,755073803
4-guanidinobutanoate	HMDB03464	Amino Acid	Guanidino and Acetamido Metabolism	-0,960793042	0,193537515	0,638474538	-0,81162882	-1,159325908
guanidinosuccinate	HMDB03157	Amino Acid	Guanidino and Acetamido Metabolism	-0,071361806	1,536911884	0,657634639	0,35646786	1,049935456
cysteinylglycine	HMDB00078	Amino Acid	Glutathione Metabolism	-1,33294918	0,789661115	-0,628339523	-0,420594473	0,926865632
5-oxoproline	HMDB00267	Amino Acid	Glutathione Metabolism	-0,136224367	2,268760649	-0,666752117	-0,711094218	0,778958269
gamma-glutamylglutamine	HMDB11738	Peptide	Gamma-glutamyl Amino Acid	-1,03402495	-0,355022381	-1,383096733	-0,474112733	-0,244392747
gamma-glutamylglycine	HMDB11667	Peptide	Gamma-glutamyl Amino Acid	-0,979282063	0,178480639	-1,462474795	0,326670635	0,162512381
gamma-glutamylhistidine		Peptide	Gamma-glutamyl Amino Acid	-1,035073686	0,345507673	-2,026198815	0,445154429	-0,069713377
gamma-glutamylisoleucine*	HMDB11170	Peptide	Gamma-glutamyl Amino Acid	0,857222426	1,531393893	0,981375852	0,966250464	-0,161660112
gamma-glutamylleucine	HMDB11171	Peptide	Gamma-glutamyl Amino Acid	-0,099171501	0,964149948	-1,820850874	0,358734408	0,251131509
gamma-glutamyl-epsilon-lysine	HMDB03869	Peptide	Gamma-glutamyl Amino Acid	-0,555152429	0,461867924	-1,315261056	-0,400690716	-0,256077756
gamma-glutamylphenylalanine	HMDB00594	Peptide	Gamma-glutamyl Amino Acid	-0,951328665	0,612094427	-1,796796279	-0,194804475	-0,436897376
gamma-glutamyltyrosine	HMDB11741	Peptide	Gamma-glutamyl Amino Acid	-1,092334798	0,356918442	-2,126922729	-0,057925629	-0,394006676
gamma-glutamylvaline	HMDB11172	Peptide	Gamma-glutamyl Amino Acid	0,598806893	1,224625366	-0,280238255	0,289556188	0,459402855
carnosine	HMDB00033	Peptide	Dipeptide Derivative	-0,912135728	0,059950483	-1,461559546	-1,177155068	0,538495966
N-acetylcarnosine	HMDB12881	Peptide	Dipeptide Derivative	2,01902815	1,107871838	0,906787422	1,666412898	0,219074512
homocarnosine	HMDB00745	Peptide	Dipeptide Derivative	-1,643484098	0,867610655	-0,35564904	-0,126129254	1,134182726
anserine	HMDB00194	Peptide	Dipeptide Derivative	-0,847416482	1,382183117	-1,274780006	-0,47005836	0,721171301
cyclo(his-pro)		Peptide	Dipeptide	-0,624579861	-0,182061717	-0,067641537	-1,010566396	0,20321633
glycylproline	HMDB00721	Peptide	Dipeptide	-1,796217499	1,28765863	-0,595497463	0,084258341	0,632739017
leucylproline	HMDB11175	Peptide	Dipeptide	-0,466424324	1,779517967	0,170340992	0,320913866	0,537329842
phenylalanylglycine		Peptide	Dipeptide	-1,319464788	0,781043624	1,836421051	-0,239748849	1,487938369
phenylalanylproline		Peptide	Dipeptide	-0,585457023	1,444779842	-0,100420556	0,85830691	0,5793889
prolylglutamate		Peptide	Dipeptide	-2,133877238	0,95853491	-0,044445279	0,03510199	0,774917094
prolylglycine		Peptide	Dipeptide	-2,266484557	1,349935723	0,245100588	0,577511203	0,903550204
prolylproline		Peptide	Dipeptide	-1,048115914	1,537609984	-0,175740006	0,193679047	1,12097052
pyroglutamylglutamine		Peptide	Dipeptide	-0,728421469	1,384034576	-0,173245718	-0,973765662	0,807477071
pyroglutamylvaline		Peptide	Dipeptide	-1,832929849	2,378008677	0,983417096	0,20488196	-0,268344798
valylleucine	HMDB29131	Peptide	Dipeptide	-0,586555759	1,375553948	0,986026745	0,732432285	-0,767913413

phenylacetylglutamine	HMDB06344	Peptide	Acetylated Peptides	0,121509798	0,401114277	0,265506971	-0,055970929	0,173192634
phenylacetylglycine	HMDB00821	Peptide	Acetylated Peptides	0,657313204	-0,382584597	0,69159711	0,133316535	-1,797781578
4-hydroxyphenylacetyl glycine		Peptide	Acetylated Peptides	-0,251289855	0,384032597	0,577166928	1,517862194	0,522709759
phenylacetylphenylalanine		Peptide	Acetylated Peptides	-0,420606478	0,714760653	1,135668526	-0,364402891	0,13007495
1,5-anhydroglucitol (1,5-AG)	HMDB02712	Carbohydrate	Glycolysis, Gluconeogenesis, and Pyruvate Metabolism	-1,286583012	-0,697175496	-1,00929965	-1,029737372	-1,11187621
glucose	HMDB00122	Carbohydrate	Glycolysis, Gluconeogenesis, and Pyruvate Metabolism	-0,470520405	-0,596010136	-0,893350207	-0,614135501	-0,745626862
3-phosphoglycerate	HMDB00807	Carbohydrate	Glycolysis, Gluconeogenesis, and Pyruvate Metabolism	0,831443891	0,86902999	-0,194163104	-0,55247794	-0,04542401
lactate	HMDB00190	Carbohydrate	Glycolysis, Gluconeogenesis, and Pyruvate Metabolism	-0,771365352	0,100579187	0,755712758	1,39485627	-0,139653261
glycerate	HMDB00139	Carbohydrate	Glycolysis, Gluconeogenesis, and Pyruvate Metabolism	-0,412528743	0,03641813	0,5025926	0,680055691	0,498014154
ribitol	HMDB00508	Carbohydrate	Pentose Metabolism	-2,051286587	-0,986150242	-1,761355631	0,742562914	-1,84504122
ribonate	HMDB00867	Carbohydrate	Pentose Metabolism	-0,762568692	0,809493866	-0,673649947	0,063727842	0,715595267
xylose	HMDB00098	Carbohydrate	Pentose Metabolism	-1,26421867	-0,522954677	-0,495647746	2,041597798	-0,510090198
arabinose	HMDB00646	Carbohydrate	Pentose Metabolism	-1,666118023	0,721454421	-0,223460927	2,020012662	-0,004669381
arabitol/xylitol		Carbohydrate	Pentose Metabolism	-0,596020772	0,895533783	0,628423151	0,698918346	1,077393798
arabonate/xylonate		Carbohydrate	Pentose Metabolism	-2,123426045	-0,647102568	-2,043713848	-0,105091318	-0,057835049
lactose	HMDB00186	Carbohydrate	Disaccharides and Oligosaccharides	-0,124600376	-0,825484278	-0,457110918	-1,396418745	-0,872974129
3-sialyllactose	HMDB00825	Carbohydrate	Disaccharides and Oligosaccharides	0,313401587	0,746743697	-3,653069836	-2,662049395	-0,339753856
sucrose	HMDB00258	Carbohydrate	Disaccharides and Oligosaccharides	-0,748305548	0,134729361	-0,122863936	-1,144404308	-0,849062634
fructose	HMDB00660	Carbohydrate	Fructose, Mannose and Galactose Metabolism	-1,66965468	-0,674469197	-0,45994844	-0,438189891	-1,667707099
mannitol/sorbitol	HMDB00247	Carbohydrate	Fructose, Mannose and Galactose Metabolism	-1,329831357	-0,987610315	-0,407885106	-0,134946749	-0,742424811
galactitol (dulcitol)	HMDB00107	Carbohydrate	Fructose, Mannose and Galactose Metabolism	0,190277964	0,361165179	-0,176144087	0,068493396	-0,029341342
galactonate	HMDB00565	Carbohydrate	Fructose, Mannose and Galactose Metabolism	-0,115992127	0,190158443	-0,380708837	-1,096831277	0,920952066
glucuronate	HMDB00127	Carbohydrate	Aminosugar Metabolism	-4,22805301	-3,602334141	-2,553816409	-3,6765951	-0,636490227
N-acetylneuraminate	HMDB00230	Carbohydrate	Aminosugar Metabolism	-1,595051944	0,459708187	-0,68065985	-0,930211236	0,629161751
3'-a-sialyl-N-acetyllactosamine		Carbohydrate	Aminosugar Metabolism	0,246478441	1,054869461	-1,3056053	-1,235479175	0,399363155
6-sialyl-N-acetyllactosamine	HMDB06584	Carbohydrate	Aminosugar Metabolism	0,458417343	0,626936825	-0,119443836	-0,002378067	0,259353195
N-acetylglucosaminylasparagine	HMDB00489	Carbohydrate	Aminosugar Metabolism	-0,129759337	1,444760595	-0,14680497	1,145523864	0,609139946
erythronate*	HMDB00613	Carbohydrate	Aminosugar Metabolism	-0,594408588	0,511238159	-0,053080021	-0,470725095	0,700939699
N6-carboxymethyllysine		Carbohydrate	Advanced Glycation End-product	0,173017106	-0,186974954	0,031389564	1,007339643	0,368576026
citrate	HMDB00094	Energy	TCA Cycle	0,012299768	-0,227210299	0,680931057	1,123603991	0,902567404
isocitrate	HMDB00193	Energy	TCA Cycle	-0,014235571	0,28961781	0,886892151	0,720100248	0,583885834

alpha-ketoglutarate	HMDB00208	Energy	TCA Cycle	0,063727348	0,310004086	0,512705161	0,77986201	1,067665525
succinylcarnitine		Energy	TCA Cycle	-1,138416223	1,46904319	0,248486027	0,520682534	-0,925645812
succinate	HMDB00254	Energy	TCA Cycle	-3,4747086	-4,511521157	-4,387059581	-0,518871056	0,280082189
fumarate	HMDB00134	Energy	TCA Cycle	0,000136187	0,552877846	1,54631203	1,335027164	1,469884085
malate	HMDB00156	Energy	TCA Cycle	-0,704490987	0,141370963	1,178283365	1,906886928	1,376749262
tricarballylate	HMDB31193	Energy	TCA Cycle	0,569780555	0,725919575	0,750215856	-0,021655868	0,557006804
2-methylcitrate	HMDB00379	Energy	TCA Cycle	-0,513950385	-0,204697615	-1,004072445	-0,729639096	-0,820191389
citraconate/glutaconate		Energy	TCA Cycle	-1,42892662	-0,254770237	-0,060926301	-0,852219977	0,487388564
phosphate	HMDB01429	Energy	Oxidative Phosphorylation	0,304454294	1,401238673	0,706437944	-0,050454091	-0,276779213
dimethylmalonic acid	HMDB02001	Lipid	Fatty Acid, Dicarboxylate	-1,159486928	1,946012619	-0,652265724	-0,416811794	-0,001872459
2-hydroxyglutarate	HMDB00606	Lipid	Fatty Acid, Dicarboxylate	-0,275153887	1,113026053	0,532370953	0,901140097	1,796621687
adipate	HMDB00448	Lipid	Fatty Acid, Dicarboxylate	0,241276025	0,811320146	1,471081792	1,202324958	1,139125049
2-hydroxyadipate	HMDB00321	Lipid	Fatty Acid, Dicarboxylate	0,219540496	2,974124189	-0,091508812	-4,009975243	0,263410468
3-methyladipate	HMDB00555	Lipid	Fatty Acid, Dicarboxylate	0,851044592	1,947816869	2,384721969	1,747806894	2,361330312
maleate	HMDB00176	Lipid	Fatty Acid, Dicarboxylate	-0,853536374	0,600484919	0,260729375	0,35729724	0,550319579
pimelate (heptanedioate)	HMDB00857	Lipid	Fatty Acid, Dicarboxylate	0,280499783	0,51995431	1,037727822	1,516074488	0,292048555
suberate (octanedioate)	HMDB00893	Lipid	Fatty Acid, Dicarboxylate	0,033034792	0,406816329	1,441670994	1,33489122	0,423055406
4-octenedioate	HMDB04982	Lipid	Fatty Acid, Dicarboxylate	-0,495516615	1,551624676	2,146751355	1,966754658	0,647788298
azelate (nonanedioate)	HMDB00784	Lipid	Fatty Acid, Dicarboxylate	0,378863067	0,223646093	0,532788334	1,19703508	0,430565557
sebacate (decanedioate)	HMDB00792	Lipid	Fatty Acid, Dicarboxylate	0,571981088	-0,236578716	1,000199677	1,283034998	0,569563839
3-carboxy-4-methyl-5-propyl-2-furanpropanoate (CMPF)	HMDB61112	Lipid	Fatty Acid, Dicarboxylate	-0,208753665	0,393817241	0,931901927	1,247044584	1,831696805
2-aminooctanoate	HMDB00991	Lipid	Fatty Acid, Amino	-1,244287228	0,638821549	-0,934396662	-0,618067155	-1,245547477
malonylcarnitine	HMDB02095	Lipid	Fatty Acid Synthesis	-0,551545169	0,666822306	0,448492142	-0,452900378	-0,331030563
malonate	HMDB00691	Lipid	Fatty Acid Synthesis	-0,562280365	2,616977022	1,217840969	0,985947173	0,901868064
2-methylmalonyl carnitine	HMDB13133	Lipid	Fatty Acid Synthesis	-0,36807046	1,000352626	-0,319186472	0,226131272	-0,080101364
propionylcarnitine	HMDB00824	Lipid	Fatty Acid Metabolism (also BCAA Metabolism)	-0,149285114	0,526804286	0,736099299	0,752094698	-0,060396946
methylmalonate (MMA)	HMDB00202	Lipid	Fatty Acid Metabolism (also BCAA Metabolism)	-4,999381227	-4,451967546	-4,989576577	-6,83362021	-6,376305022
hexanoylglycine	HMDB00701	Lipid	Fatty Acid Metabolism(Acyl Glycine)	0,738975885	1,385503505	2,162647635	1,680209948	2,012245597
acetylcarnitine	HMDB00201	Lipid	Fatty Acid Metabolism(Acyl Carnitine)	0,676700904	-0,311827287	-0,550277103	-0,059123181	-1,247451182
3-hydroxybutyrylcarnitine (1)	HMDB13127	Lipid	Fatty Acid Metabolism(Acyl Carnitine)	0,912255597	1,040454426	0,417531652	0,720462123	0,073580704
hexanoylcarnitine	HMDB00705	Lipid	Fatty Acid Metabolism(Acyl Carnitine)	-0,579437682	0,387396876	0,060703915	0,26743409	-0,329710224
octanoylcarnitine	HMDB00791	Lipid	Fatty Acid Metabolism(Acyl Carnitine)	-0,248717772	0,977202039	-0,020481915	0,515745983	-0,345999207

decanoylcarnitine	HMDB00651	Lipid	Fatty Acid Metabolism(Acyl Carnitine)	1,164106205	1,846584797	1,737102693	2,395031901	0,618262594
myristoleoylcarnitine*		Lipid	Fatty Acid Metabolism(Acyl Carnitine)	1,736030775	1,561110454	1,934591326	1,38368527	1,663869568
deoxycarnitine	HMDB01161	Lipid	Carnitine Metabolism	-1,183794043	-1,079158853	-1,392698369	-1,462383024	-1,13191816
carnitine	HMDB00062	Lipid	Carnitine Metabolism	-0,827000847	-1,888052967	-2,174656886	-1,866700661	-2,176366709
3-hydroxybutyrate (BHBA)	HMDB00357	Lipid	Ketone Bodies	3,635767108	4,930328039	6,362288689	6,185277941	4,085023756
2-hydroxyoctanoate	HMDB02264	Lipid	Fatty Acid, Monohydroxy	-0,588883033	0,456009883	-1,289978478	-0,410920325	-1,292280821
3-hydroxysuberate	HMDB00325	Lipid	Fatty Acid, Monohydroxy	0,072806373	1,520835293	1,323010604	1,173889979	0,802769587
3-hydroxyoctanoate	HMDB01954	Lipid	Fatty Acid, Monohydroxy	-1,182195539	0,994932123	0,826435849	-0,022825926	0,876914134
3-hydroxysebacate	HMDB00350	Lipid	Fatty Acid, Monohydroxy	0,153309984	1,177713536	1,482001057	1,663648785	1,421897223
5-hydroxyhexanoate	HMDB00525	Lipid	Fatty Acid, Monohydroxy	0,484956543	0,776303217	1,431749224	1,422044051	1,111876546
epiandrosterone glucuronide		Lipid	Fatty Acid, Monohydroxy	3,074004212	-0,771312685	0,759728573	1,284875074	-0,77131642
myo-inositol	HMDB00211	Lipid	Inositol Metabolism	-0,484545019	0,988797301	-0,451042152	-0,688934384	-0,438988898
chiro-inositol	HMDB34220	Lipid	Inositol Metabolism	-0,885672282	-0,005636884	-0,884184916	0,795689123	1,840055166
scyllo-inositol	HMDB06088	Lipid	Inositol Metabolism	-0,254580385	1,116198921	0,891455585	-0,441011571	0,912479594
choline	HMDB00097	Lipid	Phospholipid Metabolism	-1,467772889	0,134076073	-0,010210438	-0,254525577	-0,862611126
choline phosphate	HMDB01565	Lipid	Phospholipid Metabolism	0,0958815	1,374405324	-0,561240479	-0,116547877	0,23258766
glycerophosphorylcholine (GPC)	HMDB00086	Lipid	Phospholipid Metabolism	-0,55920763	1,105943187	-0,286517432	0,747083832	-1,315169696
phosphoethanolamine	HMDB00224	Lipid	Phospholipid Metabolism	0,678759489	0,835385096	0,045732072	-0,189523795	0,350344909
glycerophosphoethanolamine	HMDB00114	Lipid	Phospholipid Metabolism	-0,858111018	1,423350134	-0,778055253	-0,275955288	-0,519531291
trimethylamine N-oxide	HMDB00925	Lipid	Phospholipid Metabolism	0,407475157	0,54607201	0,311830942	0,012240507	1,229721108
glycerophosphoinositol*		Lipid	Phospholipid Metabolism	-0,308688459	2,156562975	1,17570465	0,122338515	0,973750443
glycerol 3-phosphate	HMDB00126	Lipid	Glycerolipid Metabolism	0,598687708	1,34535698	0,459378249	-0,125843096	0,669366317
glycerophosphoglycerol		Lipid	Glycerolipid Metabolism	-0,971240116	1,586585627	-0,649258406	1,413306659	0,471395493
3-hydroxy-3-methylglutarate	HMDB00355	Lipid	Mevalonate Metabolism	-0,395563377	0,751260816	1,165106482	-0,335496871	0,89012574
21-hydroxypregnenolone disulfate	HMDB04026	Lipid	Steroid	0,677567616	-1,035388796	-0,582217586	0,42961227	-0,951805156
5alpha-pregnan-3beta,20alpha-diol disulfate		Lipid	Steroid	0,876414255	-0,911684823	-0,911716791	0,175678217	-0,91155742
5alpha-pregnan-3(alpha or beta),20beta-diol disulfate		Lipid	Steroid	-0,639515636	-0,639450866	-0,636280486	-0,639239116	-0,637779524
pregnen-diol disulfate*		Lipid	Steroid	1,002806369	-1,478489282	-1,027611278	0,300133282	-1,772784204
pregnanediol-3-glucuronide		Lipid	Steroid	2,080118074	0,20000198	0,312191339	1,525186142	0,01644474
cortisone	HMDB02802	Lipid	Steroid	0,213098562	-0,606181043	-0,668956741	-1,393892441	0,458488246
tetrahydrocortisone	HMDB00903	Lipid	Steroid	0,796409562	0,063711652	0,705950577	1,158247408	-1,77520363
dehydroisoandrosterone sulfate (DHEA-S)	HMDB01032	Lipid	Steroid	1,203067362	-0,672747647	-0,672718918	-0,669496109	-0,671193185

16a-hydroxy DHEA 3-sulfate		Lipid	Steroid	1,384109801	-1,191756539	-1,194698377	-1,194657569	-1,192607542
androsterone sulfate	HMDB02759	Lipid	Steroid	1,728213176	-0,423903303	-0,543389447	1,901626753	0,321927808
4-androsten-3beta,17beta-diol disulfate (1)	HMDB03818	Lipid	Steroid	1,790320486	-0,966063968	-0,660686287	1,014231702	-0,96632241
4-androsten-3beta,17beta-diol disulfate (2)	HMDB03818	Lipid	Steroid	1,265225163	-1,218278999	-1,046220064	0,333814472	-1,529233069
andro steroid monosulfate (1)*	HMDB02759	Lipid	Steroid	1,080959079	-1,212674074	-0,199943736	-0,331290848	-1,21171703
11-ketoetiocholanolone glucuronide		Lipid	Steroid	1,570903947	-0,525614267	0,713058064	1,269203259	-0,181704111
etiocholanolone glucuronide		Lipid	Steroid	2,677917024	0,593135088	0,562993398	2,069211687	0,146424535
17alpha-hydroxypregnanolone glucuronide		Lipid	Steroid	2,157122242	0,602966207	0,856541194	1,198869299	0,247337642
5beta-pregnan-3alpha,21-diol-11,20-dione 21-glucosiduronate		Lipid	Steroid	0,786989679	1,713525015	0,480596578	0,475589748	1,206480045
cholate	HMDB00619	Lipid	Primary Bile Acid Metabolism	-1,5089887	1,356035743	-0,808531574	0,061417311	-0,344406176
glycocholate	HMDB00138	Lipid	Primary Bile Acid Metabolism	-0,600710326	-0,35558066	-0,16342942	-0,298197818	-0,502662143
glycochenodeoxycholate	HMDB00637	Lipid	Primary Bile Acid Metabolism	-0,774901658	-0,917215492	-0,912069601	-0,91500588	-0,913208626
tauro-beta-muricholate	HMDB00932	Lipid	Primary Bile Acid Metabolism	1,489898156	0,98980765	1,868123661	1,045114612	0,864015201
glycolithocholate sulfate*	HMDB02639	Lipid	Secondary Bile Acid Metabolism	1,707502033	-0,148530835	1,217743992	1,297235314	-1,252567454
taurolithocholate 3-sulfate	HMDB02580	Lipid	Secondary Bile Acid Metabolism	1,980226352	-0,776180549	0,730051742	1,540886846	-0,139093544
glycoursodeoxycholate	HMDB00708	Lipid	Secondary Bile Acid Metabolism	-0,024542944	0,299640726	-0,314005937	0,58328318	-1,274145751
glycohyocholate		Lipid	Secondary Bile Acid Metabolism	0,196335447	0,06033633	0,027798563	0,100672143	-0,089085329
12-dehydrocholate	HMDB00400	Lipid	Secondary Bile Acid Metabolism	-0,700249743	1,297749034	0,542831052	1,398593206	0,499038165
glycocholenate sulfate*		Lipid	Secondary Bile Acid Metabolism	1,68061579	1,228110334	-0,783198396	1,157590965	-1,148398825
taurocholenate sulfate		Lipid	Secondary Bile Acid Metabolism	0,856487138	0,413171715	-0,594770209	0,54038519	-0,186461956
7-ketodeoxycholate	HMDB00391	Lipid	Secondary Bile Acid Metabolism	-0,854483298	0,872597128	0,389537882	0,742522998	0,67763935
inosine	HMDB00195	Nucleotide	Purine Metabolism, (Hypo)Xanthine/Inosine containing	-1,03490609	-0,219895462	-0,943917427	-0,103871068	0,361806319
hypoxanthine	HMDB00157	Nucleotide	Purine Metabolism, (Hypo)Xanthine/Inosine containing	-0,184380273	-0,221407499	-0,283029605	0,063604713	0,976801447
xanthine	HMDB00292	Nucleotide	Purine Metabolism, (Hypo)Xanthine/Inosine containing	-1,162814497	0,345458836	-0,240472507	0,470745504	0,78179204
xanthosine	HMDB00299	Nucleotide	Purine Metabolism, (Hypo)Xanthine/Inosine containing	-1,53436716	0,800531072	-0,459451843	-0,790350177	1,49595114
urate	HMDB00289	Nucleotide	Purine Metabolism, (Hypo)Xanthine/Inosine containing	-0,758586938	-0,62185375	-1,770269302	-1,040728802	-0,132601104
allantoin	HMDB00462	Nucleotide	Purine Metabolism, (Hypo)Xanthine/Inosine containing	-1,836092178	-0,574202361	-0,981724242	-0,971861887	-0,687460784
allantoic acid	HMDB01209	Nucleotide	Purine Metabolism, (Hypo)Xanthine/Inosine containing	-1,348001547	1,148642007	1,568635182	1,041724866	-1,401011577
adenosine 3',5'-cyclic monophosphate (cAMP)	HMDB00058	Nucleotide	Purine Metabolism, Adenine containing	-1,207906149	3,123746991	0,333501938	1,957431002	1,113812303
adenosine	HMDB00050	Nucleotide	Purine Metabolism, Adenine containing	-0,25945515	0,535946435	-0,256996764	1,016236613	1,718307664
adenine	HMDB00034	Nucleotide	Purine Metabolism, Adenine containing	-0,26207389	-0,746327516	-0,94720651	-0,939660999	0,974043111

1-methyladenine	HMDB11599	Nucleotide	Purine Metabolism, Adenine containing	-1,212998924	-2,733908094	-1,217067946	-2,736490613	-0,172213192
N1-methyladenosine	HMDB03331	Nucleotide	Purine Metabolism, Adenine containing	-1,655735126	0,9185914	0,340192583	0,718303612	-0,093747902
N6-methyladenosine	HMDB04044	Nucleotide	Purine Metabolism, Adenine containing	-0,76044546	0,685551371	-0,48221244	-0,016544078	0,936336346
N6-carbamoylthreonyladenosine	HMDB41623	Nucleotide	Purine Metabolism, Adenine containing	-0,457148499	1,332161128	-0,531195034	0,265138007	0,186085056
N6-succinyladenosine	HMDB00912	Nucleotide	Purine Metabolism, Adenine containing	-1,725897622	1,233106319	-0,537345319	-1,839893206	0,088486345
guanosine-3',5'-cyclic monophosphate (cGMP)	HMDB01314	Nucleotide	Purine Metabolism, Guanine containing	-1,386622933	1,21167272	-0,447426499	0,470343688	1,17929892
guanosine	HMDB00133	Nucleotide	Purine Metabolism, Guanine containing	-1,107036121	-1,448400009	-1,705733585	-0,051591824	0,04471783
guanine	HMDB00132	Nucleotide	Purine Metabolism, Guanine containing	1,198476797	0,47397605	1,194418961	1,401635701	1,1559163
7-methylguanine	HMDB00897	Nucleotide	Purine Metabolism, Guanine containing	-0,769609594	-0,986656384	-1,165559594	-0,475751405	0,726597717
N1-methylguanosine	HMDB01563	Nucleotide	Purine Metabolism, Guanine containing	-0,222036823	0,980110179	-0,218906697	0,274557697	-1,035272158
N2-methylguanosine	HMDB05862	Nucleotide	Purine Metabolism, Guanine containing	-0,549829427	1,193726506	-0,665283656	0,836744084	1,025892543
N2,N2-dimethylguanosine	HMDB04824	Nucleotide	Purine Metabolism, Guanine containing	-1,029441792	-0,099744919	-0,943927307	-0,728735543	-0,129302343
N2,N2-dimethylguanine		Nucleotide	Purine Metabolism, Guanine containing	-0,752830844	0,411725936	0,237539365	0,230825683	0,853759605
2'-deoxyguanosine	HMDB00085	Nucleotide	Purine Metabolism, Guanine containing	0,138195488	-0,044408196	-0,420356249	0,869192359	0,757526172
N-carbamoylaspartate	HMDB00828	Nucleotide	Pyrimidine Metabolism, Orotate containing	2,232200981	2,610179941	0,911993655	-0,173782062	1,157284121
orotate	HMDB00226	Nucleotide	Pyrimidine Metabolism, Orotate containing	-0,708510204	-1,496668759	-1,332460522	-0,60735461	-1,253373335
orotidine	HMDB00788	Nucleotide	Pyrimidine Metabolism, Orotate containing	-0,554807709	1,837313474	-0,840029696	0,711303283	0,438933466
uridine	HMDB00296	Nucleotide	Pyrimidine Metabolism, Uracil containing	-0,143399942	0,601623384	-0,913256013	0,449999011	-0,43800694
uracil	HMDB00300	Nucleotide	Pyrimidine Metabolism, Uracil containing	0,110055843	0,923372758	-0,512497754	-0,20901774	0,062829031
pseudouridine	HMDB00767	Nucleotide	Pyrimidine Metabolism, Uracil containing	-0,594673051	1,561932331	-0,346971982	0,358281578	0,664642346
N3-methyluridine	HMDB04813	Nucleotide	Pyrimidine Metabolism, Uracil containing	-0,352871755	1,055646125	-0,609439045	0,393937491	0,209522369
5,6-dihydrouracil	HMDB00076	Nucleotide	Pyrimidine Metabolism, Uracil containing	-0,886900498	2,220722389	1,510086146	-0,554514657	0,227943027
4-ureidobutyrate		Nucleotide	Pyrimidine Metabolism, Uracil containing	-0,057312697	0,37223731	-0,745395754	-0,123988	0,557291472
3-ureidopropionate	HMDB00026	Nucleotide	Pyrimidine Metabolism, Uracil containing	-0,923952975	-0,123553486	-0,30201993	0,189966664	-0,401503047
N-acetyl-beta-alanine		Nucleotide	Pyrimidine Metabolism, Uracil containing	-0,059955136	0,622517676	1,745522486	1,180355632	2,021888509
cytidine	HMDB00089	Nucleotide	Pyrimidine Metabolism, Cytidine containing	-1,117270269	-0,285350722	-0,394461018	-0,490250316	0,058621972
cytosine	HMDB00630	Nucleotide	Pyrimidine Metabolism, Cytidine containing	-2,647872538	-0,277891455	-0,987030511	-2,647062947	0,710608831
3-methylcytidine		Nucleotide	Pyrimidine Metabolism, Cytidine containing	-0,876962001	-0,066942612	-1,184975533	-0,628314983	1,096004037
N4-acetylcytidine	HMDB05923	Nucleotide	Pyrimidine Metabolism, Cytidine containing	-0,680300663	1,364245273	-0,613396342	0,396178395	0,410011886
thymine	HMDB00262	Nucleotide	Pyrimidine Metabolism, Thymine containing	0,229391603	1,382416924	-0,156198392	0,292023747	0,318392284
5,6-dihydrothymine	HMDB00079	Nucleotide	Pyrimidine Metabolism, Thymine containing	-0,936983249	0,853181819	-0,687898671	0,243765083	0,221729044
3-aminoisobutyrate	HMDB03911	Nucleotide	Pyrimidine Metabolism, Thymine containing	-0,05783726	1,837335815	1,227737443	0,82740119	0,798506523

quinolinate	HMDB00232	Cofactors and Vitamins	Nicotinate and Nicotinamide Metabolism	-0,835574584	1,11648827	-1,352644162	-0,715136967	-0,261163038
nicotinate ribonucleoside	HMDB06809	Cofactors and Vitamins	Nicotinate and Nicotinamide Metabolism	-0,033895675	1,086185674	0,670052147	0,253228369	0,414485005
nicotinamide	HMDB01406	Cofactors and Vitamins	Nicotinate and Nicotinamide Metabolism	0,503481933	-0,706821874	-0,831687191	-1,159778576	-1,055315313
nicotinamide riboside	HMDB00855	Cofactors and Vitamins	Nicotinate and Nicotinamide Metabolism	0,518688878	0,372138858	1,033013414	1,112837774	-0,633287881
1-methylnicotinamide	HMDB00699	Cofactors and Vitamins	Nicotinate and Nicotinamide Metabolism	0,286320444	-0,327675254	-1,420213204	-0,624342752	-0,532246903
trigonelline (N'-methylnicotinate)	HMDB00875	Cofactors and Vitamins	Nicotinate and Nicotinamide Metabolism	-1,435112666	0,126823327	0,205186442	-0,441970216	0,337608415
N1-Methyl-2-pyridone-5-carboxamide	HMDB04193	Cofactors and Vitamins	Nicotinate and Nicotinamide Metabolism	0,101808532	0,354462184	-2,811731863	-0,863776944	-0,730981903
riboflavin (Vitamin B2)	HMDB00244	Cofactors and Vitamins	Riboflavin Metabolism	0,385188772	-0,455496052	-0,892843864	-0,556270978	0,159941267
pantothenate	HMDB00210	Cofactors and Vitamins	Pantothenate and CoA Metabolism	0,29255452	0,736035252	-1,032139292	0,025895441	0,379935951
glucarate (saccharate)	HMDB00663	Cofactors and Vitamins	Ascorbate and Aldarate Metabolism	1,242339547	0,801517058	0,76401761	-0,368925457	1,322690677
ascorbate (Vitamin C)	HMDB00044	Cofactors and Vitamins	Ascorbate and Aldarate Metabolism	0,022673752	-1,579172342	-1,577505465	-1,154408552	-1,579705501
dehydroascorbate	HMDB01264	Cofactors and Vitamins	Ascorbate and Aldarate Metabolism	0,35295981	-0,731279325	-0,346229587	-0,526011464	-0,155526754
threonate	HMDB00943	Cofactors and Vitamins	Ascorbate and Aldarate Metabolism	-1,040426962	-0,342205378	-0,712463035	-1,017024185	-0,720801836
oxalate (ethanedioate)	HMDB02329	Cofactors and Vitamins	Ascorbate and Aldarate Metabolism	-0,481132236	0,538109059	-0,871218906	-1,961554066	-0,269282074
gulonate*	HMDB03290	Cofactors and Vitamins	Ascorbate and Aldarate Metabolism	-0,394356013	1,536191305	-0,080992752	-0,039593368	0,793245143
gamma-CEHC	HMDB01931	Cofactors and Vitamins	Tocopherol Metabolism	-1,309801263	-0,523737439	-0,491136132	-1,311686811	-1,314522975
gamma-CEHC glucuronide*		Cofactors and Vitamins	Tocopherol Metabolism	-0,927087189	0,122378807	-0,204955086	-0,571357347	-0,416944385
alpha-CEHC glucuronide*		Cofactors and Vitamins	Tocopherol Metabolism	0,239630217	1,677097047	0,74948905	1,153211989	0,127742404
alpha-CEHC sulfate		Cofactors and Vitamins	Tocopherol Metabolism	0,059735936	1,395723215	0,560355783	0,749538992	0,149851105
biopterin	HMDB00468	Cofactors and Vitamins	Tetrahydrobiopterin Metabolism	-0,670283454	1,575228439	1,02811305	0,856716241	0,222275371
dihydrobiopterin	HMDB00038	Cofactors and Vitamins	Tetrahydrobiopterin Metabolism	-0,425392531	0,961068083	0,808013446	1,598789017	0,403781268
isoxanthopterin	HMDB00704	Cofactors and Vitamins	Pterin Metabolism	-2,674249691	-2,667026952	0,425003624	-2,663753647	0,373225735
neopterin	HMDB00845	Cofactors and Vitamins	Pterin Metabolism	0,067427249	0,95096862	0,617173991	0,188429465	-0,283799221
7,8-dihydroneopterin	HMDB02275	Cofactors and Vitamins	Pterin Metabolism	0,610664308	0,353398932	0,503648473	0,712205809	-1,262981302
5-aminolevulinate	HMDB01149	Cofactors and Vitamins	Hemoglobin and Porphyrin Metabolism	-0,30186711	0,985101945	1,132668715	0,631833809	0,628984865
L-urobilin	HMDB04159	Cofactors and Vitamins	Hemoglobin and Porphyrin Metabolism	3,830922512	-0,506289694	-0,502810872	0,653013659	0,643637352
thiamin (Vitamin B1)	HMDB00235	Cofactors and Vitamins	Thiamine Metabolism	-0,271210614	-0,174503737	-2,224326684	-0,871717599	-0,316491072

pyridoxamine	HMDB01431	Cofactors and Vitamins	Vitamin B6 Metabolism	-0,636980692	-0,011562467	-0,647778768	-0,100452409	0,445986691
pyridoxal	HMDB01545	Cofactors and Vitamins	Vitamin B6 Metabolism	-1,178065867	-0,536485721	-1,380136478	-0,762702143	-0,307192164
pyridoxate	HMDB00017	Cofactors and Vitamins	Vitamin B6 Metabolism	-0,914393894	0,166485349	-1,149633191	-0,692432583	0,640817737
hippurate	HMDB00714	Xenobiotics	Benzoate Metabolism	-0,421567467	-0,405260683	0,802690959	0,519513558	0,571260057
2-hydroxyhippurate (salicylurate)	HMDB00840	Xenobiotics	Benzoate Metabolism	-0,533878473	0,727444284	1,152297397	-0,441318343	-0,135692587
3-hydroxyhippurate	HMDB06116	Xenobiotics	Benzoate Metabolism	-0,64190268	0,504289089	0,046276877	1,048555803	0,849478267
4-hydroxyhippurate	HMDB13678	Xenobiotics	Benzoate Metabolism	-1,757720893	1,199044675	-0,457725309	-0,491696036	2,171077341
mandelate	HMDB00703	Xenobiotics	Benzoate Metabolism	-0,134578994	1,121561479	0,142356525	1,302233332	1,555465071
4-hydroxymandelate	HMDB00822	Xenobiotics	Benzoate Metabolism	0,823954814	1,506663264	0,557504358	1,150026136	1,212262441
4-hydroxybenzoate	HMDB00500	Xenobiotics	Benzoate Metabolism	-0,457566471	1,098911631	0,11389318	0,063848939	2,316744606
2,4,6-trihydroxybenzoate		Xenobiotics	Benzoate Metabolism	-1,444136299	0,553159578	1,408356302	0,603293548	0,527862475
catechol sulfate	HMDB59724	Xenobiotics	Benzoate Metabolism	-0,409460362	0,796849508	0,487181181	0,502061172	0,790793527
O-methylcatechol sulfate		Xenobiotics	Benzoate Metabolism	-0,478250804	0,679648508	0,411230136	0,362681959	0,420377967
3-methyl catechol sulfate (1)		Xenobiotics	Benzoate Metabolism	0,979374523	-0,095333364	-0,305754494	1,478173037	-0,071035486
3-methyl catechol sulfate (2)		Xenobiotics	Benzoate Metabolism	0,358757006	-0,862413102	-0,861538891	0,748963045	-0,859108306
4-methylcatechol sulfate		Xenobiotics	Benzoate Metabolism	0,343659675	1,131043213	0,714746379	0,91016164	0,900755408
4-ethylphenylsulfate		Xenobiotics	Benzoate Metabolism	1,302160856	0,316433393	0,755536129	0,632063579	0,159532604
4-vinylphenol sulfate	HMDB04072	Xenobiotics	Benzoate Metabolism	-0,550101273	0,13111964	0,005348706	0,247981463	-0,398498592
benzene-1,2,3-triol		Xenobiotics	Benzoate Metabolism	-0,222184217	0,974796476	-1,216976511	-1,215787894	0,516087479
3-methoxycatechol sulfate (1)		Xenobiotics	Benzoate Metabolism	-1,125405521	0,485458285	-0,104702822	-0,047384786	0,435443868
3-methoxycatechol sulfate (2)		Xenobiotics	Benzoate Metabolism	-1,530349273	0,983103118	-0,23515575	1,044924201	0,543405957
methyl-4-hydroxybenzoate sulfate		Xenobiotics	Benzoate Metabolism	-0,334932882	-0,185027105	-1,804370229	-1,305938663	1,248100152
caffeine	HMDB01847	Xenobiotics	Xanthine Metabolism	-0,031054439	-0,772022558	0,564924606	-0,768626018	-0,773872615
paraxanthine	HMDB01860	Xenobiotics	Xanthine Metabolism	0,774625022	-0,782106897	1,017748221	-0,776801878	-0,782999883
theobromine	HMDB02825	Xenobiotics	Xanthine Metabolism	0,814023966	0,238019407	1,438260188	-0,915154632	-1,345020655
theophylline	HMDB01889	Xenobiotics	Xanthine Metabolism	0,749702306	-0,742099834	0,287106166	-0,740533208	-0,745064049
1-methylurate	HMDB03099	Xenobiotics	Xanthine Metabolism	-0,387581627	-0,691778412	1,226271538	-2,253853654	-2,18870911
7-methylurate		Xenobiotics	Xanthine Metabolism	0,424309363	0,954600025	1,905920733	-0,538043186	-0,985661862
1,3-dimethylurate	HMDB01857	Xenobiotics	Xanthine Metabolism	1,344174253	-0,799914352	1,801814292	-0,794635736	-0,79731821
1,7-dimethylurate	HMDB11103	Xenobiotics	Xanthine Metabolism	0,756690162	-0,109068453	1,140016777	-1,184538909	-1,186495268
3,7-dimethylurate	HMDB01982	Xenobiotics	Xanthine Metabolism	1,014509946	0,821994637	2,134252521	-0,308358217	-0,942332324
1,3,7-trimethylurate	HMDB02123	Xenobiotics	Xanthine Metabolism	0,716954301	0,344046806	1,62817206	-0,962176629	-0,965222999

1-methylxanthine	HMDB10738	Xenobiotics	Xanthine Metabolism	0,743374272	-0,074351991	1,314768639	-1,002807017	-1,286677881
3-methylxanthine	HMDB01886	Xenobiotics	Xanthine Metabolism	1,182607166	0,715931756	1,603388377	0,178395281	-0,086929416
7-methylxanthine	HMDB01991	Xenobiotics	Xanthine Metabolism	0,358081753	0,570033471	1,493909972	-0,550933248	-1,546766749
5-acetylamino-6-amino-3-methyluracil	HMDB04400	Xenobiotics	Xanthine Metabolism	0,673611791	0,471855226	1,364370718	0,405548159	-0,298387031
3-methylurate*		Xenobiotics	Xanthine Metabolism	0,08475694	1,116590341	2,314374947	-0,807473305	-0,94693994
piperidine	HMDB34301	Xenobiotics	Food Component/Plant	0,072195225	-0,39020042	0,819643343	-0,070485956	0,53506696
2-piperidinone		Xenobiotics	Food Component/Plant	-0,598074219	0,320345349	-0,145020598	-0,145402158	0,205540347
sucralose	HMDB31554	Xenobiotics	Food Component/Plant	1,193414459	1,609781429	0,618748139	-0,63039412	-0,157563792
1,1-kestotetraose	HMDB39176	Xenobiotics	Food Component/Plant	-0,51420798	-0,514856087	-0,515370292	-0,515251952	-0,513461072
levulinate (4-oxovalerate)	HMDB00720	Xenobiotics	Food Component/Plant	-0,281134273	1,037361235	1,285283134	0,869172467	0,532900446
vanillate	HMDB00484	Xenobiotics	Food Component/Plant	1,294966505	0,349036522	0,782250764	-0,66105261	2,653476217
1,6-anhydroglucose	HMDB00640	Xenobiotics	Food Component/Plant	-1,884425669	-1,528698597	-2,433621229	-1,930797816	-1,155977841
2,3-dihydroxyisovalerate	HMDB12141	Xenobiotics	Food Component/Plant	-0,66986856	0,878925303	1,158606112	-0,20622594	0,901607434
2-isopropylmalate	HMDB00402	Xenobiotics	Food Component/Plant	-0,70895588	-0,032573537	0,727047827	0,24801679	-0,143860815
2-oxindole-3-acetate		Xenobiotics	Food Component/Plant	-0,248679975	1,340325253	1,719645112	0,65906328	0,202239582
3,5-dihydroxybenzoic acid	HMDB13677	Xenobiotics	Food Component/Plant	0,189246908	0,722443614	1,02307616	1,682431893	1,029031143
3-hydroxyindolin-2-one		Xenobiotics	Food Component/Plant	1,287167918	-0,300975215	0,887147779	0,213702053	-1,542105408
betonicine	HMDB29412	Xenobiotics	Food Component/Plant	-1,232470471	1,021112885	1,009172773	1,157670176	-1,23195747
gluconate	HMDB00625	Xenobiotics	Food Component/Plant	-0,331365232	-0,430233834	-1,239392085	-0,941890816	-1,109832119
abscisate	HMDB35140	Xenobiotics	Food Component/Plant	0,270502009	0,907328264	2,554413153	2,516672676	1,253433361
N-acetylalliin		Xenobiotics	Food Component/Plant	-1,033968229	-1,033752576	-1,032962942	-0,428601769	-1,033071768
3-hydroxycinnamate (m-coumarate)	HMDB01713	Xenobiotics	Food Component/Plant	-1,064177782	-0,160541431	-0,058720942	1,556077792	1,955121127
cinnamoylglycine	HMDB11621	Xenobiotics	Food Component/Plant	0,330416203	-0,228035377	1,448405993	1,322201929	1,887299618
daidzein	HMDB03312	Xenobiotics	Food Component/Plant	-0,539191059	-0,536909375	-0,539981426	-0,537283842	-0,531527012
dihydroferulic acid		Xenobiotics	Food Component/Plant	-0,87957028	-0,247463323	-0,110062436	0,832389561	0,675400728
erythritol	HMDB02994	Xenobiotics	Food Component/Plant	3,050577394	2,741124226	-1,137290001	-0,070101482	-0,707091123
ferulate	HMDB00954	Xenobiotics	Food Component/Plant	-0,823232035	-0,820130441	0,888749786	0,097690789	0,832386972
ferulic acid 4-sulfate	HMDB29200	Xenobiotics	Food Component/Plant	-0,611570841	-0,388158888	0,799516169	-0,048231616	-0,077478056
fucitol		Xenobiotics	Food Component/Plant	-0,686588095	-0,110596174	-0,223408558	-0,162000972	-0,419985521
homocitrate	HMDB03518	Xenobiotics	Food Component/Plant	2,262720343	2,651750664	3,35197977	2,15306749	2,850361344
homostachydrine*	HMDB33433	Xenobiotics	Food Component/Plant	0,113971874	-0,61891612	-0,08018945	-0,845454375	0,650684421
indolin-2-one		Xenobiotics	Food Component/Plant	0,153388836	-0,007674195	0,933103818	0,389736331	0,354623532

methyl indole-3-acetate	HMDB29738	Xenobiotics	Food Component/Plant	0,226518095	0,237083777	-0,6544295	0,25422693	0,599679171
N-(2-furoyl)glycine	HMDB00439	Xenobiotics	Food Component/Plant	0,016987886	-1,009452952	-1,061769252	-1,675343981	-1,786938704
quinate	HMDB03072	Xenobiotics	Food Component/Plant	-0,940404821	-0,104160818	1,072781336	0,129838902	-0,275123533
saccharin	HMDB29723	Xenobiotics	Food Component/Plant	0,34051146	1,075121941	1,833255258	1,217135693	1,570375879
stachydrine	HMDB04827	Xenobiotics	Food Component/Plant	0,181765171	-0,77236493	-0,581507861	0,156652612	1,298476251
syringic acid	HMDB02085	Xenobiotics	Food Component/Plant	-0,588500104	0,571866702	1,536682921	1,21595424	-1,046418678
tartarate	HMDB00956	Xenobiotics	Food Component/Plant	-0,885826504	1,639656943	1,89909765	-2,500369378	-0,797321074
thymol sulfate	HMDB01878	Xenobiotics	Food Component/Plant	0,014483829	0,599217984	-0,991358819	-0,990508744	-0,991809426
4-allylphenol sulfate		Xenobiotics	Food Component/Plant	-0,411924822	-0,200330306	-0,450557544	0,369651888	0,404177158
N-acetyl-S-allyl-L-cysteine		Xenobiotics	Food Component/Plant	-0,792266493	-0,791784042	-0,793684209	-0,794079771	-0,793604109
4-vinylguaiacol sulfate		Xenobiotics	Food Component/Plant	-0,903104448	-0,374198109	-0,903450529	-0,905534068	-0,905517006
pyrraline		Xenobiotics	Food Component/Plant	-0,526987809	1,096884249	-0,080655962	-0,324326039	0,688979553
umbelliferone sulfate		Xenobiotics	Food Component/Plant	-0,562153827	-0,392567838	-1,077375672	-1,46848733	-0,077555323
eugenol sulfate		Xenobiotics	Food Component/Plant	-0,200317129	0,227721187	-0,104963053	-0,256493505	-0,410027097
N-acetylpyrraline		Xenobiotics	Food Component/Plant	-0,914709024	0,992074291	-0,074067286	-0,150805452	0,312373934
2-keto-3-deoxy-gluconate	HMDB01353	Xenobiotics	Food Component/Plant	0,571413775	0,305981715	-1,65129382	0,236451411	0,048225291
3-hydroxycinnamate sulfate		Xenobiotics	Food Component/Plant	-0,914562828	0,181248528	0,210200915	0,98832962	1,355688777
syringol sulfate		Xenobiotics	Food Component/Plant	-0,540970507	1,685567917	0,412316757	1,662185792	-0,057298733
4-acetaminophen sulfate	HMDB59911	Xenobiotics	Drug	-0,610178459	1,573726095	-0,888277536	-1,155891227	-1,155584162
salicyluric glucuronide*		Xenobiotics	Drug	-0,64071871	0,974229237	1,76813528	-1,300764818	-1,729476808
4-acetylphenol sulfate		Xenobiotics	Drug	-0,188810073	0,266680114	0,273548515	-1,740312817	0,448496259
4-hydroxycoumarin		Xenobiotics	Drug	-0,506524948	-0,512757024	-0,509315039	-0,174765525	-0,508594214
hydroquinone sulfate	HMDB02434	Xenobiotics	Drug	-0,283718938	1,099635073	0,571038647	1,016134269	0,171782225
pivaloylcarnitine	HDMB41993	Xenobiotics	Drug	0,522888906	1,040868368	-0,923593634	0,067801504	1,388572709
salicylate	HMDB01895	Xenobiotics	Drug	-0,440694131	0,001927433	1,024428239	-1,162041226	0,710976382
S-carboxymethyl-L-cysteine	HMDB29415	Xenobiotics	Drug	-2,06154726	0,564167669	-0,116207735	-0,26375073	-0,336618748
2-acetamidophenol sulfate		Xenobiotics	Drug	-0,77263297	0,207342394	0,568162226	1,082427213	0,729939501
diglycerol		Xenobiotics	Chemical	1,432933085	0,536102811	-0,311321785	0,45923993	1,443497655
sulfate*	HMDB01448	Xenobiotics	Chemical	-0,192956026	0,430351296	-0,724799195	-0,079232026	1,012013019
O-sulfo-L-tyrosine		Xenobiotics	Chemical	-0,552075225	2,185979679	0,290036778	0,915378791	0,111855677
2-oxo-1-pyrrolidinepropionate		Xenobiotics	Chemical	0,253165033	0,139750364	5,106348043	0,93146766	1,315152613
2-aminophenol sulfate	HMDB61116	Xenobiotics	Chemical	-0,742463672	0,181192521	0,684200052	1,013295228	0,90172486

S-(3-hydroxypropyl)mercapturic acid (HPMA)		Xenobiotics	Chemical	-1,108994133	0,247760108	-0,3251301	-0,712139598	0,968604357
dimethyl sulfone	HMDB04983	Xenobiotics	Chemical	-1,938297453	-0,639405029	0,036044801	0,336240267	-1,937437181
ectoine		Xenobiotics	Chemical	-0,998183088	-0,105348316	-0,117242025	-0,462966838	-0,86223376
benzoylcarnitine*		Xenobiotics	Chemical	0,350305523	0,464641594	0,849166365	0,799538253	0,527000417
succinimide		Xenobiotics	Chemical	-0,345248937	1,152034267	1,241968729	0,168159292	0,483906861
N-methylpipecolate		Xenobiotics	Chemical	-0,155249757	1,521189296	0,500781832	2,377422886	0,432153375
1,2,3-benzenetriol sulfate (2)		Xenobiotics	Chemical	-0,119457345	0,822191735	0,024237374	-0,323473526	0,574165917
2-methoxyresorcinol sulfate		Xenobiotics	Chemical	-0,26198133	0,949017221	-0,046013557	-0,766332992	0,519892585
3-hydroxypyridine sulfate		Xenobiotics	Chemical	-0,170295331	-0,096789965	0,872891955	-0,401588677	-0,340306378
1,2,3-benzenetriol sulfate (1)		Xenobiotics	Chemical	-0,218425265	0,910718497	-0,15489243	-0,530965886	0,649872572
3-hydroxyindolin-2-one sulfate		Xenobiotics	Chemical	0,343397548	0,193429314	1,157843017	0,519195416	0,53243586
gentisic acid-5-glucoside		Xenobiotics	Chemical	-0,306534764	1,105325087	1,223170648	0,776842669	-0,884053241
4'-hydroxypropiophenone sulfate		Xenobiotics	Chemical	-1,042909139	0,332606673	-1,048528792	-1,042684799	-1,045358014
